# Investigation of the Lipid-Lowering Mechanisms and Active Ingredients of Danhe Granule on Hyperlipidemia Based on Systems Pharmacology

**DOI:** 10.3389/fphar.2020.00528

**Published:** 2020-05-06

**Authors:** Kuikui Chen, Zhaochen Ma, Xiaoning Yan, Jie Liu, Wenjuan Xu, Yueting Li, Yihang Dai, Yinhuan Zhang, Hongbin Xiao

**Affiliations:** ^1^Research Center of Chinese Medicine Analysis and Transformation & School of Chinese Materia Medica, Beijing University of Chinese Medicine, Beijing, China; ^2^School of Life Sciences, Beijing University of Chinese Medicine, Beijing, China

**Keywords:** Danhe granule, hyperlipidemia, systems pharmacology, mechanism, active ingredients

## Abstract

**Objective:**

Investigate the active ingredients and underlying hypolipidemic mechanisms of Danhe granule (DHG).

**Methods:**

The lipid-lowering effect of DHG was evaluated in hyperlipidemic hamsters induced by a high-fat diet. The ingredients absorbed into the blood after oral administration of DHG in hamsters were identified by ultra-high-performance liquid chromatography coupled with quadrupole time-of-flight mass spectrometry (UHPLC-Q-TOF/MS). A systems pharmacology approach incorporating target prediction and network construction, gene ontology (GO) enrichment and pathway analysis was performed to predict the active compounds and map the compounds-targets-disease network. Real-time polymerase chain reaction (RT-PCR) and Western blot were utilized to analyze the mRNA and protein expression levels of predicted targets.

**Results:**

DHG remarkably lowered the levels of serum total cholesterol (TC), triglyceride (TG), low-density lipoprotein cholesterol (LDL-c), and arteriosclerosis index (AI), at the same time, elevated the levels of serum high-density lipoprotein cholesterol (HDL-c) and HDL-c/TC ratio in hyperlipidemic hamsters. Sixteen ingredients absorbed into blood after oral administration of DHG were identified as the possible components interacted with targets. Moreover, 65 potential targets were predicted after targets intersection and compounds–targets–disease network mapping. Then, compounds–targets–pathways network mapping revealed that six active compounds (emodin, naringenin, etc.) compounds could interact with 10 targets such as sterol regulatory element binding protein (SREBP) 1c, SREBP-2 and peroxisome proliferation-activated receptor (PPAR) α, regulate three lipid metabolism-related pathways including SREBP control of lipid synthesis pathway, PPAR signaling pathway and nuclear receptors in lipid metabolism and toxicity pathway, and further affect lipid metabolic processes including fatty acid biosynthesis, low-density lipoprotein receptor (LDLR)-mediated cholesterol uptake, bile acid biosynthesis, and cholesterol efflux. Experimental results indicated that DHG significantly increased SREBP-2, LDLR, PPARα, liver X receptor alpha (LXRα), cholesterol 7α-hydroxylase (CYP7A1), and ATP binding cassette subfamily A member 1 (ABCA1) mRNA and protein expressions while decreased SREBP-1c and fatty acid synthase (FAS) mRNA, and protein expressions.

**Conclusion:**

DHG possessed a good hypolipidemic effect that may be through affecting the mRNA and protein expressions of SREBP-1c, FAS, SREBP-2, LDLR, PPARα, LXRα, CYP7A1, and ABCA1, involving in fatty acid synthesis, LDLR-mediated cholesterol uptake, bile acid biosynthesis, and cholesterol efflux. This study further provided experimental evidence about its practical application for treating hyperlipidemia and its complications.

## Introduction

Cardiovascular diseases (CVDs) are the leading cause of death globally. Hyperlipidemia is one of the leading risk factors for the development and progression of CVDs, characterized by elevated serum total cholesterol (TC), serum triglycerides (TG), low-density lipoprotein cholesterol (LDL-c), and decreased serum high-density lipoprotein cholesterol (HDL-c) (Gupta et al., 2011). It is well known that reducing high lipid levels, especially for the circulating levels of TC and LDL-c, has a huge potential to lower the risks for CVDs (Ridker, 2014; Qu et al., 2018). Genetic background, environmental factors, and lifestyle preferences are the main factors that may contribute to the rising prevalence of hyperlipidemia (Mathur and Devi, 2016). In some cases, patients with hyperlipidemia also suffered from severe complications such as coronary artery disease, ischemic stroke, angina pectoris, and diabetes. The commonly used lipid-regulating drugs like statins, the inhibitors of the rate-limiting enzyme in cholesterol synthesis, are challenging to achieve a satisfactory therapeutic effect, but also accompanied by harmful side effects such as gastrointestinal tract issues and myopathy (Garnett, 1995; Tacherfiout et al., 2018). It was reported that the proportion of patients with myopathy caused by statins was about 10%, and an estimated 20% of the patients were statin-resistant or intolerant (Harper and Jacobson, 2007; Maningat and Breslow, 2011). Thus, it warrants to find alternative medicines for the treatment of hyperlipidemia.

In several Asian countries, such as China and Korea, herbs and traditional Chinese medicine (TCM) formulas were widely used to prevent and cure atherosclerosis and hyperlipidemia. Danhe granule (DHG) is a TCM formula which consists of *Salvia miltiorrhiza* Bunge (Danshen), *Reynoutria japonica* Houtt. (Huzhang), *Crataegus pinnatifida* Bunge (Shanzha), *Citrus × aurantium* L. (Chenpi), *Coix lacryma-jobi var. ma-yuen* (Rom.Caill.) Stapf (Yiyiren), and *Nelumbo nucifera* Gaertn. (Heye). It is originated from clinical prescriptions Danhe decoction that has been used in treating hyperlipidemia for many years. Modern pharmacology researches showed that some component herbs of DHG exhibited excellent hypolipidemic effects. For example, *Crataegus pinnatifida* Bge. could reduce blood lipid levels by inhibiting cholesterol biosynthesis and increasing lipid β oxidation (Ye et al., 2010; Niu et al., 2011). Meanwhile, researches also showed some bioactive monomer compounds such as naringin and salvianolic acid B possessed regulatory effects on lipid metabolism disorder (Yue et al., 2015; Liang et al., 2016). Although previous studies indicated that DHG had potential effects on hyperlipidemia (Ma et al., 2019), because of the complexity of components, the underlying lipid-lowering mechanisms, and effective components of DHG are not yet clear.

In TCM formula, the characteristics “multi-component, multi-target, and multi-pathway” present a tremendous challenge in understanding of the interactions between components and their mechanisms of action (Jiang et al., 2019). Fortunately, systems pharmacology, as a new discipline based on the basic theories of pharmacology and systems biology pharmacology, integrating pharmacology feature mapping, multiple targeting techniques, network pharmacology, and pathway analyses, has gradually become a powerful tool to investigate the therapeutic mechanisms of TCM (Su et al., 2019; Zhou et al., 2019). For example, Liu et al. found 33 compounds with potential anticancer effects from *Scutellaria barbata* D. Don and investigated their mechanisms in treating non-small cell lung cancer by systems pharmacology method (Liu et al., 2018). However, previous systems pharmacology studies usually consider drug-like compounds in herb databases. In contrast, whether the compounds can be absorbed into the blood is often neglected, which may lead to the results that the active ingredients and predicted targets deviate from the truth. The serum pharmacochemistry method could help to discover the compounds absorbed into blood of the Chinese medicinal formula as the clues of active ingredients and is widely used to reveal the efficacy of TCMs (Yan et al., 2017). Therefore, the detected constituents absorbed into blood can provide the basis of chemical composition for further systems pharmacology investigation.

**Figure 1:**
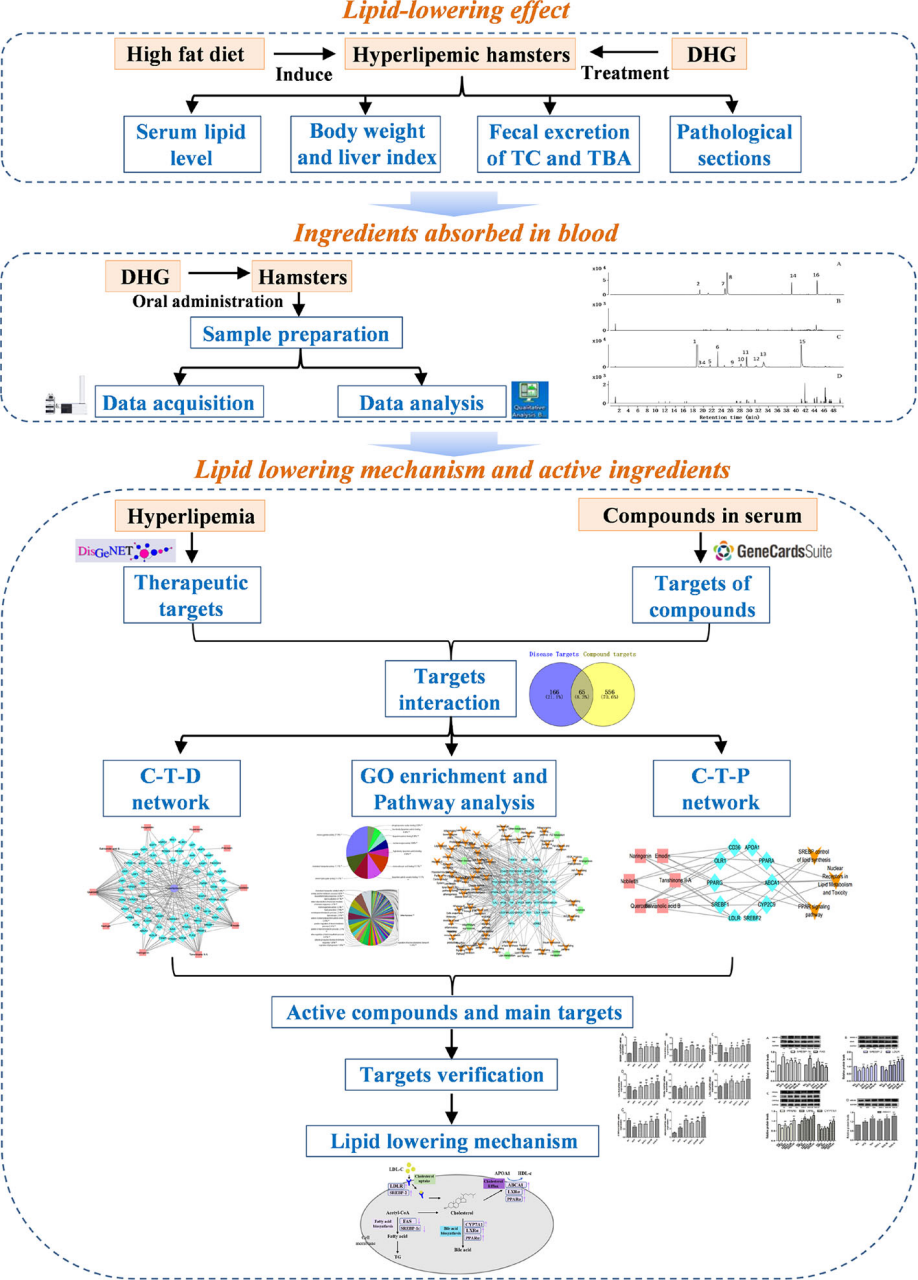
The scheme of investigating the lipid-lowering mechanism and active ingredients of DHG based on systems pharmacology. DHG, Danhe granule.

In this work, a systems pharmacology approach was employed to investigate the lipid-lowering mechanism and active components of DHG. The detailed flowchart is shown in **Figure 1**. First, the high-fat diet (HFD)-induced hyperlipidemic hamster model was used to evaluate the hypolipidemic effect of DHG. Then, a UPLC-Q-TOF/MS method was performed to identify the constituents absorbed into blood after DHG administration. On this basis, target prediction, network mapping, and pathway analysis were carried out to systematically explore the underlying reciprocity between active compounds, active targets and pathways. Finally, some crucial targets were experimentally validated. This work will serve to deepen the understanding of the effective substances and mechanisms of DHG in the treatment of hyperlipidemia and contribute to its development and application.

## Materials and Methods

### Chemicals and Materials

Simvastatin (Hangzhou MSD Pharmaceutical Co., Ltd., China) was taken as a positive control. The HFD contained 10% lard, 10% egg yolk powder, 0.3% cholesterol, and 79.7% standard diet was provided by Beijing HFK Bioscience Co., Ltd., China. Reference standards (purity ≥ 98%) of gallic acid (M-017-161223), polydatin (M-017-161223), isoquercetin (Y-076-161216), hesperidin (110721-201818), salvianolic acid B (D-012-170417), emodin 8-*O*-*β*-D-glucoside (D-018-160928), hyperin (J-012-170317), nuciferine (111566-201706), quercetin (H-009-170426), nobiletin (C-015-170316), emodin (Must-16031601), and tanshinone II A (D-008-170508) were purchased from Chengdu Herbpurify Co., Ltd. (China). High-performance liquid chromatography (HPLC)-grade acetonitrile, methanol, and formic acid were supplied by Merck (Darmstadt, Germany). Ultrapure water was obtained using a Milli-Q water purification system from Millipore (Bedford, MA, USA). TC, TG, LDL-c, and HDL-c assay kits were purchased from Nanjing Jiancheng Bioengineering Institute (Nanjing, China). BCA protein assay kit was purchased from Beyotime (Shanghai, China). total bile acid (TBA) assay kit was provided by Crystal Chem (USA). Anti-SREBP-1c, anti-liver X receptor alpha (LXRα), anti-cholesterol 7α-hydroxylase (CYP7A1), and anti-ATP binding cassette subfamily A member 1 (ABCA1) were purchased from ImmunoWay Biotechnology Company (Newark, DE, USA). Anti-SREBP-2, anti-low-density lipoprotein receptor (LDLR), anti-fatty acid synthase (FAS), anti-peroxisome proliferation-activated receptor (PPAR)α, and anti-GAPDH were obtained from Proteintech Group Inc. (Chicago, USA). Power sybr green pcr mix and Revert aid first strand cDNA synthesis kit were purchased from Thermo Fisher Scientific Inc. (St. Louis, MO, USA). The primer was provided by Sangon Biotech Co., Ltd. (Shanghai, China).

### DHG Preparation and HPLC Analysis

DHG (batch No. D1905007) was provided by the hospital preparation room, China–Japan Friendship Hospital (Beijing, China). Saliae Mil Tiorrhizae Radix et Rhizoma (Lot No.80270602) (the root and rhizome of *Salvia miltiorrhiza* Bunge a perennial herbal plant of the *Salvia* L. genus, the *Labiatae* family), Polygoni Cuspidati Rhizoma et Radix (Lot No.80510801) (the root and rhizome of *Reynoutria japonica* Houtt., genus *Reynoutria* Houtt., family *Polygonaceae*), Crataegi Fructus (Lot No.82230701) (the fruit of *Crataegus pinnatifida* Bunge the short arbor plants of the *Crataegus* L. genus, the *Rosaceae* family), Citri Reticulatae Pericarpium (Lot No. 81450601) (the peel of *Citrus × aurantium* L., genus *Citrus* L., family *Rutaceae*), Coicis Semen (Lot No. 70511101) (the mature kernel of *Coix lacryma-jobi var. ma-yuen* (Rom.Caill.) Stapf a perennial herbal plant of the *Coix* L. genus, the *Poaceae* family) and Nelumbinis Folium (Lot No. 83720701) (the leaf of *Nelumbo nucifera* Gaertn., genus *Nelumbo* Adans., family *Nelumbonaceae*) were obtained from Beijing San He Co., Ltd. (Beijing, China) and identified by Professor Xueyong Wang (School of Chinese Materia Medica, Beijing University of Chinese Medicine. Voucher specimens including Saliae Mil Tiorrhizae Radix et Rhizoma (No. CMAT-SM-201806), Polygoni Cuspidati Rhizoma et Radix (No. CMAT-PC-201809), Crataegi Fructus (No. CMAT-CP-201810), Citri Reticulatae Pericarpium (No. CMAT-CR-201811), Coicis Semen (No. CMAT-CL-201808), and Nelumbinis Folium. (No. CMAT-NN-201807) were deposited in School of Chinese Materia Medica, Beijing University of Chinese Medicine (Herbarium Code: BCMM). Besides, detailed drug materials information, including herbal material origin and the scan of the vouchers, was given in **Supplementary Table S1**. A mixture of Saliae Mil Tiorrhizae Radix et Rhizoma (1000g), Coicis Semen (3000g), Polygoni Cuspidati Rhizoma et Radix (1000g), Crataegi Fructus (1000g), Citri Reticulatae Pericarpium (1000g), and Nelumbinis Folium (1500g) at a weight ratio of 1:3:1:1:1:1.5 were extracted three times by refluxing with 12-fold of water (volume/weight) for 1 h each time. The liquid extract was filtered and concentrated to a density of 1.08 (60°C). 95% ethanol was added into the concentrated liquid to 60% ethanol concentration, and then it was precipitated at room temperature for 24 h. The supernatant was condensed and dried to yield 1060 g powder using the decompression drying method at 60°C.

The HPLC analysis of DHG was performed on Agilent 1260 Infinity HPLC system (Agilent Technologies, Waldbronn, Germany) with a ZORBAX Eclipse XDB-C18 column (4.6 × 250 mm, 5 μm) at a flow rate of 1.0 ml/min and a 30°C column temperature. The mobile phase was composed of solvent A (water-0.1% phosphoric acid) and solvent B (acetonitrile): 0–55 min, 5%–30% B; 55–60 min, 30%%95% B; 70–75 min, maintained at 95% B. The injection volume was 10 μl and the UV detection wavelength was set at 286 nm.

### Animals and Experimental Design

Healthy 4-week-old male Syrian golden hamsters weighing 90 ± 3 g were obtained from Beijing Vital River Laboratory Animal Technology Co., Ltd [SCXK(JING) 2016-0011]. All animals were housed in stainless steel wire-mesh cages individually in a room kept at 22 ± 2°C with 50%–60% relative humidity and a 12-h light/dark cycle and allowed free access to normal food and water. After acclimatization with the facility for a week, animals were randomly assigned to different dietary groups: the normal diet control group (NC) (n = 10) and the experimental group (n = 50). The experimental group was fed with a HFD, which was composed of 10% lard, 10% egg yolk powder, 0.3% cholesterol, and 79.7% standard diet. Animals had free access to both food and distilled water, which were provided fresh every day. After 2 weeks, dyslipidemia hamsters were determined according to the lipid levels including serum TC, TG, LDL-c, and HDL-c and divided into the HFD group (n = 10), three DHG-treated groups with a dose of 0.37 (1/3-fold clinical doses) g/kg/day (n = 10), 1.10 (clinical doses) g/kg/day (n = 10), and 3.3 (3-fold clinical doses) g/kg/day (n = 10), and 2.4 mg/kg/day of simvastatin (Sim) was used as positive control (n = 10). In subsequent an 8-week experiment, except for the NC group (n = 10), the remaining five groups were fed with the HFD. During the experiment, food intake was measured daily. Body weight was recorded every seven days. Feces of each hamster were collected third day prior to termination of the study for the analysis of cholesterol and bile acid.

This study was conducted in conformity with the policies and procedures of the Ethics Committee of Beijing University of Traditional Chinese Medicine (permit number: BUCM-4-2018091101-3048). At the end of the experiment, blood from each hamster was collected *via* puncturing the retro-orbital sinus with a capillary tube after overnight fasting. After collecting the blood, the adipose tissue, liver, heart, spleen, lung, kidney, and brain were removed, rinsed with a physiological saline solution, and weighed. The viscera index (%) = viscera weight (g)/body weight (g) ×100. Then the liver and adipose tissue were stored at −80°C.

### Biochemical Parameters

The serum sample was prepared by centrifugation of blood at 1000×g for 10 min at 4°C and stored at −80°C until analysis. Serum TC, TG, LDL-c, and HDL-c were measured by enzymatic colorimetric methods using commercial kits (Nanjing Jiancheng Bioengineering Institute, Nanjing, China). The total fat in feces was extracted with chloroform/methanol (2:1, v/v) according to the reported method (Folch et al., 1957). After lipid extraction, the TC concentration was assayed by commercial kits (Nanjing Jiancheng Bioengineering Institute, Nanjing, China). The TBA in feces was extracted by 75% ethanol at 50°C for 2 h (Ning et al., 2015), then the level of TBA was analyzed by commercial kits (Crystal Chem, USA). The atherogenic index (AI) was calculated by the equation AI = (TC −HDL-c)/HDL-c (Guo et al., 2011).

### Histological Analysis

For histological analysis of lipid accumulation in the liver, tissues were embedded in Tissue-TekOCT cryostat molds and frozen at −80°C. After the sections were obtained from the liver tissues above, the liver tissue sections were stained with 0.5% Oil Red O. The epididymal adipose tissues were fixed in 10% formalin, dehydrated, embedded in paraffin, and sectioned at 5 μm on to poly-L-lysine-coated slides. Then the sections were stained with hematoxylin and eosin, photographed with an upright fluorescence microscope (Nikon Instruments CO., Ltd, Japan).

### Serum Ingredients Analysis After DHG Taken Orally

#### Sample Preparation

Six male Syrian golden hamsters (weighing from 95 to 110 g) were randomly divided into the experiment group (n = 4) and blank group (n = 2). The DHG was administrated by oral administration at a single dose of 3.30 (3-fold clinical doses) g/kg body weight to the experiment group. At the same time, an equivalent of distilled water was given to the blank group. For the collection of serum samples, the hamsters were anesthetized by intraperitoneal injection of 10% aqueous chloral hydrate after oral administration of the DHG. The blood samples were collected from the hepatic portal at 0.5, 1, 2, and 4 h, respectively. The collected samples were centrifuged at 4000 rpm for 15 min at 4°C and then mixed to obtain a pooled serum. The blank serum was collected in the same way. A volume of 2 ml serum samples was immediately treated with 6 ml acetonitrile to precipitate serum proteins. After centrifuged at 12,000 rpm for 15 min at 4°C, the supernatants were dried under a vacuum concentrator and dissolved in 200 μl methanol for LC-MS analysis.

#### Analysis Method

The sample detection was performed by an Agilent 1290/6550 iFunnel Q-TOF MS system with both negative and positive ionization modes. Samples were separated by the Agilent Eclipse C18 RRHD (2.1 mm × 150 mm, 1.8 μm), the temperature was maintained at 40°C, and the injection volume was 2 μl. The mobile phase consisted of water–0.1% formic acid (A), and acetonitrile–0.1% formic acid (B) (5% B at 0–5 min, 5%–20% B at 5–15 min, 20%–35% B at 15–35 min, 35%–95% B at 35–45 min, 95%–100% B at 45–50 min) and the flow rate was 0.4 ml/min. The Dual AJS ESI source conditions were as follows: gas temperature, 200°C; gas flow, 14 L/min; nebulizer pressure, 45 psi; sheath gas temperature, 300°C; sheath gas flow, 12 L/min; capillary voltage, 3500 V (−)/4000 V (+); nozzle voltage, 1000 V; fragmentor voltage, 380 V; MS range, 100–1400 *m*/*z*. The sample collision energy was set at 10, 20, and 40 V. The mass spectral data were processed by Agilent Mass Hunter Qualitative Analysis B.07.00 software (version B.07.00, Agilent Technologies, USA).

### Network Construction

The network construction mainly includes the following three steps: (1) Disease target collection. DisGeNET is a discovery platform containing one of the largest collections of genes associated with human diseases, which was typically used for the discovery of information about disease-related targets (Zhu et al., 2018). After screening targets with relevance score ≥ 0.001 from DisGeNET database (http://www.disgenet.org/), the known therapeutic targets of treatment for hyperlipidemia were obtained. (2) DHG candidate targets collection. Genecards is an integrative human genes database, which could be employed to search for target information related to compounds (Chen et al., 2019). The candidate targets of DHG (relevance score ≥1) were collected from Genecards Databases (http://www.genecards.org/) based on the 16 compounds identified in hamster serum. After retaining the common targets for disease and serum compounds of DHG, removing duplicate targets, and the compounds without targets, then the potential targets and active compounds were obtained for next network mapping. (3) The network construction. The active compounds–potential targets–disease network (C–T–D network) was established by mapping the potential targets with the active compounds and hyperlipidemia, made by Cytoscape (Version 3.6.0) (http://www.cytoscape.org/). The potential targets, which could be associated with other targets by the String database (https://string-db.org/), were used to conduct further gene ontology (GO) enrichment analysis and pathway analysis.

### GO Enrichment and Pathway Analysis

GO enrichment analysis with biological process and molecular function of potential targets were carried out for biological function annotation based on a Cytoscape (Version 3.6.0) plugin Clue GO (Version 2.5.5) with the criterion of *p*-value ≤ 0.01 and kappa score 0.7. In addition, the DAVID database (https://david.ncifcrf.gov/home.jsp, Version 6.8) was utilized to analyze the representative pathways of the potential targets, and a *p*-value ≤ 0.05 significant level was used. Meanwhile, the targets–pathways (T–P) network was conducted by Cytoscape (Version 3.6.0) to visualize the relationship of the potential targets and related pathways. Based on the pathway analysis, we focused on the three pathways associated with lipid metabolism. Then the compounds–targets–pathways (C–T–P) network was further mapped to investigate the relationship between compounds, targets, and pathways using Cytoscape (Version 3.6.0) similarly.

### RNA Extract, cDNA Synthesis, and Real-time PCR

Total RNA was prepared from livers using TRIzol reagent. cDNA was synthesized using Revertaid first strand cDNA synthesis kit. The quantitative real-time polymerase chain reaction (RT-PCR) reaction, containing target genes and SYBR Green PCR master mix, was performed on Bio-Rad CFX connect real-time system (Bio-Rad, USA). The sequences of the primers were listed in **Supplementary Table S2**. The PCR conditions were as follows: 95°C for 3 min, cycled at 95°C for 10 s, 55°C for 10 s, and 72°C for 30 s for 40 cycles, for melt curve from 55°C to 95°C with an interval at 0.5°C for 5 s. Each experiment was performed in triplicate. The relative levels of each gene expression were determined by the 2^−ΔΔCt^ method with GAPDH as an internal control to normalize all the mRNA levels.

### Western Blot Analysis

The liver tissue was homogenized in sodium chloride–Tris–EDTA buffer (250 mM sucrose, 10 mM Tris/HCl, 1 mM EDTA/Na, pH 8.0) with protease inhibitor. Total protein samples were separated by SDS-PAGE at 120 V for 1.5 h at room temperature and then electrotransferred onto PVDF membranes at 200 mA for 2.5 h at 4°C. To block non-specific binding site, the membranes were blocked with 5% non-fat dry milk in TBST for 2 h at room temperature, followed by incubated with primary antibodies overnight at 4°C including anti-rabbit SREBP-1c (1:1000, Immunoway), anti-rabbit SREBP-2 (1:1000, Proteintech), anti-rabbit LDLR (1:1000, Proteintech), anti-rabbit FAS (1:1000, Proteintech), anti-rabbit LXRα (1:1000, Immunoway), anti-rabbit CYP7A1 (1:1000, Immunoway), anti-rabbit PPARα (1:750, Proteintech), anti-rabbit ABCA1 (1:1000, Immunoway), and anti-rabbit GAPDH (1:1000, Proteintech). Afterward, the membranes were incubated with HRP-linked anti-IgG (1:2000, Proteintech) for 2 h at room temperature. Densitometric measurement of chemiluminescent bands was performed using Image Lab™ software on ChemiDoc XRS+ (Bio-Rad, USA).

### Statistical Analysis

Results were expressed as mean ± standard deviation (SD). Statistical analysis was performed with one-way analysis of variance (ANOVA) using the SPSS 19.0 program, and the differences in mean values among groups were assessed using Duncan’s multiple range test. *p* < 0.05 was considered as statistically significant difference, and *p* < 0.01 was extremely significant difference.

## Results

### HPLC Profile of DHG

**Figure 2:**
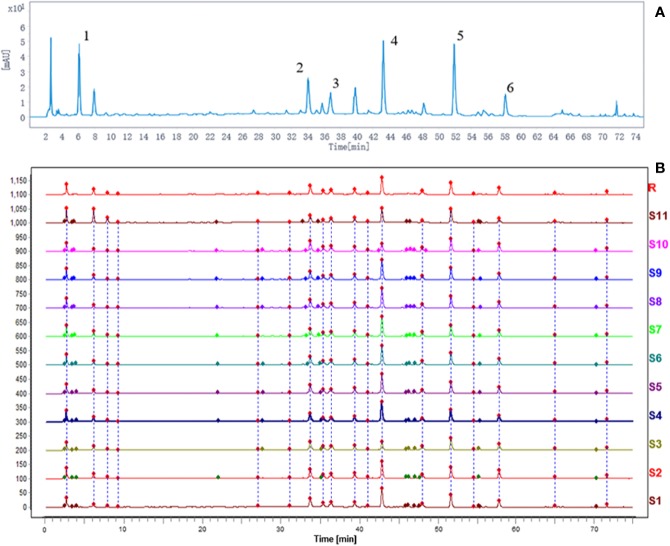
HPLC fingerprint analysis of DHG. The HPLC chromatographic of DHG **(A)**. Comparability results of reproducibility of DHG samples **(B)**. The main components of DHG in the chromatograms are as follows: (1) gallic acid, (2) polydatin, (3) isoquercetin, (4) hesperidin, (5) salvianolic acid B, and (6) emodin-8-O-β-D-glucoside. HPLC, high-performance liquid chromatography.

Using the current HPLC method, the reproducibility analysis of the 11 batches of DHG samples was conducted (**Figure 2B**), and the results (similarities > 0.94) indicated that the preparation process of DHG was reasonable and feasible (**Supplementary Information** for details). Besides, to determine the main chemical composition of DHG, an HPLC analysis was performed and the chromatogram was shown in **Figure 2A**. Six compounds from the HPLC chromatographic peaks, including gallic acid, polydatin, isoquercetin, hesperidin, salvianolic acid B, and emodin-8-O-β-D-glucoside, were identified by comparing with standard references. The contents of these six compounds in the DHG sample (batch No. D1905007) were determined as 7.09, 4.37, 5.39, 9.62, 13.09, and 2.15 mg/g, respectively. The chromatograms of mixed standards and their structures were shown in **Supplementary Figure S1**.

### Protective Effects of DHG on Hyperlipidemia

#### DHG Modulated Serum Lipid Levels

**Figure 3:**
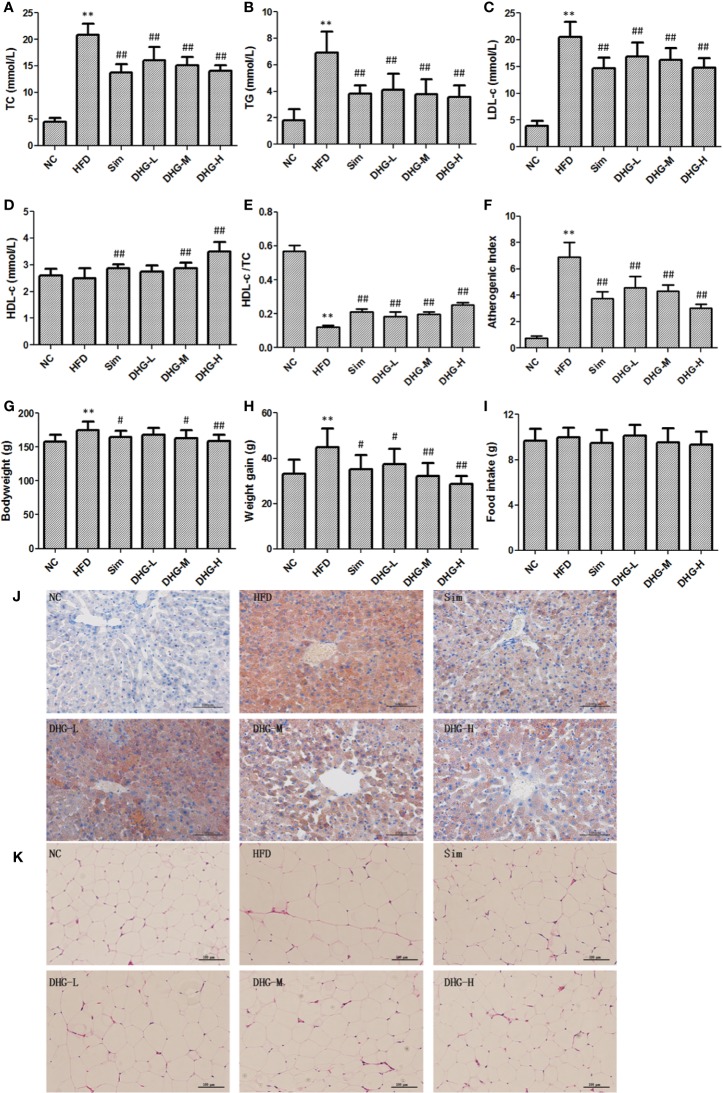
Protective effect of DHG on hyperlipidemic hamsters induced by HFD. Values were mean ± SD, n =10. **(A)** Serum TC levels. **(B)** Serum TG levels. **(C)** Serum LDL-c levels. **(D)** Serum HDL-c levels. **(E)** HDL-c/TC ratio. **(F)** Atherosclerosis index. **(G)** Body weight in the 10th week. **(H)** Body weight gain of each group. **(I)** The food intake of each group. **(J)** Histological analysis of liver. Liver sections were stained with Oil Red O, Scale bar = 100 μm. **(K)** Histological analysis of epididymal adipose tissue. Epididymal adipose tissue sections were stained with hematoxylin and eosin, Scale bar = 100 μm. NC, normal control group; HFD, high-fat diet group; Sim, simvastatin (2.40 mg/kg/day); DHG-L, DHG at low dosage (0.37 g/kg/day); DHG-M, DHG at medium dosage (1.10 g/kg/day); DHG-H, DHG at high dosage (3.30 g/kg/day). TC, total cholesterol; TG triglyceride; LDL-c, low-density lipoprotein cholesterol; HDL-c, high-density lipoprotein cholesterol. ^**^p < 0.01 vs NC; ^#^*p* < 0.05 vs HFD; ^##^*p* < 0.01 vs HFD.

Firstly, we investigate the effect of DHG on serum lipid levels in hyperlipidemic hamsters. As shown in **Figures 3A–C**, the results show that the elevated serum levels of TC, TG, and LDL-c in dyslipidemia hamsters were significantly declined after treatment with DHG (*p* < 0.01). Notably, DHG-H therapy decreased the serum TC, TG, and LDL-c level by 32.41%, 48.56%, and 28.23%, respectively, and enhanced the serum HDL-c level by 40.96% in hyperlipidemic hamsters (**Figure 3D**). From the data, DHG-H showed a similar effect as Sim in reducing serum TC, TG, and LDL-C levels, while a strong effect than Sim in improving serum HDL-c level. As seen in **Figures 3E, F**, HFD administration resulted in a significant increase in the AI (*p* < 0.01) and a decrease in serum HDL-c/TC ratio (*p* < 0.01) in hamster compared with the NC group. After 8 weeks of treatment, the serum HDL-c/TC ratio was significantly enhanced by DHG treatment compared to the HFD group (*p* < 0.01). Besides, the phenomenon was associated with a 34.01%, 37.77%, and 56.01% reduction in AI in DHG at low dosage (DHG-L), DHG at medium dosage (DHG-M), and DHG at high dosage (DHG-H) treatment group (*p* < 0.01). In addition, we also investigated the effect of DHG on the fecal excretion of TC and TBA in hamsters. As shown in **Table 1**, the fecal excretion of TC and TBA in hamsters was increased by HFD compared with the NC group (*p* < 0.05). DHG-H group exhibited a higher fecal TBA level compared with the HFD group (*p* < 0.01). Besides, the fecal TBA level was significantly enhanced by 21.67% and 32.51% with DHG-M and DHG-H treatment compared to the HFD group (*p* < 0.05), respectively. These data suggested that DHG has an excellent effect on improving blood lipid levels.

**Table 1:** Effect of DHG on fecal excretion of TC and TBA levels in hamsters.

Group	TC (μmol/g)	TBA (μmol/g)
NC	3.05 ± 0.37	1.61 ± 0.28
HFD	5.45 ± 0.88^**^	2.03 ± 0.43^*^
Sim	6.18 ± 0.85	2.10 ± 0.35
DHG-L	6.34 ± 1.17	2.25 ± 0.54
DHG-M	6.65 ± 1.89	2.47 ± 0.42^#^
DHG-H	7.48 ± 0.96^##^	2.69 ± 0.21^##^

Values were mean ± SD, n = 10. TC, total cholesterol; TBA, total bile acids; NC, normal control group; HFD, high-fat diet group; Sim, simvastatin (2.40 mg/kg/day); DHG-L, DHG at low dosage (0.37 g/kg/day); DHG-M, DHG at medium dosage (1.10 g/kg/day); DHG-H, DHG at high dosage (3.3 g/kg/day). ^*^p < 0.05, ^**^p < 0.01 vs NC group; ^#^p < 0.05, ^##^p < 0.01 vs HFD group.

#### DHG Reduced Body Weight Gain, Liver Index, and Fat Weight

Since hyperlipidemia often accompanied by body weight gain as well as fat increase and liver index change, we studied the effect of DHG on these aspects. The body weight of each group from the 10th week was recorded in **Figure 3G** and the weight gain of each group was shown in **Figure 3H**. The results indicated that the intake of the HFD diet induced a remarkable increase in the body weight and body weight gain of hamsters compared with the NC group (*p* < 0.01). Compared with the HFD group, DHG administration notably lowered the body weight in hyperlipidemic hamsters. Besides, DHG treatment significantly decreased body weight gain in hyperlipidemic hamsters by 16.60%, 28.01%, and 36.04%, respectively, at three dose levels. During the experimental period, there were no significant changes in daily food intake among different groups (**Figure 3I**), which indicated the changes in body weight in drug therapy groups were might mainly result from DHG treatment. As shown in **Table 2**, HFD led to a significant increase in the perirenal adipose tissue weight, epididymal adipose tissue weight and liver index compared with the NC group (*p* < 0.01). After treatment, DHG-M and DHG-H groups showed a significant decreasing trend in the liver index, the perirenal and epididymal adipose tissue weight in hyperlipidemic hamsters. Notably, DHG-H lowered perirenal adipose tissue weight, epididymal adipose tissue weight and liver index by 36.09%, 19.24%, and 15.19%, respectively. Besides, DHG and Sim treatments have little impact on other viscera indexes such as heart index, spleen index and lung index in hyperlipidemic hamsters (**Table 2**). These data indicated the beneficial effects of DHG on body weight, fat weight, and liver index in hyperlipidemic hamsters.

**Table 2:** Effect of DHG on perirenal and epididymal adipose tissue weight and relative viscera index of hyperlipidemic hamsters.

Group	NC	HFD	Sim	DHG-L	DHG-M	DHG-H
Perirenal adipose tissue (g)	2.29 ± 0.34	3.38 ± 0.50^**^	2.35 ± 0.26^##^	2.60 ± 0.37^##^	2.35 ± 0.51^##^	2.16 ± 0.37^##^
Epididymal adipose tissue (g)	4.28 ± 0.31	5.25 ± 0.62^**^	4.42 ± 0.53^##^	4.67 ± 0.52^#^	4.41 ± 0.85^##^	4.24 ± 0.52^##^
Liver index (%)	3.33 ± 0.31	5.53 ± 0.29^**^	4.58 ± 0.51^##^	5.35 ± 0.40	5.12 ± 0.38^#^	4.69 ± 0.32^##^
Heart index (%)	0.32 ± 0.02	0.32 ± 0.03	0.33 ± 0.02	0.32 ± 0.03	0.33 ± 0.03	0.33 ± 0.02
Spleen index (%)	0.07 ± 0.02	0.07 ± 0.01	0.08 ± 0.02	0.07 ± 0.01	0.07 ± 0.01	0.07 ± 0.02
Lung index (%)	0.39 ± 0.04	0.38 ± 0.02	0.41 ± 0.03	0.40 ± 0.02	0.40 ± 0.03	0.41 ± 0.03
Kidney index (%)	0.71 ± 0.05	0.68 ± 0.04	0.69 ± 0.07	0.67 ± 0.04	0.69 ± 0.05	0.68 ± 0.04
Brain index (%)	0.53 ± 0.03	0.51 ± 0.04	0.53 ± 0.03	0.52 ± 0.03	0.52 ± 0.05	0.54 ± 0.03

Values were mean ± SD, n = 10. NC, normal control group; HFD, high-fat diet group; Sim, simvastatin (2.40 mg/kg/day); DHG-L, DHG at low dosage (0.37 g/kg/day); DHG-M, DHG at medium dosage (1.10 g/kg/day); DHG-H, DHG at high dosage (3.3 g/kg/day). ^**^p < 0.01 vs NC; ^#^p < 0.05 vs HFD; ^##^p < 0.01 vs HFD.

#### Histopathological Changes in Liver and Adipose Tissues

To further evaluate the effects of DHG on the pathological changes of liver and adipose tissue, the Oil red O staining liver tissue and hematoxylin & eosin staining epididymal fat tissue were employed. As shown in **Figure 3J**, DHG substantially decreased the amount of fat accumulated in hyperlipidemic hamster liver. Moreover, histological analysis revealed that the Sim group and DHG-H group with fewer lipid droplets showed a more potent effect in inhibiting hepatic fat accumulation. In the hematoxylin and eosin staining of epididymal adipose tissue sections (**Figure 3K**), it’s easy to see that the adipocyte size in the HFD group was larger than the NC group. Treatment with Sim and DHG-H suppressed enlargement of the adipocytes in epididymal adipose tissue in hyperlipidemic hamsters.

### The Ingredients Absorbed Into Blood After Oral Administration of DHG

The ultra-high performance liquid chromatography coupled with quadrupole time-of-flight mass spectrometry (UHPLC-Q-TOF/MS) was employed to analyze the main ingredients absorbed into the blood after oral administration of DHG. According to the accurate mass measurements, reference standards, fragmentation behavior, and related literatures, a total of 16 prototype compounds in hamster serum from DHG were first identified, including polydatin, n-nornuciferine, hyperin, isoquercitrin, naringin, hesperidin, o-nornuciferine, nuciferine, quercetin, emodin 8-O-β-D-glucoside, naringenin, torachryson, nobiletin, emodin, and tanshinone II-A. The detailed information of the 16 compounds was summarized in **Table 3**. The detailed identification process can be seen in the **Supplementary Material**. The extracted ion chromatograms of the 16 compounds in dosed groups and blank groups in positive and negative ion modes were shown in **Supplementary Figure S2**, and the total ion chromatograms of DHG sample were shown in **Supplementary Figure S3**.

**Table 3:** The compounds identified in hamster serum after oral administration of DHG.

No.	Rt (min)	Observed *m*/*z*	Error (ppm)	Formula	MS/MS fragments	Name
1	18.75	389.1240 [M − H]^−^	0.81	C_20_H_22_O_8_	227.0722, 185.0616, 143.0508	Polydatin^a^
2	19.26	282.1495 [M + H]^+^	−2.02	C_18_H_19_NO_2_	251.1093, 236.0849, 219.0828, 208.0897, 191.0875, 179.0867, 165.0709	N-nornuciferine
3	19.61	463.088 [M − H]^−^	0.75	C_21_H_20_O_12_	300.0281, 271.0255, 255.0305, 243.0305, 178.9994, 151.0043	Hyperin^a^
4	20.01	463.0874 [M − H]^−^	1.79	C_21_H_20_O_12_	300.0282, 271.0255, 255.0305, 243.0307, 227.0355, 151.0044	Isoquercitrin
5	21.46	579.1719 [M − H]^−^	0.01	C_27_H_32_O_14_	271.0619, 227.0722, 175.0044, 151.0044, 119.0510, 107.0148, 93.0353	Naringin
6	23.1	609.1833 [M − H]^−^	−1.28	C_28_H_34_O_15_	325.0718, 301.0722, 286.0485, 257.0823, 242.0590, 164.0122, 151.0042, 125.0250	Hesperidin^a^
7	24.67	282.1493 [M + H]^+^	−1.45	C_18_H_19_NO_2_	265.1245, 250.1006, 235.0772, 219.0815, 207.0816, 191.0862, 179.0859	O-nornuciferine
8	25.13	296.1642 [M + H]^+^	1.23	C_19_H_21_NO_2_	265.1246, 250.1008, 235.0773, 219.0819, 207.0821, 191.0869, 179.0866	Nuciferine^a^
9	26.25	717.1463 [M − H]^−^	−0.59	C_36_H_30_O_16_	519.0933, 339.0510, 321.0406, 295.0613, 185.0248, 109.0299	Salvianolic acid B^a^
10	28.06	301.0358 [M − H]^−^	−1.2	C_15_H_10_O_7_	273.0394, 245.0440, 178.9979, 151.0028, 121.0284, 107.0145	Quercetin^a^
11	29.29	431.0992 [M − H]^−^	−1.57	C_21_H_20_O_10_	269.0458, 240.0436, 225.0564, 210.0320, 197.0604, 181.0666	Emodin 8-O-β-D-glucoside^a^
12	31.12	271.0616 [M − H]^−^	−1.69	C_15_H_12_O_5_	151.0042, 119.0508, 107.0144	Naringenin
13	32.92	245.0824 [M − H]^−^	−1.95	C_14_H_14_O_4_	230.0594, 215.0362, 187.0396, 159.0458	Torachryson
14	38.98	403.1395 [M + H]^+^	−1.73	C_21_H_22_O_8_	388.1115, 373.0918, 359.1090, 327.0858, 211.0253, 165.0555	Nobiletin^a^
15	40.97	269.0461 [M − H]^−^	−1.88	C_15_H_10_O_5_	241.0514, 225.0565, 197.0615	Emodin^a^
16	44.21	295.1327 [M + H]^+^	−0.08	C_19_H_18_O_3_	277.1235, 266.0922, 262.0991, 252.0794, 249.1276, 235.0755, 206.1095, 191.0855, 179.0870	Tanshinone II A^a^

a Compared with authentic compounds.

### Network Construction and Enrichment Analysis

#### C–T–D Network

**Figure 4:**
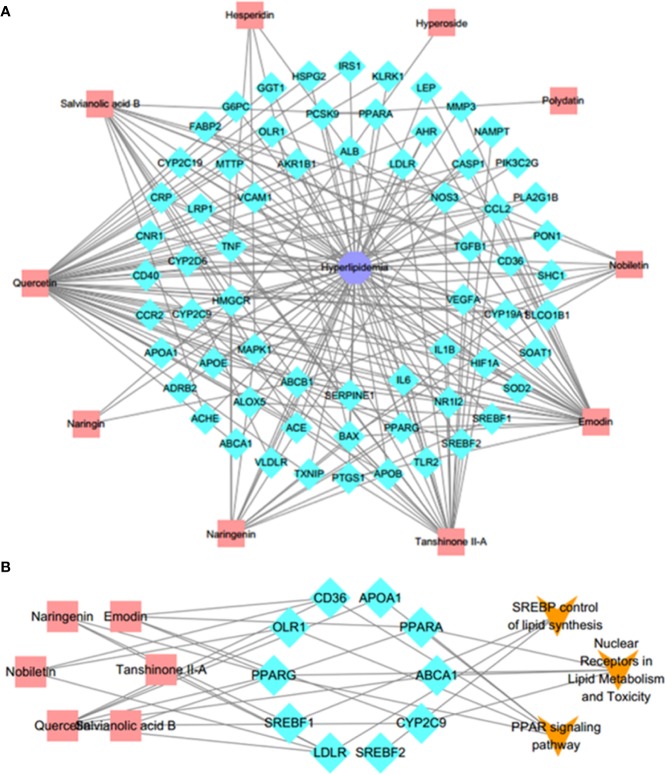
C–T–D network and C–T–P network. **(A)** Active C–T–D network of 10 compounds and 65 common potential targets in treatment of hyperlipemia. The aquamarine rhombus nodes represent the targets, red rectangle nodes delineate the compounds, and indigo octagon nodes delineate the disease. **(B)** C–T–P network. The C–T–P network was constructed by linking compounds, targets, and their related pathways. The nodes represent compounds (red rectangle), targets (aquamarine rhombus), and pathways (orange v-shaped). C–T–D, compounds–potential targets–disease; C–T–P, compounds–targets–pathways.

To intuitively reflect the relationship between compounds, targets and disease, Cytoscape 3.6.0 was employed to map out a C–T–D network diagram. Firstly, based on the Genecards Databases (http://www.genecards.org/), 621 targets of compounds absorbed into the blood were predicted after filtering out the overlapping targets and targets with relevance score < 0.001. The target information of compounds is shown in **Supplementary Table S3**. Secondly, after consulting with the DisGeNET database (http://www.disgenet.org/), a total of 231 candidate targets of hyperlipemia with relevance score ≥1 were obtained (**Supplementary Table S4**). Finally, the 65 potential targets, which associated with both disease and serum ingredients, were screened out to generate a C–T–D network diagram to reflect the relationship between components, targets, and disease (**Supplementary Table S5**). As shown in **Figure 4A**, there were 10 components, 65 potential targets, and 1 disease contained in the C–T–D network. These 10 compounds were quercetin, emodin, polydatin, salvianolic acid B, tanshinone II-A, hesperidin, hyperoside, naringenin, naringin, and nobiletin. These compounds are mainly categorized as flavonoids, phenolic acids, anthraquinones, tanshinone, and stilbene glycosides. The 8 ones including quercetin, emodin, salvianolic acid B, tanshinone II-A, hesperidin, naringenin, naringin, and nobiletin of 10 compounds have high correlation degree (degree ≥ 3), which may play a significant role in the network. For instance, quercetin is a considerable compound of DHG with the highest number of target interaction (degree = 46). Previous studies have demonstrated that quercetin could reduce fat accumulation in the liver by regulating lipid metabolism genes (Jung et al., 2013). The LDLR (degree = 5) was targeted by four compounds from DHG, which played an important role in hepatic cholesterol metabolism (Li et al., 2018). Meanwhile, some active compounds were also linked to multiple targets, suggesting potential synergies between them. In summary, we first mapped the C–T–D network focusing on the ingredients absorbed into the blood to preliminary explain the effect of DHG on hyperlipidemia and screened 10 components and 65 potential targets.

#### GO Enrichment Analysis

Firstly, the protein–protein interaction (PPI) network of the 65 common targets was built by String database (https://string-db.org/) based on the STRING score values (with the highest confidence 0.9), and the targets with no connection with any others were neglected in our following study. Then, to further investigate the 48 remained potential targets, GO enrichment analysis, including molecular function and biological processes, was performed by ClueGO (version 2.5.5). For molecular functions analysis (**Supplementary Figure S4A**), the 10 significant GO terms associated with hyperlipidemia were enriched, such as cholesterol transporter activity, steroid hydroxylase activity, lipoprotein particle receptor binding, high-density lipoprotein particle binding, lipoprotein particle receptor binding, and low-density lipoprotein particle binding, etc. Moreover, as shown in **Supplementary Figure S4B**, the GO enrichment results revealed that these potential targets were involved in many biological processes, including cholesterol transporter activity, secondary alcohol metabolic process, lipid localization, lipid storage, regulation of plasma lipoprotein particle levels, positive regulation of steroid metabolic process, etc. The results indicated that these targets were associated with the pathogenesis of hyperlipidemia and related diseases. At the same time, many molecular functions and biological processes, such as cholesterol transporter activity, lipoprotein particle receptor binding and lipid localization, were closely related to lipid metabolism, which suggested that lipid metabolism may play an essential role in the pathogenesis or treatment mechanism of hyperlipidemia, and we will focus on it.

#### C–T–P Network

**Figure 5:**
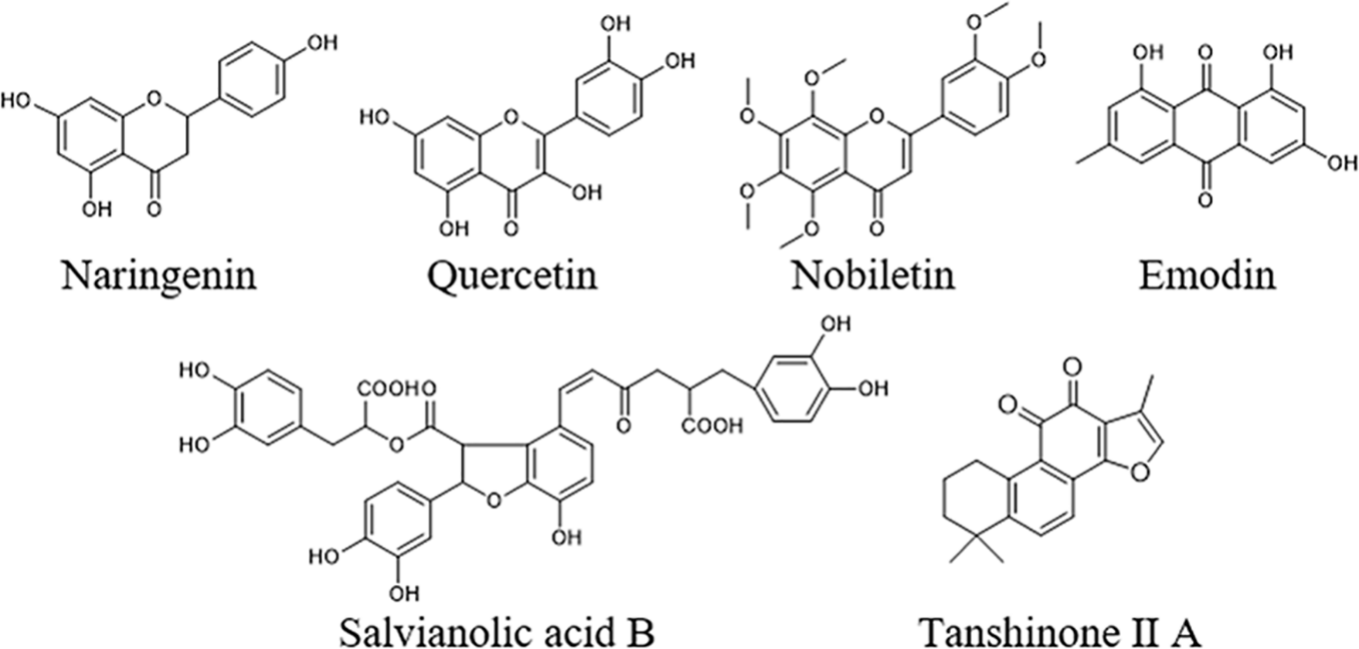
Chemical structures of the six active compounds in DHG including naringenin, nobiletin quercetin, emodin, salvianolic acid B, and tanshinone II A.

To further elucidate the underlying curative mechanisms of DHG for the treatment of hyperlipidemia, the 48 remained potential targets with the STRING score values (with the highest confidence 0.9) were mapped onto the DAVID database (https://david.ncifcrf.gov/home.jsp, Version 6.8) to enrich their relevant pathways. As a result, a total of 52 pathways were obtained based on the criterion of *p* < 0.05, which were divided into eight groups such as inflammatory and immune, apoptosis, lipid metabolism, angiogenesis, etc. The detail information of the pathways was presented in **Supplementary Table S6**. Moreover, to visually reflect the relationship between targets and pathways, a T–P network containing 41 targets and 52 pathways classified into 8 groups were mapped, and the results suggested that these targets may affect many pathways to regulate the pathologic processes of hyperlipidemia (**Supplementary Figure S5**). Since lipid metabolism disorder is the core part of hyperlipidemia, we focused on the three pathways closely associated with lipid metabolism, including PPAR signaling pathway, SREBP control of lipid synthesis pathway, and nuclear receptors in lipid metabolism and toxicity pathway (**Table 4**). To further investigate the relationship between components, targets, and the three pathways, a C–T–P network diagram was mapped. As shown in **Figure 4B**, the network diagram displays that six active compounds connected with 10 targets affecting three pathways. These six active compounds are quercetin, emodin, salvianolic acid B, tanshinone II-A, naringenin, and nobiletin (**Figure 5**). The SREBP control of lipid synthesis pathway involves in lipid biosynthesis and LDLR-modulated cholesterol uptake. The PPAR signaling pathway and nuclear receptors in lipid metabolism and toxicity pathway play an important role in the conversion of cholesterol to bile acid and cholesterol efflux. In the three pathways, the targets SREBPs and PPARα are transcription regulators involved in hyperlipidemia-related lipid metabolism processes. SREBP-1c (SREBF-1) and its target gene FAS could regulate the biosynthesis of fatty acids, further influencing the biosynthesis of TGs (Dentin et al., 2005). SREBP-2 (SREBF-2) and its related gene LDLR involved in cholesterol uptake (Kartawijaya et al., 2016). The target PPARα and its related genes LXRα, CYP7A1, and ABCA1 played a regulatory role in the process in the transformation of cholesterol into bile acids and cholesterol efflux (Wang et al., 2001; Basso et al., 2003; Cao et al., 2012; Ramakrishna et al., 2017). Taken together, these results indicated that the lipid-lowering mechanism of DHG may be that the 6 active components (quercetin, emodin, salvianolic acid B, etc.) acted on some important targets such as SREBP-1c, SPEBP-2 and PPARα, which could affect three pathways, including PPAR signaling pathway, SREBP control of lipid synthesis pathway and nuclear receptors in lipid metabolism and toxicity pathway, and further involve in and influence lipid metabolic processes. Besides, some crucial targets, including SREBP-1c, FAS, SREBP-2, LDLR, PPARα, ABCA1, LXRα, and CYP7A1 in the pathways, were selected to investigate the hypolipidemic mechanism of DHG further.

**Table 4:** The detail information of three pathways mainly involved in lipid metabolism.

No.	Pathway	Targets	Count	Relevant targets	*p*-Value	Benjamini	Category	References
1	PPAR signaling pathway	PPARα, APOA1, CD36, OLR1, PPARG	5	RXR, LXRα, CYP7A1, PPARβ, PPARγ, APOAV	6.35E-04	0.005524	Lipid metabolism	(Hansjörg et al., 1993; Wu and Xu, 2016)
2	SREBP control of lipid synthesis	SREBF1, LDLR, SREBF2	3	SCAP, SRE1, HMGCS	0.008613	0.306643	Lipid metabolism	(Eberle et al., 2004; Giudetti et al., 2013)
3	Nuclear receptors in lipid metabolism and toxicity	PPARα, CYP2C9, PPARG, ABCA1	4	LXRα, CYP7A1, CYP27B1, ABCG1, ABCG5	0.024963	0.414386	Lipid metabolism	(Beaven and Tontonoz, 2006)

### Effect of DHG on the Expression of mRNA and Protein of Key Targets

**Figure 6:**
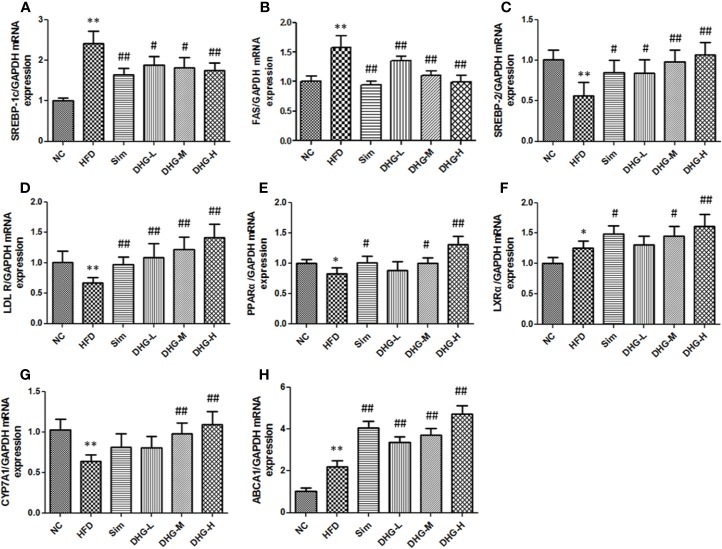
Effect of DHG on mRNA expression of genes in hamster liver by RT-PCR. Values are mean ± SD, n = 5. **(A)** SREBP-1c mRNA. **(B)** FAS mRNA. **(C)** SREBP-2 mRNA. **(D)** LDLR mRNA. **(E)** PPARα mRNA. **(F)** LXRα mRNA. **(G)** CYP7A1 mRNA. **(H)** ABCA1 mRNA. RT-PCR, real-time polymerase chain reaction; SREBP-1c, sterol regulatory element-binding protein-1c; FAS, fatty acid synthase; SREBP-2, sterol regulatory element binding protein 2; LDLR, low density lipoprotein receptor; PPARα, peroxisome proliferation-activated receptor alpha; LXRα, liver X receptor alpha; CYP7A1, cholesterol 7α-hydroxylase; ABCA1, ATP binding cassette subfamily A member 1. ^*^p < 0.05 vs NC; ^**^p < 0.01 vs NC; ^#^*p* < 0.05 vs HFD; ^##^*p* < 0.01 vs HFD.

**Figure 7:**
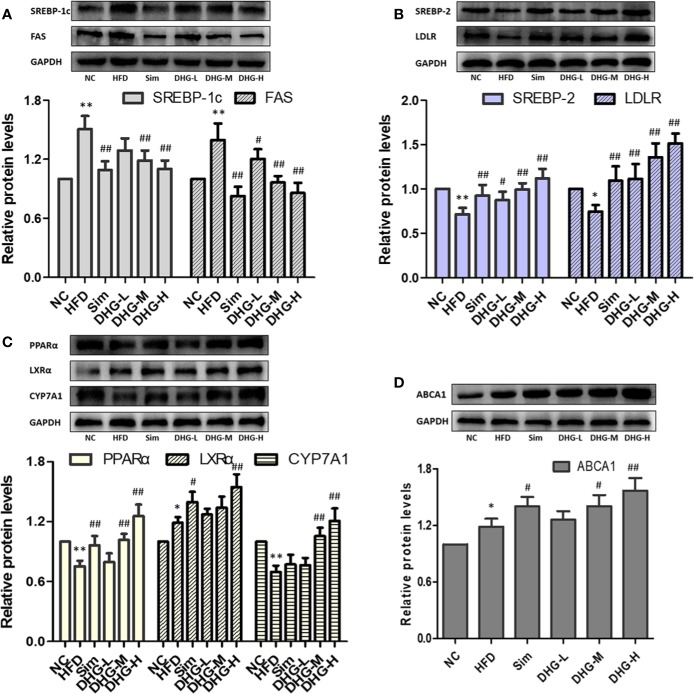
Effect of DHG on protein expression of targets in hamster liver by Western blot. Values are mean ± SD, n = 3. **(A)** SREBP-1c, FAS. **(B)** SREBP-2, LDLR. **(C)** PPARα, LXRα, CYP7A1. **(D)** ABCA1. ^*^p < 0.05, ^**^ *p* < 0.01 vs NC group; ^#^*p* < 0.05, ^##^*p* < 0.01 vs HFD group. The expression levels were normalized by GAPDH.

To elucidate the molecular mechanism of DHG in treating hyperlipidemia, the mRNA and protein expressions of the targets predicted above were measured by RT-PCR and Western blot. The targets SREBP-1c and FAS play an important role in the biosynthesis of fatty acids (Horton, 2002). As shown in **Figures 6A, B**, and **7A**, compared with the NC group, the mRNA and protein expressions of SREBP-1c and FAS in HFD group were significantly increased (*p* < 0.01). Interesting, DHG-M and DHG-H obviously inhibited the mRNA and protein expressions of SREBP-1c and FAS in hyperlipidemic hamster liver (*p* < 0.05). A remarkably lower hepatic mRNA and protein expressions of SREBP-2 and LDLR were observed in HFD group than those in NC group (*p* < 0.05) (**Figures 6C, D**, **7B**). While treated with DHG, the mRNA and protein expression levels of SREBP-2 and LDLR were significantly increased in three DHG groups vs. HFD group (*p* < 0.01). The targets PPARα, LXRα and CYP7A1 could regulate the metabolism of cholesterol transformed into bile acids. As seen in **Figures 6E–G** and **7C**, HFD induced a down-regulation of PPARα and CYP7A1 mRNA and protein expressions (*p* < 0.05), while an up-regulation of LXRα mRNA and protein expressions (*p* < 0.05). After DHG-H treatment for 8 weeks, the mRNA and protein expressions of the PPARα, LXRα, and CYP7A1 were enhanced (*p* < 0.01). ABCA1 plays a major in cholesterol efflux by transporting intracellular cholesterol to the extracellular fluid (Wang and Tall, 2003). As shown in **Figures 6H** and **7D**, the mRNA and protein expression levels of ABCA1 were significantly up-regulated by DHG-M and DHG-H, compared with HFD group (*p* < 0.05).

## Discussion

In China, the TCM formula is a conventional, safe and effective means for treating hyperlipidemia. In our study, DHG, derived from clinical prescriptions Danhe decoction, showed a strong hypolipidemic effect. The results showed that DHG significantly decreased the levels of serum TC, TG, LDL-c, and AI, enhanced the level of serum HDL-c and HDL-c/TC ratio in hyperlipidemic hamsters. Long-term hyperlipidemia may induce atherosclerosis and further cause CVDs such as coronary artery disease. The HDL-c/TC ratio could predict the risk of coronary artery disease and the atherosclerosis index (AI) was also usually taken as the indicator of the risk of atherosclerosis (Yang et al., 2008; Sathiya et al., 2014). It is noteworthy that DHG-H was more effective than Sim in elevating levels of serum HDL-c and HDL-c/TC ratio, lowering the AI value, which suggested DHG has the potential application in inhibiting coronary artery disease and atherosclerosis. Besides, DHG treatment not only inhibited the fat accumulation in the liver, but also inversed the increase of body weight gain, liver index, and related adipose tissue weight in hyperlipidemic hamsters, which further confirmed that the DHG had a beneficial lipid-lowering effect.

Systems pharmacology has gradually become an effective method to study the active components and mechanisms of action of TCM (Zhou et al., 2019; Zhang et al., 2019). Different from most of the previous studies that usually consider ingredients from herbal databases, we take the compounds that are detected in the hamster blood as the compound source to carry out systems pharmacology research. In the present study, the 16 prototype compounds, such as polydatin, nuciferine and hyperin were identified from the hamster serum, which are the first report of the chemical constituents of DHG *in vivo*. Moreover, focusing on these ingredients absorbed into the blood, further systems pharmacology results indicated that 6 active compounds, including quercetin, emodin, salvianolic acid B, tanshinone II-A, naringenin, and nobiletin, may modulate some key targets (SREBP-1c, SPEBP-2, PPARα, and so on), involved in 3 closely lipid metabolism-related pathways. These six active compounds may be the potential material basis for the lipid-lowering effect of DHG. Previous studies have shown that quercetin, naringenin, and nobiletin, as flavonoids, are known for their protection against CVDs though lowering hepatic lipid accumulation, inhibiting hepatic fatty acid synthesis and increasing fatty acid oxidation (Jung et al., 2013; Hil et al., 2014; Mulvihill et al., 2016; Zhang et al., 2018). Emodin, as anthraquinones, could regulate lipid metabolism *via* the inhibition of lipid accumulation and lipogenesis (Tzeng et al., 2012; Cao et al., 2016). Salvianolic acid B and tanshinone II-A could attenuate atherosclerotic lesions by reducing vascular oxidative stress or regulating lipid metabolism (Xu et al., 2011; Zhang et al., 2011; Yue et al., 2015). Moreover, for PPAR signaling pathway and nuclear receptors in lipid metabolism and toxicity pathway, PPARα plays a role in scavenging circulating or cellular lipids by forming heterodimers with retinoid X receptor and regulating the expressions of genes involved in lipid metabolism such as biosynthesis of bile acids, cholesterol efflux, and so on (Ogata et al., 2009; Filip-Ciubotaru et al., 2011; Ramakrishna et al., 2017). In SREBP control of lipid synthesis pathway, SREBPs could bind to the LDL receptor promoter to enhance the expression of LDLR on cell surface to promote the internalization of plasma LDL (Ochiai et al., 2015).

**Figure 8:**
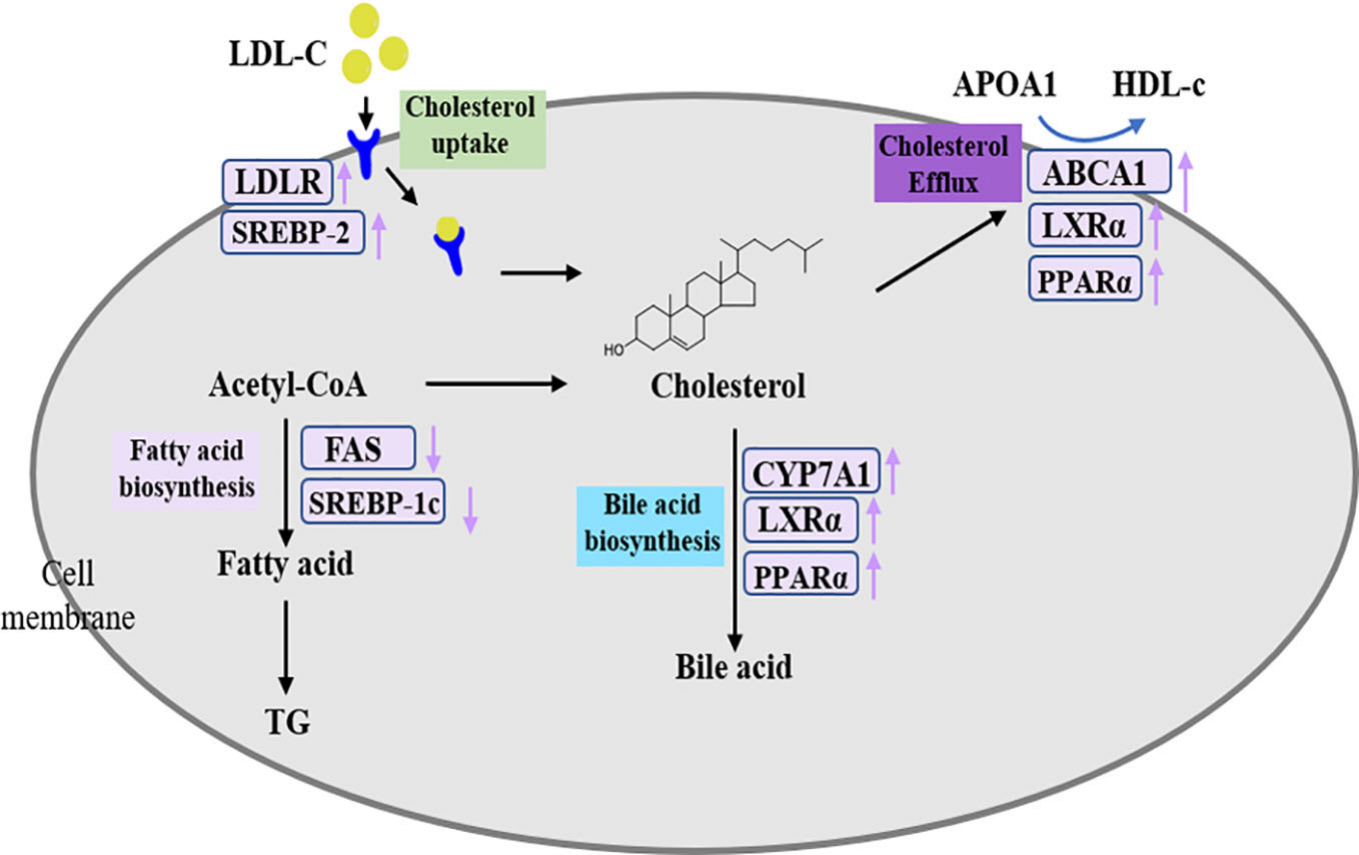
The proposed mechanism of DHG in treatment of hyperlipidemia.

The SREBPs are important transcription factors that regulate lipid homeostasis by regulating the expressions of related genes in cholesterol homeostasis, fatty acid synthesis, and TG metabolism. SREBP-1c preferentially activates genes of fatty acid and TG metabolism, whereas SREBP-2 preferentially activates genes of cholesterol metabolism (Horton, 2002). Fatty acids, as raw materials for the synthesis of TGs, are closely related to the synthesis of TGs. FAS directly catalyzed the biosynthesis of fatty acids and modulated by SREBP-1c. The systems pharmacology results showed that the target SREBP-1c could be regulated by naringenin. Meanwhile, researchers have found that naringenin could inhibit hepatic fatty acids synthesis by decreasing SREBP-1c and other related genes mRNA levels in rats (Hashimoto and Ide, 2015). Moreover, our results indicated that DHG suppressed the SREBP-1c and FAS mRNA and protein expressions in hyperlipidemic hamsters, which could contribute to explain the effects of DHG on lowering serum TG level and inhibiting hepatic lipid accumulation. In humans, LDL receptor-mediated uptake and HDL-mediated reverse transports are important transport methods responsible for nearly 60-70% total plasma cholesterol (Zhang et al., 2013; Ridker, 2014). In general, LDL-c is removed from the circulation mainly by liver uptake *via* LDLR that internalizes bound LDL particles through endocytosis. The increase of LDLR expression will enhance the uptake and removal of LDL-c. LDLR is a known target gene of SREBP-2 (Kartawijaya et al., 2016). By C-T-P network mapping, we discovered that the target SREBP-2 could be modulated by quercetin and LDLR is connected to naringenin, nobiletin, quercetin and tanshinone II-A. Research has revealed quercetin could induce SREBP-2 and LDLR expression in hepG2 cells (Moon et al., 2012). In addition, naringenin, nobiletin, and tanshinone II-A were also reported to up-regulate the LDLR expression to affect lipid metabolism (Morin et al., 2008; Chen et al., 2016; Bawazeer et al., 2017). In the present study, our results suggested that DHG treatment increased the SREBP-2 and LDLR mRNA and protein expressions, which may be the main reason for the reduction of serum LDL-c effect by DHG. PPARα is a transcription factor that belongs to the nuclear receptor superfamily and plays important roles in metabolic regulation and affects the different links of lipid metabolism, including fatty acid uptake, fatty acid oxidation, cholesterol elimination and transport, etc. (Chinetti et al., 2001; Ogata et al., 2009). From the C-T-P network, PPARα is targeted by emodin and salvianolic acid B. Previous investigators have demonstrated that the two active compounds could regulate the expression of the target PPARα (Liu et al., 2010; Huang et al., 2016). CYP7A1, the rate-limiting enzyme in the classical bile acid biosynthetic pathway, catalyzing about 2/5 synthesized cholesterol transformed into bile acids, has a critical function in keeping homeostasis of cholesterol (Li et al., 2011; Cao et al., 2012). It is reported that PPARα and LXRα mediate feed-forward induction of CYP7A1, contributing to the clearance of excess cholesterol by increasing the formation of bile acids (Beaven and Tontonoz, 2006; Singh et al., 2013; Ramakrishna et al., 2017). In this study, DHG-H treatment significantly increased the mRNA and protein expressions of PPARα, LXRα, and CYP7A1, and enhanced the fecal TC and TBA levels in hyperlipidemic hamsters. These results suggested that the lipid-lowering effects of DHG may be related to the biosynthesis of bile acids. ABCA1, a trans-membranous protein, plays a key role in the regulation of cholesterol efflux (Li et al., 2018). The chief functions of ABCA1 are transporting intracellular cholesterol to the extracellular fluid and reducing the level of intracellular cholesterol by uniting it with HDL, which is regulated in a PPARα-LXRα-ABCA1 dependent manner (Hossain et al., 2008; Liu et al., 2015; Vijayakumar and Nachiappan, 2017). Based on the C-T-P network, the target ABCA1 is connected with quercetin. Quercetin demonstrated a protective effect against atherosclerosis partly through increasing the expression of ABCA1 (Jia et al., 2019). Our results showed that DHG up-regulated the mRNA and protein expressions of PPARα, LXRα, and ABCA1, which could help to explain the HDL-c-elevating effect of DHG. In summary, as shown in **Figure 8**, DHG may exert a hypolipidemic effect by affecting some targets such as some targets, such as SREBP-1c, SREBP-2 and PPARα, and further involving in lipid metabolic processes including fatty acid biosynthesis, LDLR-mediated cholesterol uptake, bile acid biosynthesis and cholesterol efflux.

Regardless, this study still has some limitations. First, we did not carry out experimental research on the targets of monomer active ingredients. Moreover, other species should also be considered in future experimental study.

## Conclusion

In this study, DHG exhibited excellent hypolipidemic effects in HFD-induced hyperlipidemic hamsters by multiple aspects, including improving blood lipid, reducing liver fat accumulation, and lowering body weight gain and liver index, etc. Then, systems pharmacology investigations focusing on the 16 main compounds absorbed into blood after oral administration of DHG showed that 6 active compounds, namely, quercetin, emodin, salvianolic acid B, tanshinone II-A, naringenin, and nobiletin, may affect some key targets such as SREBP-1c, SREBP-2 and PPARα, which were involved in three closely lipid metabolism-related pathways including PPAR signaling pathway, SREBP control of lipid synthesis pathway, and nuclear receptors in lipid metabolism and toxicity pathway. Further studies indicated that the hypolipidemic mechanisms of DHG were associated with the up-regulation of SREBP-2, LDLR, PPARα, LXRα, CYP7A1, and ABCA1 mRNA and protein expression levels, the down-regulation of SREBP-1c and FAS mRNA and protein expression levels. This work demonstrated that DHG has a good hypolipidemic effect, and provided an efficient way to understand the active ingredients and underlying mechanisms of DHG.

## Data Availability Statement

All datasets generated for this study are included in the article/**Supplementary Material**.

## Ethics Statement

The animal study was reviewed and approved by the Ethics Committee of Beijing University of Traditional Chinese Medicine (Beijing, China). This study was carried out in accordance with the principles of the Basel Declaration and recommendations of guidelines of the National Institutes of Health Conflict of Interest.

## Author Contributions

KC, HX, JL, and WX conceived and designed the experiments. KC and ZM performed the experiments. ZM and XY analyzed the data. YL, YD, and YZ contributed reagents/materials/analysis tools. KC and XY wrote and edited the paper.

## Funding

This work was financially supported by the National Science and Technology Major Project (2019ZX09201004-001), the National Natural Science Foundation of China (No. 81573839 and No. 81774155), and the Fundamental Research Funds for the Central Universities (No. 2018-JYB-XS058).

## Conflict of Interest

The authors declare that the research was conducted in the absence of any commercial or financial relationships that could be construed as a potential conflict of interest.

## Abbreviations

DHG, Danhe granule; UHPLC-Q-TOF/MS, ultra-high performance liquid chromatography coupled with quadrupole time-of-flight mass spectrometry; GO enrichment, gene ontology enrichment; RT-PCR, real-time polymerase chain reaction; TC, total cholesterol; TG, triglyceride; LDL-c, low-density lipoprotein cholesterol; HDL-c, high-density lipoprotein cholesterol; NC, normal control group; HFD, high-fat diet group; Sim, simvastatin; DHG-L, DHG at low dosage; DHG-M, DHG at medium dosage; DHG-H, DHG at high dosage; SREBP, sterol regulatory element binding proteins; SREBP-1c, sterol regulatory element-binding protein-1c; FAS, fatty acid synthase; SREBP-2, sterol regulatory element binding protein 2; LDLR, low-density lipoprotein receptor; PPARα, peroxisome proliferation-activated receptor alpha; LXRα, liver X receptor alpha; CYP7A1, cholesterol 7α-hydroxylase; ABCA1, ATP binding cassette subfamily A member 1; CVD, cardiovascular disease; TCM, traditional Chinese medicine; TBA, total bile acid.
