# Supplementary material for: Investigation of the Lipid-Lowering Mechanisms and Active Ingredients of Danhe Granule on Hyperlipidemia Based on Systems Pharmacology
Source: Front Pharmacol. 2020 May 6;11:528. doi: 10.3389/fphar.2020.00528 (PMC7218108; doi:10.3389/fphar.2020.00528)
Supplement: Supplementary file 1 [file DataSheet_1.docx]

Table S1. Detailed information of the crude drugs composed in DHG.

| Voucher number | Name | Authentication | Origin | Voucher specimen |
| --- | --- | --- | --- | --- |
| No. CMAT-SM-201806 | Saliae Mil Tiorrhizae Radix et Rhizoma | Root and rhizome of *Salvia miltiorrhiza* Bunge | Anguo City, Heibei, China | 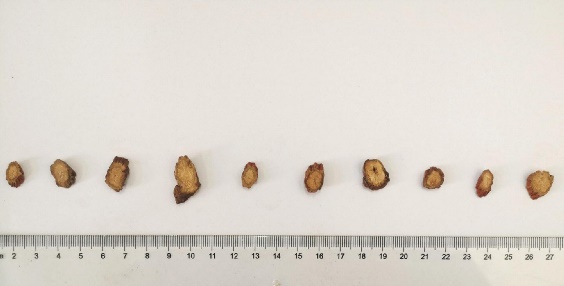 |
| NO. CMAT-PC-201809 | Polygoni Cuspidati Rhizoma et Radix | Root and rhizome of *Reynoutria japonica* Houtt. | Xuzhou City, Jiangsu, China | 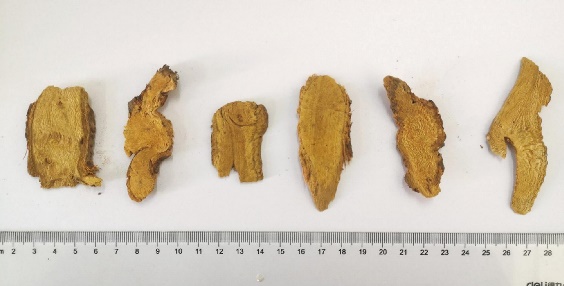 |
| NO. CMAT-CP-201810 | Crataegi Fructus | Fruit of *Crataegus pinnatifida* Bunge | Chengde City, Hebei, China | 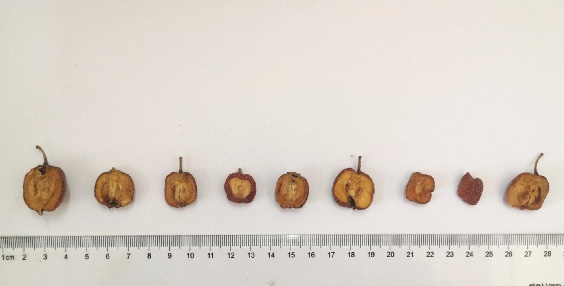 |
| NO. CMAT-CR-201811 | Citri Reticulatae Pericarpium | Peel of *Citrus × aurantium* L. | Deyang City, Sichuan, China | 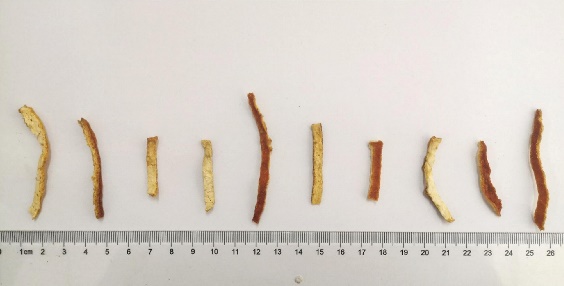 |
| NO. CMAT-CL-201808 | Coicis Semen | kernel of *Coix lacryma-jobi var. ma-yuen* (Rom.Caill.) Stapf | Anguo City, Hebei, China | 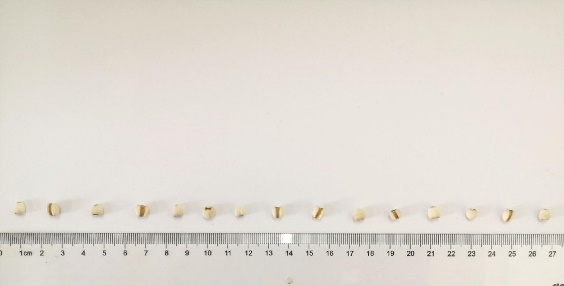 |
| NO. CMAT-NN-201807 | Nelumbinis Folium | Leaf of *Nelumbo nucifera* Gaertn. | Anxin City, Hebei, China | 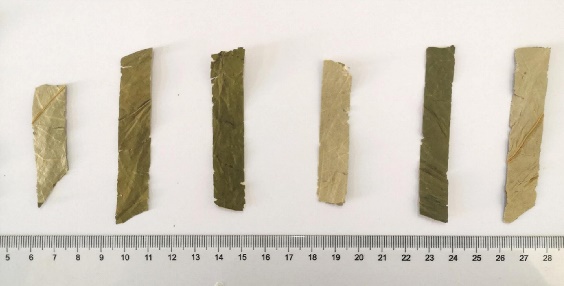 |

Table S2. The primer sequences of the target genes

| No. | Gene | Primer sequence（5’ to 3’） | | |
| --- | --- | --- | --- | --- |
|  |  | Forward | | Reverse |
| 1 | SREBP-1c | GCACTTTTTGACACGTTTCTTC | | CTGTACAGGCTCTCCTGTGG |
| 2 | FAS | AATTGCGGCTGCGTGGATATGA | | GTTGGCTGAGACCCAGAACGGATAC |
| 3 | SREBP-2 | GCAGGTACAGCCAGTCACGAT | TGGTCAAAACAAGGGAATCTG | |
| 4 | LDLR | ATCGCTCTGGTCATCCTCCTTGTCT | | TTCGTCTTCCGTGGTCTTCTGGTAC |
| 5 | PPARα | TGCCTGTCTGTTGGGATGT | | TGGCTTCATACACACCGTACTT |
| 6 | LXRα | TCAGCATCTTCTCTGCAGACCGG | | TCATTAGCATCCGTGGGAACA |
| 7 | CYP7A1 | ACACCATTCCTGCAACCTTC | | GCTGTCCGGATATTCAAGGA |
| 8 | ABCA1 | TCCAGCTCCTCCACCCAGATATACC | | TAGTTGTTGTCCTCGTACCAGTTGA |
| 9 | GAPDH | GTGCCGAGTATGTTGTGGAGT | | GTGTGGAGATGATGACCCTATTG |

Table S3 the information of 969 targets of compounds obtained from Genecards Databases.

| NO. | Compound | Gene Symbol | NO. | Compound | Gene Symbol | NO. | Compound | Gene Symbol |
| --- | --- | --- | --- | --- | --- | --- | --- | --- |
| 1 | Emodin | CSNK2A1 | 324 | Tanshinone II-A | PRKAA1 | 647 | Quercetin | TP53 |
| 2 | Emodin | NFKB1 | 325 | Tanshinone II-A | NR1I2 | 648 | Quercetin | BAK1 |
| 3 | Emodin | CASP3 | 326 | Tanshinone II-A | NCF2 | 649 | Quercetin | SULT1E1 |
| 4 | Emodin | BCL2 | 327 | Tanshinone II-A | G6PD | 650 | Quercetin | ABCG2 |
| 5 | Emodin | TP53 | 328 | Tanshinone II-A | PRKAA2 | 651 | Quercetin | TFF1 |
| 6 | Emodin | MMP2 | 329 | Tanshinone II-A | FOXM1 | 652 | Quercetin | FASN |
| 7 | Emodin | CDKN1A | 330 | Tanshinone II-A | H6PD | 653 | Quercetin | ALOX5 |
| 8 | Emodin | BAX | 331 | Tanshinone II-A | CHUK | 654 | Quercetin | PTPN3 |
| 9 | Emodin | MMP9 | 332 | Tanshinone II-A | AKR1B1 | 655 | Quercetin | TYMS |
| 10 | Emodin | ERBB2 | 333 | Tanshinone II-A | PTK2 | 656 | Quercetin | AGK |
| 11 | Emodin | AHR | 334 | Tanshinone II-A | CCN2 | 657 | Quercetin | POLB |
| 12 | Emodin | CYCS | 335 | Tanshinone II-A | NAMPT | 658 | Quercetin | GFAP |
| 13 | Emodin | MAPK1 | 336 | Tanshinone II-A | PRKAB1 | 659 | Quercetin | AKR1B1 |
| 14 | Emodin | CCNB1 | 337 | Tanshinone II-A | OXA1L | 660 | Quercetin | F3 |
| 15 | Emodin | OXA1L | 338 | Tanshinone II-A | DDIT3 | 661 | Quercetin | LOX |
| 16 | Emodin | NFKB2 | 339 | Tanshinone II-A | PLAU | 662 | Quercetin | PIK3R1 |
| 17 | Emodin | ERCC1 | 340 | Tanshinone II-A | NR1I3 | 663 | Quercetin | CCL2 |
| 18 | Emodin | RAD51 | 341 | Tanshinone II-A | IKBKG | 664 | Quercetin | TNFSF10 |
| 19 | Emodin | MAPK14 | 342 | Tanshinone II-A | KCNMA1 | 665 | Quercetin | CCNA2 |
| 20 | Emodin | CDK1 | 343 | Tanshinone II-A | HSPA5 | 666 | Quercetin | IFI27 |
| 21 | Emodin | CASP9 | 344 | Tanshinone II-A | CYP2A6 | 667 | Quercetin | NR1H2 |
| 22 | Emodin | PPARG | 345 | Tanshinone II-A | TNFSF10 | 668 | Quercetin | GADD45A |
| 23 | Emodin | ABCC1 | 346 | Tanshinone II-A | LRP1 | 669 | Quercetin | CDK2 |
| 24 | Emodin | IL10 | 347 | Tanshinone II-A | EIF4EBP1 | 670 | Quercetin | POR |
| 25 | Emodin | TGFB1 | 348 | Tanshinone II-A | MMP7 | 671 | Quercetin | SELE |
| 26 | Emodin | CHUK | 349 | Tanshinone II-A | PKM | 672 | Quercetin | EGFR |
| 27 | Emodin | AR | 350 | Tanshinone II-A | MCL1 | 673 | Quercetin | CYP2C9 |
| 28 | Emodin | PARP1 | 351 | Tanshinone II-A | AQP1 | 674 | Quercetin | CHUK |
| 29 | Emodin | ILK | 352 | Tanshinone II-A | TIMP2 | 675 | Quercetin | DNTT |
| 30 | Emodin | TNF | 353 | Tanshinone II-A | PPIG | 676 | Quercetin | MME |
| 31 | Emodin | BOK | 354 | Tanshinone II-A | PLA2G4A | 677 | Quercetin | OLR1 |
| 32 | Emodin | CHEK2 | 355 | Tanshinone II-A | MIR122 | 678 | Quercetin | PTGS2 |
| 33 | Emodin | PIK3CG | 356 | Tanshinone II-A | PTGS1 | 679 | Quercetin | IPMK |
| 34 | Emodin | BIRC5 | 357 | Tanshinone II-A | MMP12 | 680 | Quercetin | CASP1 |
| 35 | Emodin | PLAU | 358 | Tanshinone II-A | PHB | 681 | Quercetin | CYP2D6 |
| 36 | Emodin | CRP | 359 | Tanshinone II-A | GAS5 | 682 | Quercetin | PTEN |
| 37 | Emodin | NFKBIA | 360 | Tanshinone II-A | CYP2J2 | 683 | Quercetin | ALPP |
| 38 | Emodin | CCNA2 | 361 | Tanshinone II-A | SERPINB5 | 684 | Quercetin | UGT2B7 |
| 39 | Emodin | LRP1 | 362 | Tanshinone II-A | RPS6 | 685 | Quercetin | UGT1A4 |
| 40 | Emodin | ALB | 363 | Tanshinone II-A | CSF1R | 686 | Quercetin | IP6K2 |
| 41 | Emodin | HSPA4 | 364 | Tanshinone II-A | GPX1 | 687 | Quercetin | AKR1C3 |
| 42 | Emodin | RHOA | 365 | Tanshinone II-A | AQP5 | 688 | Quercetin | CDKN1A |
| 43 | Emodin | IFNA1 | 366 | Tanshinone II-A | MIR27A | 689 | Quercetin | APOB |
| 44 | Emodin | CXCR4 | 367 | Tanshinone II-A | XYLT1 | 690 | Quercetin | NR1I2 |
| 45 | Emodin | VEGFA | 368 | Tanshinone II-A | MAPK9 | 691 | Quercetin | UGT2B4 |
| 46 | Emodin | CASP8 | 369 | Tanshinone II-A | YWHAQ | 692 | Quercetin | CSNK2A1 |
| 47 | Emodin | CHGA | 370 | Tanshinone II-A | PSMC4 | 693 | Quercetin | UGT1A3 |
| 48 | Emodin | KRT20 | 371 | Tanshinone II-A | DLX5 | 694 | Quercetin | SLC16A1 |
| 49 | Emodin | NOS2 | 372 | Tanshinone II-A | ELAVL1 | 695 | Quercetin | NQO2 |
| 50 | Emodin | EGFR | 373 | Tanshinone II-A | C5AR1 | 696 | Quercetin | TMPO |
| 51 | Emodin | GSK3B | 374 | Tanshinone II-A | ITGAV | 697 | Quercetin | PLAT |
| 52 | Emodin | HSP90AA1 | 375 | Tanshinone II-A | PSMC6 | 698 | Quercetin | MCL1 |
| 53 | Emodin | CAPN1 | 376 | Tanshinone II-A | NFKB2 | 699 | Quercetin | CYP2B6 |
| 54 | Emodin | SOD1 | 377 | Tanshinone II-A | CCR2 | 700 | Quercetin | PLAU |
| 55 | Emodin | CAT | 378 | Tanshinone II-A | MIR137 | 701 | Quercetin | PTK2 |
| 56 | Emodin | CASP1 | 379 | Tanshinone II-A | AKR1B10 | 702 | Quercetin | EGF |
| 57 | Emodin | GDNF | 380 | Tanshinone II-A | ITGB5 | 703 | Quercetin | SYK |
| 58 | Emodin | CD36 | 381 | Tanshinone II-A | LOC110366354 | 704 | Quercetin | IRS1 |
| 59 | Emodin | ATF1 | 382 | Tanshinone II-A | HCN2 | 705 | Quercetin | CSNK2B |
| 60 | Emodin | PLAUR | 383 | Tanshinone II-A | TPT1 | 706 | Quercetin | CXCL8 |
| 61 | Emodin | PRKD1 | 384 | Tanshinone II-A | EFS | 707 | Quercetin | CHRNA7 |
| 62 | Emodin | FGFR2 | 385 | Tanshinone II-A | CRYZ | 708 | Quercetin | CFTR |
| 63 | Emodin | STAT3 | 386 | Tanshinone II-A | PARP4 | 709 | Quercetin | NGF |
| 64 | Emodin | NPM1 | 387 | Tanshinone II-A | BDKRB1 | 710 | Quercetin | ICAM1 |
| 65 | Emodin | TRIB3 | 388 | Tanshinone II-A | KCNE2 | 711 | Quercetin | FOS |
| 66 | Emodin | MYC | 389 | Tanshinone II-A | IRAK1 | 712 | Quercetin | EDN1 |
| 67 | Emodin | JUN | 390 | Naringenin | CYP1B1 | 713 | Quercetin | SLCO2B1 |
| 68 | Emodin | ERCC4 | 391 | Naringenin | ESR1 | 714 | Quercetin | PIK3CD |
| 69 | Emodin | SNAI2 | 392 | Naringenin | APOB | 715 | Quercetin | PIK3CB |
| 70 | Emodin | P2RX7 | 393 | Naringenin | HMGCR | 716 | Quercetin | PLCG2 |
| 71 | Emodin | RPA1 | 394 | Naringenin | MTTP | 717 | Quercetin | SETD2 |
| 72 | Emodin | XPA | 395 | Naringenin | CYP2A6 | 718 | Quercetin | APP |
| 73 | Emodin | FGFR1 | 396 | Naringenin | ABCB1 | 719 | Quercetin | ERBB2 |
| 74 | Emodin | PDGFRB | 397 | Naringenin | UGT1A1 | 720 | Quercetin | TGFB1 |
| 75 | Emodin | KDR | 398 | Naringenin | LDLR | 721 | Quercetin | PRKAB1 |
| 76 | Emodin | DNMT3A | 399 | Naringenin | KCNH2 | 722 | Quercetin | AIFM1 |
| 77 | Emodin | COL1A1 | 400 | Naringenin | CES1 | 723 | Quercetin | VEGFA |
| 78 | Emodin | COL3A1 | 401 | Naringenin | SULT1A3 | 724 | Quercetin | TNF |
| 79 | Emodin | CDKN3 | 402 | Naringenin | CYP1A1 | 725 | Quercetin | DHRS11 |
| 80 | Emodin | UHRF1 | 403 | Naringenin | NOS2 | 726 | Quercetin | MMP9 |
| 81 | Emodin | PTGS2 | 404 | Naringenin | ABCC1 | 727 | Quercetin | ERVK-6 |
| 82 | Emodin | UGT1A1 | 405 | Naringenin | GUSB | 728 | Quercetin | ABCC4 |
| 83 | Emodin | AIFM1 | 406 | Naringenin | GSR | 729 | Quercetin | PPARG |
| 84 | Emodin | PIK3R1 | 407 | Naringenin | ESR2 | 730 | Quercetin | PIK3R2 |
| 85 | Emodin | CDKN2A | 408 | Naringenin | POR | 731 | Quercetin | PIK3C3 |
| 86 | Emodin | GRB2 | 409 | Naringenin | GBA3 | 732 | Quercetin | PIK3R4 |
| 87 | Emodin | FN1 | 410 | Naringenin | ABCG2 | 733 | Quercetin | PIK3C2B |
| 88 | Emodin | CASP2 | 411 | Naringenin | CYP19A1 | 734 | Quercetin | PIK3R5 |
| 89 | Emodin | CCL2 | 412 | Naringenin | AHR | 735 | Quercetin | PIK3C2A |
| 90 | Emodin | BCL2L1 | 413 | Naringenin | TFF1 | 736 | Quercetin | PIK3R3 |
| 91 | Emodin | MMP7 | 414 | Naringenin | UGT2B15 | 737 | Quercetin | PIK3C2G |
| 92 | Emodin | PTK2 | 415 | Naringenin | SLCO2B1 | 738 | Quercetin | DNASE1 |
| 93 | Emodin | SOS1 | 416 | Naringenin | AKR1C1 | 739 | Quercetin | EGR1 |
| 94 | Emodin | MAP3K3 | 417 | Naringenin | SHBG | 740 | Quercetin | CLDN4 |
| 95 | Emodin | KLK3 | 418 | Naringenin | CYP3A4 | 741 | Quercetin | TNFRSF10B |
| 96 | Emodin | CCR1 | 419 | Naringenin | KANSL3 | 742 | Quercetin | HCK |
| 97 | Emodin | BAK1 | 420 | Naringenin | SLCO1A2 | 743 | Quercetin | PIM1 |
| 98 | Emodin | RASSF1 | 421 | Naringenin | BCL2 | 744 | Quercetin | ACTB |
| 99 | Emodin | PENK | 422 | Naringenin | SOAT2 | 745 | Quercetin | SLCO1B1 |
| 100 | Emodin | AKT1 | 423 | Naringenin | SOD1 | 746 | Quercetin | UBA1 |
| 101 | Emodin | CTNNB1 | 424 | Naringenin | INSR | 747 | Quercetin | HIBCH |
| 102 | Emodin | XIAP | 425 | Naringenin | PPARG | 748 | Quercetin | HSPA2 |
| 103 | Emodin | TWIST1 | 426 | Naringenin | PIK3R1 | 749 | Quercetin | CEBPB |
| 104 | Emodin | CXCL8 | 427 | Naringenin | INS | 750 | Quercetin | RUVBL2 |
| 105 | Emodin | HIF1A | 428 | Naringenin | SIK2 | 751 | Quercetin | EIF3F |
| 106 | Emodin | IKBKG | 429 | Naringenin | MMP2 | 752 | Quercetin | SHBG |
| 107 | Emodin | NAT1 | 430 | Naringenin | SREBF1 | 753 | Quercetin | STK17B |
| 108 | Emodin | PTEN | 431 | Naringenin | AKT1 | 754 | Quercetin | SLC16A7 |
| 109 | Emodin | SLC31A1 | 432 | Naringenin | NOS3 | 755 | Quercetin | SF3B3 |
| 110 | Emodin | SYP | 433 | Naringenin | CAT | 756 | Quercetin | UGT3A1 |
| 111 | Emodin | PTP4A3 | 434 | Naringenin | FAS | 757 | Quercetin | CINP |
| 112 | Emodin | MPO | 435 | Naringenin | CASP9 | 758 | Quercetin | ATP5F1A |
| 113 | Emodin | MAPK8 | 436 | Naringenin | ERN1 | 759 | Quercetin | ATP5F1B |
| 114 | Emodin | CD79A | 437 | Naringenin | TYR | 760 | Quercetin | ATP5F1C |
| 115 | Emodin | SLC17A5 | 438 | Naringenin | CTNNB1 | 761 | Quercetin | PRKCA |
| 116 | Emodin | BCR | 439 | Naringenin | MAPT | 762 | Quercetin | CFLAR |
| 117 | Emodin | FASN | 440 | Naringenin | APP | 763 | Quercetin | CASP7 |
| 118 | Emodin | BID | 441 | Naringenin | FASN | 764 | Quercetin | ACACA |
| 119 | Emodin | ARAF | 442 | Naringenin | PARP1 | 765 | Quercetin | COX5A |
| 120 | Emodin | HMGB1 | 443 | Naringenin | P2RY12 | 766 | Quercetin | SLC12A2 |
| 121 | Emodin | RNF34 | 444 | Naringenin | F2R | 767 | Quercetin | NOS3 |
| 122 | Emodin | RFFL | 445 | Naringenin | TCF7L2 | 768 | Quercetin | ALDH1A1 |
| 123 | Emodin | LOC107963955 | 446 | Naringenin | MAPK1 | 769 | Quercetin | HSPA5 |
| 124 | Emodin | LOC107980440 | 447 | Naringenin | G6PD | 770 | Quercetin | CD36 |
| 125 | Emodin | P4HB | 448 | Naringenin | LOC110599580 | 771 | Quercetin | CTSD |
| 126 | Emodin | VIM | 449 | Naringenin | CASP3 | 772 | Quercetin | SNAI1 |
| 127 | Emodin | HSPD1 | 450 | Naringenin | SOAT1 | 773 | Quercetin | XBP1 |
| 128 | Emodin | CDC25A | 451 | Naringenin | CYP1A2 | 774 | Quercetin | TP73 |
| 129 | Emodin | ENDOG | 452 | Naringenin | CYB5A | 775 | Quercetin | IL6 |
| 130 | Emodin | IFI27 | 453 | Naringenin | MIR17 | 776 | Quercetin | MAP3K5 |
| 131 | Emodin | CDK2 | 454 | Naringenin | BAX | 777 | Quercetin | EIF2S1 |
| 132 | Emodin | TAS2R43 | 455 | Naringenin | CASP8 | 778 | Quercetin | PON2 |
| 133 | Emodin | TAS2R31 | 456 | Naringenin | STAT1 | 779 | Quercetin | IKBKB |
| 134 | Emodin | WEE1 | 457 | Naringenin | AIFM1 | 780 | Quercetin | MAPK10 |
| 135 | Emodin | BACE1 | 458 | Naringenin | NQO1 | 781 | Quercetin | MYC |
| 136 | Emodin | UGT2B7 | 459 | Naringenin | CAV1 | 782 | Quercetin | SLC17A5 |
| 137 | Emodin | UGT1A9 | 460 | Naringenin | NFE2L2 | 783 | Quercetin | PPIG |
| 138 | Emodin | UGT1A3 | 461 | Naringenin | POLB | 784 | Quercetin | PCNA |
| 139 | Emodin | RHOB | 462 | Naringenin | OGG1 | 785 | Quercetin | SLC2A1 |
| 140 | Emodin | CFTR | 463 | Naringenin | GCLC | 786 | Quercetin | ERN1 |
| 141 | Emodin | MMP1 | 464 | Naringenin | GCLM | 787 | Quercetin | KNG1 |
| 142 | Emodin | ABCB1 | 465 | Naringenin | INTS3 | 788 | Quercetin | CREBBP |
| 143 | Emodin | MMP3 | 466 | Naringenin | SULT2B1 | 789 | Quercetin | NFKB1 |
| 144 | Emodin | APOE | 467 | Naringenin | ADORA2A | 790 | Quercetin | CYBA |
| 145 | Emodin | APP | 468 | Naringenin | ADORA3 | 791 | Quercetin | MTTP |
| 146 | Emodin | FAS | 469 | Naringin | SLCO1A2 | 792 | Quercetin | CSF2 |
| 147 | Emodin | CYP3A4 | 470 | Naringin | HMGCR | 793 | Quercetin | EP300 |
| 148 | Emodin | CYP2C9 | 471 | Naringin | CYP3A4 | 794 | Quercetin | LOC106560211 |
| 149 | Emodin | IL6 | 472 | Naringin | CYP2A6 | 795 | Quercetin | APOE |
| 150 | Emodin | CYP2C8 | 473 | Naringin | GUSB | 796 | Quercetin | APOA1 |
| 151 | Emodin | CYP2D6 | 474 | Naringin | CYP1A1 | 797 | Quercetin | IFNG |
| 152 | Emodin | IL1B | 475 | Naringin | BMP2 | 798 | Quercetin | KEAP1 |
| 153 | Emodin | IL1RN | 476 | Naringin | ABCB1 | 799 | Quercetin | CHEK2 |
| 154 | Emodin | LEP | 477 | Naringin | ALPP | 800 | Quercetin | MMP2 |
| 155 | Emodin | ALOX5 | 478 | Naringin | PIK3R1 | 801 | Quercetin | TLR4 |
| 156 | Emodin | AKR1B1 | 479 | Naringin | CASP3 | 802 | Quercetin | TJP2 |
| 157 | Emodin | CYP3A5 | 480 | Naringin | FOS | 803 | Quercetin | NAT2 |
| 158 | Emodin | COL2A1 | 481 | Naringin | JUN | 804 | Quercetin | EPO |
| 159 | Emodin | CYP2C19 | 482 | Naringin | DNAH8 | 805 | Quercetin | NOTCH1 |
| 160 | Emodin | TNFRSF1B | 483 | Naringin | MMP2 | 806 | Quercetin | SRC |
| 161 | Emodin | AKR1C2 | 484 | Naringin | MMP9 | 807 | Quercetin | GCLC |
| 162 | Emodin | FAAH | 485 | Naringin | MAPK14 | 808 | Quercetin | LDLR |
| 163 | Emodin | BDNF | 486 | Naringin | PARP1 | 809 | Quercetin | FYN |
| 164 | Emodin | ABCC4 | 487 | Naringin | CASP9 | 810 | Quercetin | FASLG |
| 165 | Emodin | PTGS1 | 488 | Naringin | CSK | 811 | Quercetin | BMP2 |
| 166 | Emodin | AQP2 | 489 | Naringin | NDUFS4 | 812 | Quercetin | RUNX2 |
| 167 | Emodin | VKORC1 | 490 | Naringin | CASP1 | 813 | Quercetin | SPP1 |
| 168 | Emodin | IL1R1 | 491 | Naringin | PRKACA | 814 | Quercetin | TNFRSF10D |
| 169 | Emodin | PLA2G2A | 492 | Naringin | GNMT | 815 | Quercetin | PSMC4 |
| 170 | Emodin | VCAM1 | 493 | Nobiletin | MAPK1 | 816 | Quercetin | MUC5AC |
| 171 | Emodin | LOX | 494 | Nobiletin | JUN | 817 | Quercetin | MIR34A |
| 172 | Emodin | GP1BA | 495 | Nobiletin | TIMP1 | 818 | Quercetin | CDKN2A |
| 173 | Emodin | F3 | 496 | Nobiletin | MAPK8 | 819 | Quercetin | ABCA1 |
| 174 | Emodin | AMACR | 497 | Nobiletin | CD36 | 820 | Quercetin | PGK1 |
| 175 | Emodin | CPOX | 498 | Nobiletin | PIK3CG | 821 | Quercetin | EIF2AK3 |
| 176 | Emodin | PPARA | 499 | Nobiletin | CREB1 | 822 | Quercetin | BIRC3 |
| 177 | Emodin | SLCO2B1 | 500 | Nobiletin | PTGS2 | 823 | Quercetin | LAT |
| 178 | Emodin | NGFR | 501 | Nobiletin | MMP9 | 824 | Quercetin | BIRC2 |
| 179 | Emodin | LTF | 502 | Nobiletin | MSR1 | 825 | Quercetin | CDC6 |
| 180 | Emodin | BMP6 | 503 | Nobiletin | ABCB1 | 826 | Quercetin | RASSF1 |
| 181 | Emodin | ALOX12 | 504 | Nobiletin | BAX | 827 | Quercetin | E2F1 |
| 182 | Emodin | COX5A | 505 | Nobiletin | BCL2 | 828 | Quercetin | POSTN |
| 183 | Emodin | SLC22A6 | 506 | Nobiletin | MMP1 | 829 | Quercetin | SLC2A10 |
| 184 | Emodin | SULT1E1 | 507 | Nobiletin | CASP9 | 830 | Quercetin | E2F2 |
| 185 | Emodin | CSF2 | 508 | Nobiletin | CYP3A4 | 831 | Quercetin | BNIP3 |
| 186 | Emodin | THBD | 509 | Nobiletin | MBL2 | 832 | Quercetin | GDF15 |
| 187 | Emodin | MAOB | 510 | Nobiletin | MBP | 833 | Quercetin | CLDN2 |
| 188 | Emodin | AKR1C1 | 511 | Nobiletin | CPB2 | 834 | Quercetin | CTRL |
| 189 | Emodin | FABP2 | 512 | Nobiletin | PLA2G4A | 835 | Quercetin | KRAS |
| 190 | Emodin | ASIC1 | 513 | Nobiletin | SP1 | 836 | Quercetin | PRDX5 |
| 191 | Emodin | IFNB1 | 514 | Nobiletin | ADAMTS4 | 837 | Quercetin | IGF1R |
| 192 | Emodin | SLC22A8 | 515 | Nobiletin | LDLR | 838 | Quercetin | PTPN11 |
| 193 | Emodin | CCL4 | 516 | Nobiletin | SCARB1 | 839 | Quercetin | CBL |
| 194 | Emodin | S100A7 | 517 | Nobiletin | CYP19A1 | 840 | Quercetin | NOS1 |
| 195 | Emodin | SLC22A11 | 518 | Nobiletin | MMP7 | 841 | Quercetin | NDUFS3 |
| 196 | Emodin | SLC5A8 | 519 | Nobiletin | OLR1 | 842 | Quercetin | PLA2G1B |
| 197 | Emodin | UGT2B4 | 520 | Nobiletin | GUSB | 843 | Quercetin | STUB1 |
| 198 | Emodin | SLCO1A2 | 521 | Nobiletin | CYP2A6 | 844 | Quercetin | CDKN3 |
| 199 | Emodin | CGB5 | 522 | Nobiletin | CYP1A1 | 845 | Quercetin | COPS6 |
| 200 | Polydatin | PCSK9 | 523 | Nobiletin | HMGCR | 846 | Quercetin | GDE1 |
| 201 | Polydatin | AKT1 | 524 | Nobiletin | SLCO1A2 | 847 | Quercetin | GSTT1 |
| 202 | Polydatin | ICAM1 | 525 | Hesperidin | BAX | 848 | Quercetin | FAS |
| 203 | Salvianolic acid B | MYC | 526 | Hesperidin | GUSB | 849 | Quercetin | GAPDH |
| 204 | Salvianolic acid B | AKT1 | 527 | Hesperidin | AURKB | 850 | Quercetin | CNR1 |
| 205 | Salvianolic acid B | ABCB1 | 528 | Hesperidin | MMP9 | 851 | Quercetin | CYP2E1 |
| 206 | Salvianolic acid B | CASP3 | 529 | Hesperidin | TNF | 852 | Quercetin | THBS1 |
| 207 | Salvianolic acid B | MAPK1 | 530 | Hesperidin | ICAM1 | 853 | Quercetin | ELK1 |
| 208 | Salvianolic acid B | TGFB1 | 531 | Hesperidin | KNG1 | 854 | Quercetin | HBG2 |
| 209 | Salvianolic acid B | PPARA | 532 | Hesperidin | OPRK1 | 855 | Quercetin | ENPP3 |
| 210 | Salvianolic acid B | MAPK14 | 533 | Hesperidin | NFE2L2 | 856 | Quercetin | CRH |
| 211 | Salvianolic acid B | MAPK8 | 534 | Hesperidin | MAFK | 857 | Quercetin | STAT1 |
| 212 | Salvianolic acid B | NOS3 | 535 | Hesperidin | MAPK1 | 858 | Quercetin | SERPINE1 |
| 213 | Salvianolic acid B | CTNNB1 | 536 | Hesperidin | EDN1 | 859 | Quercetin | DHRS4 |
| 214 | Salvianolic acid B | MMP2 | 537 | Hesperidin | MAPK8 | 860 | Quercetin | CDH1 |
| 215 | Salvianolic acid B | MTOR | 538 | Hesperidin | JUN | 861 | Quercetin | NFKBIA |
| 216 | Salvianolic acid B | VCAM1 | 539 | Hesperidin | IGHE | 862 | Quercetin | ADAM9 |
| 217 | Salvianolic acid B | MAPK3 | 540 | Hesperidin | FAM215A | 863 | Quercetin | TCF4 |
| 218 | Salvianolic acid B | HMGCR | 541 | Hesperidin | ALB | 864 | Quercetin | TCF7L2 |
| 219 | Salvianolic acid B | ACE | 542 | Hesperidin | CASP3 | 865 | Quercetin | SERPINB5 |
| 220 | Salvianolic acid B | SERPINE1 | 543 | Hesperidin | GSR | 866 | Quercetin | HSF2 |
| 221 | Salvianolic acid B | ACHE | 544 | Hesperidin | NOS2 | 867 | Quercetin | SELL |
| 222 | Salvianolic acid B | MET | 545 | Hesperidin | SLCO2B1 | 868 | Quercetin | SP3 |
| 223 | Salvianolic acid B | EDN1 | 546 | Hesperidin | MPO | 869 | Quercetin | NUMBL |
| 224 | Salvianolic acid B | CREB1 | 547 | Hesperidin | BCL2 | 870 | Quercetin | MIRLET7C |
| 225 | Salvianolic acid B | MMP1 | 548 | Hesperidin | SLCO1A2 | 871 | Quercetin | CXCR4 |
| 226 | Salvianolic acid B | RB1 | 549 | Hyperoside | AGER | 872 | Quercetin | HGF |
| 227 | Salvianolic acid B | SHC1 | 550 | Hyperoside | CCND1 | 873 | Quercetin | ACE |
| 228 | Salvianolic acid B | NR1I2 | 551 | Hyperoside | ADRB2 | 874 | Quercetin | G6PD |
| 229 | Salvianolic acid B | AKR1B1 | 552 | Hyperoside | MIRLET7A1 | 875 | Quercetin | MUC1 |
| 230 | Salvianolic acid B | ATF4 | 553 | Quercetin | PIR | 876 | Quercetin | XIAP |
| 231 | Salvianolic acid B | SIRT1 | 554 | Quercetin | CASP3 | 877 | Quercetin | IL4 |
| 232 | Salvianolic acid B | GOT1 | 555 | Quercetin | SOD1 | 878 | Quercetin | GLI1 |
| 233 | Salvianolic acid B | HSPA5 | 556 | Quercetin | MAPK1 | 879 | Quercetin | EPCAM |
| 234 | Salvianolic acid B | ATF2 | 557 | Quercetin | HSPA4 | 880 | Quercetin | ACP5 |
| 235 | Salvianolic acid B | AIFM1 | 558 | Quercetin | CAT | 881 | Quercetin | OGG1 |
| 236 | Salvianolic acid B | EIF4EBP1 | 559 | Quercetin | CYP1A1 | 882 | Quercetin | TNFRSF11A |
| 237 | Salvianolic acid B | SMAD3 | 560 | Quercetin | OXA1L | 883 | Quercetin | TRPV6 |
| 238 | Salvianolic acid B | MIR106B | 561 | Quercetin | JUN | 884 | Quercetin | APEX1 |
| 239 | Salvianolic acid B | BMP7 | 562 | Quercetin | MAPK8 | 885 | Quercetin | STS |
| 240 | Salvianolic acid B | EIF2AK3 | 563 | Quercetin | BCL2 | 886 | Quercetin | IQGAP1 |
| 241 | Salvianolic acid B | SIK2 | 564 | Quercetin | PIK3CG | 887 | Quercetin | STMN1 |
| 242 | Salvianolic acid B | PPIA | 565 | Quercetin | BAX | 888 | Quercetin | GSTM1 |
| 243 | Salvianolic acid B | PRMT1 | 566 | Quercetin | UGT1A1 | 889 | Quercetin | RYR3 |
| 244 | Salvianolic acid B | VLDLR | 567 | Quercetin | ABCB1 | 890 | Quercetin | KLRK1 |
| 245 | Salvianolic acid B | CDH5 | 568 | Quercetin | XDH | 891 | Quercetin | PRKCQ |
| 246 | Salvianolic acid B | PHB | 569 | Quercetin | CBR1 | 892 | Quercetin | MPO |
| 247 | Salvianolic acid B | ATF6 | 570 | Quercetin | MAPK14 | 893 | Quercetin | MET |
| 248 | Salvianolic acid B | TAGLN2 | 571 | Quercetin | PARP1 | 894 | Quercetin | ATM |
| 249 | Salvianolic acid B | DUSP6 | 572 | Quercetin | GUSB | 895 | Quercetin | IL2RA |
| 250 | Salvianolic acid B | ERP29 | 573 | Quercetin | NOS2 | 896 | Quercetin | AURKA |
| 251 | Tanshinone II-A | TP53 | 574 | Quercetin | TYR | 897 | Quercetin | AURKB |
| 252 | Tanshinone II-A | TNF | 575 | Quercetin | ANXA5 | 898 | Quercetin | TFRC |
| 253 | Tanshinone II-A | IFNG | 576 | Quercetin | SIRT1 | 899 | Quercetin | F2R |
| 254 | Tanshinone II-A | TGFB1 | 577 | Quercetin | HSPA1A | 900 | Quercetin | SERPINH1 |
| 255 | Tanshinone II-A | BCL2 | 578 | Quercetin | BIRC5 | 901 | Quercetin | TWIST1 |
| 256 | Tanshinone II-A | MTOR | 579 | Quercetin | HSPA8 | 902 | Quercetin | TXN |
| 257 | Tanshinone II-A | MAPK1 | 580 | Quercetin | CCND1 | 903 | Quercetin | SNAI2 |
| 258 | Tanshinone II-A | EGFR | 581 | Quercetin | ABCC2 | 904 | Quercetin | H2AFX |
| 259 | Tanshinone II-A | AKT1 | 582 | Quercetin | CYCS | 905 | Quercetin | THBD |
| 260 | Tanshinone II-A | VEGFA | 583 | Quercetin | HIF1A | 906 | Quercetin | UGDH |
| 261 | Tanshinone II-A | NOS3 | 584 | Quercetin | HSPB1 | 907 | Quercetin | EREG |
| 262 | Tanshinone II-A | BAX | 585 | Quercetin | CYP3A4 | 908 | Quercetin | ALOXE3 |
| 263 | Tanshinone II-A | MAPK14 | 586 | Quercetin | SULT1A3 | 909 | Quercetin | GABPA |
| 264 | Tanshinone II-A | MYC | 587 | Quercetin | COMT | 910 | Quercetin | CTSB |
| 265 | Tanshinone II-A | ALB | 588 | Quercetin | AR | 911 | Quercetin | CDC25C |
| 266 | Tanshinone II-A | LDLR | 589 | Quercetin | SP1 | 912 | Quercetin | NLRP3 |
| 267 | Tanshinone II-A | XDH | 590 | Quercetin | UGT1A9 | 913 | Quercetin | ALOX15 |
| 268 | Tanshinone II-A | IL6 | 591 | Quercetin | NQO1 | 914 | Quercetin | CYB5A |
| 269 | Tanshinone II-A | SOD1 | 592 | Quercetin | HSP90AA1 | 915 | Quercetin | CYBRD1 |
| 270 | Tanshinone II-A | EGF | 593 | Quercetin | CTNNB1 | 916 | Quercetin | TXNIP |
| 271 | Tanshinone II-A | IL1B | 594 | Quercetin | AKT1 | 917 | Quercetin | IFNA1 |
| 272 | Tanshinone II-A | PTGS2 | 595 | Quercetin | GBA3 | 918 | Quercetin | ATP5F1E |
| 273 | Tanshinone II-A | FAS | 596 | Quercetin | HMOX1 | 919 | Quercetin | MT-CYB |
| 274 | Tanshinone II-A | HIF1A | 597 | Quercetin | ABCC1 | 920 | Quercetin | MIR146A |
| 275 | Tanshinone II-A | ERBB2 | 598 | Quercetin | ARSH | 921 | Quercetin | FLT4 |
| 276 | Tanshinone II-A | CTNNB1 | 599 | Quercetin | CYP1A2 | 922 | Quercetin | IRF1 |
| 277 | Tanshinone II-A | NFE2L2 | 600 | Quercetin | IL1B | 923 | Quercetin | PPARGC1A |
| 278 | Tanshinone II-A | NR3C1 | 601 | Quercetin | PIK3CA | 924 | Quercetin | VEGFC |
| 279 | Tanshinone II-A | MAPK8 | 602 | Quercetin | NFE2L2 | 925 | Quercetin | SELP |
| 280 | Tanshinone II-A | PIK3CG | 603 | Quercetin | GSR | 926 | Quercetin | CS |
| 281 | Tanshinone II-A | TYR | 604 | Quercetin | CASP9 | 927 | Quercetin | CSF3 |
| 282 | Tanshinone II-A | AR | 605 | Quercetin | BCL2L1 | 928 | Quercetin | TOP1MT |
| 283 | Tanshinone II-A | CCL2 | 606 | Quercetin | HSF1 | 929 | Quercetin | MTOR |
| 284 | Tanshinone II-A | ABCB1 | 607 | Quercetin | CCNB1 | 930 | Quercetin | BRAF |
| 285 | Tanshinone II-A | NR1H2 | 608 | Quercetin | SULT1A1 | 931 | Quercetin | JAK2 |
| 286 | Tanshinone II-A | JUN | 609 | Quercetin | VCAM1 | 932 | Quercetin | MAPT |
| 287 | Tanshinone II-A | ERCC1 | 610 | Quercetin | CYP2C8 | 933 | Quercetin | HPRT1 |
| 288 | Tanshinone II-A | SP1 | 611 | Quercetin | ESR1 | 934 | Quercetin | HDAC9 |
| 289 | Tanshinone II-A | PLA2G2A | 612 | Quercetin | ALB | 935 | Quercetin | HMGCR |
| 290 | Tanshinone II-A | ICAM1 | 613 | Quercetin | IL10 | 936 | Quercetin | G6PC |
| 291 | Tanshinone II-A | MMP9 | 614 | Quercetin | GSTP1 | 937 | Quercetin | SREBF2 |
| 292 | Tanshinone II-A | SIRT1 | 615 | Quercetin | MAPK3 | 938 | Quercetin | CXCL1 |
| 293 | Tanshinone II-A | CASP3 | 616 | Quercetin | ESR2 | 939 | Quercetin | PSMC6 |
| 294 | Tanshinone II-A | FOS | 617 | Quercetin | CASP8 | 940 | Quercetin | USF2 |
| 295 | Tanshinone II-A | CYP1A2 | 618 | Quercetin | CYP2A6 | 941 | Quercetin | ODC1 |
| 296 | Tanshinone II-A | MMP2 | 619 | Quercetin | HSPB2 | 942 | Quercetin | NKX3-1 |
| 297 | Tanshinone II-A | MAPK3 | 620 | Quercetin | GP6 | 943 | Quercetin | SULT2B1 |
| 298 | Tanshinone II-A | ATM | 621 | Quercetin | PTK2B | 944 | Quercetin | IGFBP3 |
| 299 | Tanshinone II-A | TLR4 | 622 | Quercetin | CSN1S1 | 945 | Quercetin | CSNK2A2 |
| 300 | Tanshinone II-A | NFKB1 | 623 | Quercetin | ERBB3 | 946 | Quercetin | CYP19A1 |
| 301 | Tanshinone II-A | RELA | 624 | Quercetin | BAD | 947 | Quercetin | SLC47A1 |
| 302 | Tanshinone II-A | F3 | 625 | Quercetin | SLC5A1 | 948 | Quercetin | PLA2G6 |
| 303 | Tanshinone II-A | EP300 | 626 | Quercetin | AHR | 949 | Quercetin | SOD2 |
| 304 | Tanshinone II-A | RPS6KB1 | 627 | Quercetin | TGFA | 950 | Quercetin | LTF |
| 305 | Tanshinone II-A | VCAM1 | 628 | Quercetin | UGT1A | 951 | Quercetin | VHL |
| 306 | Tanshinone II-A | SRC | 629 | Quercetin | RAF1 | 952 | Quercetin | PRKCD |
| 307 | Tanshinone II-A | TNFRSF1A | 630 | Quercetin | CYP1B1 | 953 | Quercetin | PRKCG |
| 308 | Tanshinone II-A | HMGB1 | 631 | Quercetin | AKR1C1 | 954 | Quercetin | PRKCE |
| 309 | Tanshinone II-A | CYBB | 632 | Quercetin | CDK4 | 955 | Quercetin | PRKCH |
| 310 | Tanshinone II-A | CD40 | 633 | Quercetin | STAT3 | 956 | Quercetin | PRKCI |
| 311 | Tanshinone II-A | PCNA | 634 | Quercetin | NR3C1 | 957 | Quercetin | PRKCZ |
| 312 | Tanshinone II-A | IKBKB | 635 | Quercetin | PON1 | 958 | Quercetin | PRKCB |
| 313 | Tanshinone II-A | SELE | 636 | Quercetin | LYN | 959 | Quercetin | PRKD1 |
| 314 | Tanshinone II-A | BCL2L1 | 637 | Quercetin | PIKFYVE | 960 | Quercetin | PKN1 |
| 315 | Tanshinone II-A | CYP3A4 | 638 | Quercetin | GSTA1 | 961 | Quercetin | PRKD2 |
| 316 | Tanshinone II-A | CDKN1A | 639 | Quercetin | GGT1 | 962 | Quercetin | PRKD3 |
| 317 | Tanshinone II-A | CYP1A1 | 640 | Quercetin | LPO | 963 | Quercetin | PKN2 |
| 318 | Tanshinone II-A | TIMP1 | 641 | Quercetin | PLA2G2A | 964 | Quercetin | PKN3 |
| 319 | Tanshinone II-A | MMP13 | 642 | Quercetin | IL2 | 965 | Quercetin | PIK3R6 |
| 320 | Tanshinone II-A | NCF1 | 643 | Quercetin | HDC | 966 | Isoquercitrin | ETS1 |
| 321 | Tanshinone II-A | PCSK9 | 644 | Quercetin | CDK1 | 967 | Isoquercitrin | ATF6 |
| 322 | Tanshinone II-A | TUBB | 645 | Quercetin | UGT1A6 | 968 | Isoquercitrin | CTNNB1 |
| 323 | Tanshinone II-A | TLR2 | 646 | Quercetin | HSPG2 | 969 | Isoquercitrin | NOS2 |

Table S4 The information of 231 targets related to hyperlipidemia.

| NO. | Disease | Targets | Targets Full Name | Score |
| --- | --- | --- | --- | --- |
| 1 | Hyperlipidemia | ABCA1 | ATP binding cassette subfamily A member 1 | 0.22 |
| 2 | Hyperlipidemia | ABCB1 | ATP binding cassette subfamily B member 1 | 0.2 |
| 3 | Hyperlipidemia | ABCG5 | ATP binding cassette subfamily G member 5 | 0.31 |
| 4 | Hyperlipidemia | ABCG8 | ATP binding cassette subfamily G member 8 | 0.1 |
| 5 | Hyperlipidemia | ACADSB | acyl-CoA dehydrogenase short/branched chain | 0.01 |
| 6 | Hyperlipidemia | ACAT1 | acetyl-CoA acetyltransferase 1 | 0.02 |
| 7 | Hyperlipidemia | ACE | angiotensin I converting enzyme | 0.01 |
| 8 | Hyperlipidemia | ACHE | acetylcholinesterase (Cartwright blood group) | 0.01 |
| 9 | Hyperlipidemia | ACSL3 | acyl-CoA synthetase long chain family member 3 | 0.01 |
| 10 | Hyperlipidemia | ACSL5 | acyl-CoA synthetase long chain family member 5 | 0.01 |
| 11 | Hyperlipidemia | ACTN4 | actinin alpha 4 | 0.1 |
| 12 | Hyperlipidemia | ADAMTS7 | ADAM metallopeptidase with thrombospondin type 1 motif 7 | 0.01 |
| 13 | Hyperlipidemia | ADIPOQ | adiponectin, C1Q and collagen domain containing | 0.21 |
| 14 | Hyperlipidemia | ADM | adrenomedullin | 0.01 |
| 15 | Hyperlipidemia | ADRB2 | adrenoceptor beta 2 | 0.31 |
| 16 | Hyperlipidemia | ADRB3 | adrenoceptor beta 3 | 0.31 |
| 17 | Hyperlipidemia | AGL | amylo-alpha-1, 6-glucosidase, 4-alpha-glucanotransferase | 0.1 |
| 18 | Hyperlipidemia | AHR | aryl hydrocarbon receptor | 0.01 |
| 19 | Hyperlipidemia | AKR1B1 | aldo-keto reductase family 1 member B | 0.01 |
| 20 | Hyperlipidemia | ALB | albumin | 0.31 |
| 21 | Hyperlipidemia | ALOX5 | arachidonate 5-lipoxygenase | 0.01 |
| 22 | Hyperlipidemia | ANGPTL3 | angiopoietin like 3 | 0.01 |
| 23 | Hyperlipidemia | ANGPTL4 | angiopoietin like 4 | 0.01 |
| 24 | Hyperlipidemia | APC | APC, WNT signaling pathway regulator | 0.31 |
| 25 | Hyperlipidemia | APOA1 | apolipoprotein A1 | 0.04 |
| 26 | Hyperlipidemia | APOA2 | apolipoprotein A2 | 0.01 |
| 27 | Hyperlipidemia | APOA4 | apolipoprotein A4 | 0.01 |
| 28 | Hyperlipidemia | APOA5 | apolipoprotein A5 | 0.06 |
| 29 | Hyperlipidemia | APOB | apolipoprotein B | 0.5 |
| 30 | Hyperlipidemia | APOBEC1 | apolipoprotein B mRNA editing enzyme catalytic subunit 1 | 0.2 |
| 31 | Hyperlipidemia | APOC1 | apolipoprotein C1 | 0.05 |
| 32 | Hyperlipidemia | APOC2 | apolipoprotein C2 | 0.01 |
| 33 | Hyperlipidemia | APOC3 | apolipoprotein C3 | 0.18 |
| 34 | Hyperlipidemia | APOE | apolipoprotein E | 0.6 |
| 35 | Hyperlipidemia | APOF | apolipoprotein F | 0.01 |
| 36 | Hyperlipidemia | APRT | adenine phosphoribosyltransferase | 0.03 |
| 37 | Hyperlipidemia | ARSA | arylsulfatase A | 0.01 |
| 38 | Hyperlipidemia | AS3MT | arsenite methyltransferase | 0.01 |
| 39 | Hyperlipidemia | ASRGL1 | asparaginase like 1 | 0.02 |
| 40 | Hyperlipidemia | BAX | BCL2 associated X, apoptosis regulator | 0.01 |
| 41 | Hyperlipidemia | BCHE | butyrylcholinesterase | 0.01 |
| 42 | Hyperlipidemia | BUD13 | BUD13 homolog | 0.01 |
| 43 | Hyperlipidemia | C3 | complement C3 | 0.01 |
| 44 | Hyperlipidemia | C5AR2 | complement component 5a receptor 2 | 0.01 |
| 45 | Hyperlipidemia | CAD | carbamoyl-phosphate synthetase 2, aspartate transcarbamylase, and dihydroorotase | 0.01 |
| 46 | Hyperlipidemia | CASP1 | caspase 1 | 0.01 |
| 47 | Hyperlipidemia | CCL2 | C-C motif chemokine ligand 2 | 0.01 |
| 48 | Hyperlipidemia | CCL5 | C-C motif chemokine ligand 5 | 0.01 |
| 49 | Hyperlipidemia | CCR2 | C-C motif chemokine receptor 2 | 0.01 |
| 50 | Hyperlipidemia | CCR5 | C-C motif chemokine receptor 5 (gene/pseudogene) | 0.01 |
| 51 | Hyperlipidemia | CCR7 | C-C motif chemokine receptor 7 | 0.01 |
| 52 | Hyperlipidemia | CD36 | CD36 molecule | 0.04 |
| 53 | Hyperlipidemia | CD40 | CD40 molecule | 0.01 |
| 54 | Hyperlipidemia | CD68 | CD68 molecule | 0.01 |
| 55 | Hyperlipidemia | CDC73 | cell division cycle 73 | 0.01 |
| 56 | Hyperlipidemia | CDCP1 | CUB domain containing protein 1 | 0.01 |
| 57 | Hyperlipidemia | CDH13 | cadherin 13 | 0.01 |
| 58 | Hyperlipidemia | CEL | carboxyl ester lipase | 0.01 |
| 59 | Hyperlipidemia | CETP | cholesteryl ester transfer protein | 0.44 |
| 60 | Hyperlipidemia | CHDH | choline dehydrogenase | 0.01 |
| 61 | Hyperlipidemia | CHIT1 | chitinase 1 | 0.01 |
| 62 | Hyperlipidemia | CNBP | CCHC-type zinc finger nucleic acid binding protein | 0.02 |
| 63 | Hyperlipidemia | CNR1 | cannabinoid receptor 1 | 0.01 |
| 64 | Hyperlipidemia | CP | ceruloplasmin | 0.01 |
| 65 | Hyperlipidemia | CPE | carboxypeptidase E | 0.01 |
| 66 | Hyperlipidemia | CRP | C-reactive protein | 0.2 |
| 67 | Hyperlipidemia | CYP19A1 | cytochrome P450 family 19 subfamily A member 1 | 0.1 |
| 68 | Hyperlipidemia | CYP27A1 | cytochrome P450 family 27 subfamily A member 1 | 0.01 |
| 69 | Hyperlipidemia | CYP2C19 | cytochrome P450 family 2 subfamily C member 19 | 0.01 |
| 70 | Hyperlipidemia | CYP2C9 | cytochrome P450 family 2 subfamily C member 9 | 0.01 |
| 71 | Hyperlipidemia | CYP2D6 | cytochrome P450 family 2 subfamily D member 6 | 0.01 |
| 72 | Hyperlipidemia | CYP3A51P | cytochrome P450 family 3 subfamily A member 51, pseudogene | 0.01 |
| 73 | Hyperlipidemia | CYP7A1 | cytochrome P450 family 7 subfamily A member 1 | 0.01 |
| 74 | Hyperlipidemia | DCAF17 | DDB1 and CUL4 associated factor 17 | 0.1 |
| 75 | Hyperlipidemia | DECR1 | 2,4-dienoyl-CoA reductase 1 | 0.01 |
| 76 | Hyperlipidemia | DEFA3 | defensin alpha 3 | 0.01 |
| 77 | Hyperlipidemia | DGAT1 | diacylglycerol O-acyltransferase 1 | 0.1 |
| 78 | Hyperlipidemia | DISC1 | DISC1 scaffold protein | 0.01 |
| 79 | Hyperlipidemia | DOCK7 | dedicator of cytokinesis 7 | 0.01 |
| 80 | Hyperlipidemia | EEF1A2 | eukaryotic translation elongation factor 1 alpha 2 | 0.05 |
| 81 | Hyperlipidemia | ELF3 | E74 like ETS transcription factor 3 | 0.02 |
| 82 | Hyperlipidemia | EPHB2 | EPH receptor B2 | 0.01 |
| 83 | Hyperlipidemia | ESRRA | estrogen related receptor alpha | 0.01 |
| 84 | Hyperlipidemia | F2 | coagulation factor II, thrombin | 0.01 |
| 85 | Hyperlipidemia | F5 | coagulation factor V | 0.01 |
| 86 | Hyperlipidemia | F7 | coagulation factor VII | 0.22 |
| 87 | Hyperlipidemia | FABP2 | fatty acid binding protein 2 | 0.01 |
| 88 | Hyperlipidemia | FGF21 | fibroblast growth factor 21 | 0.01 |
| 89 | Hyperlipidemia | FSD1 | fibronectin type III and SPRY domain containing 1 | 0.01 |
| 90 | Hyperlipidemia | FSD1L | fibronectin type III and SPRY domain containing 1 like | 0.01 |
| 91 | Hyperlipidemia | G6PC | glucose-6-phosphatase catalytic subunit | 0.1 |
| 92 | Hyperlipidemia | GALNT2 | polypeptide N-acetylgalactosaminyltransferase 2 | 0.01 |
| 93 | Hyperlipidemia | GCG | glucagon | 0.3 |
| 94 | Hyperlipidemia | GFPT1 | glutamine--fructose-6-phosphate transaminase 1 | 0.2 |
| 95 | Hyperlipidemia | GGT1 | gamma-glutamyltransferase 1 | 0.01 |
| 96 | Hyperlipidemia | GGT2 | gamma-glutamyltransferase 2 | 0.01 |
| 97 | Hyperlipidemia | GGTLC3 | gamma-glutamyltransferase light chain family member 3 | 0.01 |
| 98 | Hyperlipidemia | GGTLC4P | gamma-glutamyltransferase light chain 4 pseudogene | 0.01 |
| 99 | Hyperlipidemia | GGTLC5P | gamma-glutamyltransferase light chain 5 pseudogene | 0.01 |
| 100 | Hyperlipidemia | GH1 | growth hormone 1 | 0.01 |
| 101 | Hyperlipidemia | GLA | galactosidase alpha | 0.1 |
| 102 | Hyperlipidemia | GLB1 | galactosidase beta 1 | 0.01 |
| 103 | Hyperlipidemia | GREB1 | growth regulating estrogen receptor binding 1 | 0.01 |
| 104 | Hyperlipidemia | HDL3 | Huntington-like neurodegenerative disorder 2 | 0.01 |
| 105 | Hyperlipidemia | HELZ2 | helicase with zinc finger 2 | 0.01 |
| 106 | Hyperlipidemia | HFE | homeostatic iron regulator | 0.01 |
| 107 | Hyperlipidemia | HIF1A | hypoxia inducible factor 1 subunit alpha | 0.02 |
| 108 | Hyperlipidemia | HMGCR | 3-hydroxy-3-methylglutaryl-CoA reductase | 0.01 |
| 109 | Hyperlipidemia | HP | haptoglobin | 0.01 |
| 110 | Hyperlipidemia | HSD11B1 | hydroxysteroid 11-beta dehydrogenase 1 | 0.2 |
| 111 | Hyperlipidemia | HSPG2 | heparan sulfate proteoglycan 2 | 0.01 |
| 112 | Hyperlipidemia | ID2 | inhibitor of DNA binding 2 | 0.01 |
| 113 | Hyperlipidemia | IL17A | interleukin 17A | 0.01 |
| 114 | Hyperlipidemia | IL1B | interleukin 1 beta | 0.01 |
| 115 | Hyperlipidemia | IL6 | interleukin 6 | 0.01 |
| 116 | Hyperlipidemia | INPP5F | inositol polyphosphate-5-phosphatase F | 0.01 |
| 117 | Hyperlipidemia | IRF6 | interferon regulatory factor 6 | 0.01 |
| 118 | Hyperlipidemia | IRS1 | insulin receptor substrate 1 | 0.3 |
| 119 | Hyperlipidemia | JPH3 | junctophilin 3 | 0.01 |
| 120 | Hyperlipidemia | KIDINS220 | kinase D interacting substrate 220 | 0.01 |
| 121 | Hyperlipidemia | KL | klotho | 0.2 |
| 122 | Hyperlipidemia | KLRC4-KLRK1 | KLRC4-KLRK1 readthrough | 0.01 |
| 123 | Hyperlipidemia | KLRK1 | killer cell lectin like receptor K1 | 0.01 |
| 124 | Hyperlipidemia | LCAT | lecithin-cholesterol acyltransferase | 0.21 |
| 125 | Hyperlipidemia | LDLR | low density lipoprotein receptor | 0.5 |
| 126 | Hyperlipidemia | LDLRAP1 | low density lipoprotein receptor adaptor protein 1 | 0.1 |
| 127 | Hyperlipidemia | LEP | leptin | 0.23 |
| 128 | Hyperlipidemia | LEPR | leptin receptor | 0.31 |
| 129 | Hyperlipidemia | LIPA | lipase A, lysosomal acid type | 0.01 |
| 130 | Hyperlipidemia | LIPC | lipase C, hepatic type | 0.5 |
| 131 | Hyperlipidemia | LMNA | lamin A/C | 0.1 |
| 132 | Hyperlipidemia | LMX1B | LIM homeobox transcription factor 1 beta | 0.3 |
| 133 | Hyperlipidemia | LPA | lipoprotein(a) | 0.03 |
| 134 | Hyperlipidemia | LPAL2 | lipoprotein(a) like 2, pseudogene | 0.01 |
| 135 | Hyperlipidemia | LPL | lipoprotein lipase | 0.4 |
| 136 | Hyperlipidemia | LRP1 | LDL receptor related protein 1 | 0.01 |
| 137 | Hyperlipidemia | LYZ | lysozyme | 0.01 |
| 138 | Hyperlipidemia | MAGEE1 | MAGE family member E1 | 0.01 |
| 139 | Hyperlipidemia | MAPK1 | mitogen-activated protein kinase 1 | 0.01 |
| 140 | Hyperlipidemia | MIR107 | microRNA 107 | 0.01 |
| 141 | Hyperlipidemia | MIR126 | microRNA 126 | 0.01 |
| 142 | Hyperlipidemia | MIR143 | microRNA 143 | 0.01 |
| 143 | Hyperlipidemia | MIR210 | microRNA 210 | 0.01 |
| 144 | Hyperlipidemia | MIR24-1 | microRNA 24-1 | 0.01 |
| 145 | Hyperlipidemia | MIR27B | microRNA 27b | 0.02 |
| 146 | Hyperlipidemia | MIR29A | microRNA 29a | 0.01 |
| 147 | Hyperlipidemia | MIR30C1 | microRNA 30c-1 | 0.02 |
| 148 | Hyperlipidemia | MIR30C2 | microRNA 30c-2 | 0.02 |
| 149 | Hyperlipidemia | MIR33A | microRNA 33a | 0.01 |
| 150 | Hyperlipidemia | MMP14 | matrix metallopeptidase 14 | 0.01 |
| 151 | Hyperlipidemia | MMP3 | matrix metallopeptidase 3 | 0.01 |
| 152 | Hyperlipidemia | MTTP | microsomal triglyceride transfer protein | 0.24 |
| 153 | Hyperlipidemia | MVP | major vault protein | 0.01 |
| 154 | Hyperlipidemia | MYLK | myosin light chain kinase | 0.01 |
| 155 | Hyperlipidemia | MYO5A | myosin VA | 0.1 |
| 156 | Hyperlipidemia | NAMPT | nicotinamide phosphoribosyltransferase | 0.02 |
| 157 | Hyperlipidemia | NANOS3 | nanos C2HC-type zinc finger 3 | 0.01 |
| 158 | Hyperlipidemia | NEDD4 | neural precursor cell expressed, developmentally down-regulated 4, E3 ubiquitin protein ligase | 0.01 |
| 159 | Hyperlipidemia | NEIL1 | nei like DNA glycosylase 1 | 0.3 |
| 160 | Hyperlipidemia | NOS3 | nitric oxide synthase 3 | 0.52 |
| 161 | Hyperlipidemia | NOV | nephroblastoma overexpressed | 0.01 |
| 162 | Hyperlipidemia | NPC1L1 | NPC1 like intracellular cholesterol transporter 1 | 0.01 |
| 163 | Hyperlipidemia | NPHS1 | NPHS1, nephrin | 0.1 |
| 164 | Hyperlipidemia | NPHS2 | NPHS2, podocin | 0.1 |
| 165 | Hyperlipidemia | NR1H4 | nuclear receptor subfamily 1 group H member 4 | 0.02 |
| 166 | Hyperlipidemia | NR1I2 | nuclear receptor subfamily 1 group I member 2 | 0.01 |
| 167 | Hyperlipidemia | NT5E | 5'-nucleotidase ecto | 0.01 |
| 168 | Hyperlipidemia | OLR1 | oxidized low density lipoprotein receptor 1 | 0.01 |
| 169 | Hyperlipidemia | OSM | oncostatin M | 0.01 |
| 170 | Hyperlipidemia | PAEP | progestagen associated endometrial protein | 0.01 |
| 171 | Hyperlipidemia | PCSK9 | proprotein convertase subtilisin/kexin type 9 | 0.14 |
| 172 | Hyperlipidemia | PIK3C2G | phosphatidylinositol-4-phosphate 3-kinase catalytic subunit type 2 gamma | 0.01 |
| 173 | Hyperlipidemia | PLA2G1B | phospholipase A2 group IB | 0.01 |
| 174 | Hyperlipidemia | PLA2G7 | phospholipase A2 group VII | 0.01 |
| 175 | Hyperlipidemia | PLB1 | phospholipase B1 | 0.01 |
| 176 | Hyperlipidemia | PLG | plasminogen | 0.02 |
| 177 | Hyperlipidemia | PLXNA1 | plexin A1 | 0.01 |
| 178 | Hyperlipidemia | PON1 | paraoxonase 1 | 0.02 |
| 179 | Hyperlipidemia | PPARA | peroxisome proliferator activated receptor alpha | 0.36 |
| 180 | Hyperlipidemia | PPARG | peroxisome proliferator activated receptor gamma | 0.05 |
| 181 | Hyperlipidemia | PPARGC1B | PPARG coactivator 1 beta | 0.3 |
| 182 | Hyperlipidemia | PPBP | pro-platelet basic protein | 0.01 |
| 183 | Hyperlipidemia | PRH1 | proline rich protein HaeIII subfamily 1 | 0.01 |
| 184 | Hyperlipidemia | PRH2 | proline rich protein HaeIII subfamily 2 | 0.01 |
| 185 | Hyperlipidemia | PTGS1 | prostaglandin-endoperoxide synthase 1 | 0.01 |
| 186 | Hyperlipidemia | PTH | parathyroid hormone | 0.01 |
| 187 | Hyperlipidemia | PTPRA | protein tyrosine phosphatase, receptor type A | 0.01 |
| 188 | Hyperlipidemia | PYGL | glycogen phosphorylase L | 0.1 |
| 189 | Hyperlipidemia | RAB27A | RAB27A, member RAS oncogene family | 0.1 |
| 190 | Hyperlipidemia | REN | renin | 0.01 |
| 191 | Hyperlipidemia | RGN | regucalcin | 0.2 |
| 192 | Hyperlipidemia | RPL10 | ribosomal protein L10 | 0.01 |
| 193 | Hyperlipidemia | RPSA | ribosomal protein SA | 0.01 |
| 194 | Hyperlipidemia | SAA1 | serum amyloid A1 | 0.01 |
| 195 | Hyperlipidemia | SAA3P | serum amyloid A3, pseudogene | 0.01 |
| 196 | Hyperlipidemia | SDC1 | syndecan 1 | 0.2 |
| 197 | Hyperlipidemia | SDC2 | syndecan 2 | 0.01 |
| 198 | Hyperlipidemia | SERPINA13P | serpin family A member 13, pseudogene | 0.01 |
| 199 | Hyperlipidemia | SERPINB2 | serpin family B member 2 | 0.01 |
| 200 | Hyperlipidemia | SERPINC1 | serpin family C member 1 | 0.2 |
| 201 | Hyperlipidemia | SERPIND1 | serpin family D member 1 | 0.01 |
| 202 | Hyperlipidemia | SERPINE1 | serpin family E member 1 | 0.02 |
| 203 | Hyperlipidemia | SERPINF2 | serpin family F member 2 | 0.2 |
| 204 | Hyperlipidemia | SHC1 | SHC adaptor protein 1 | 0.2 |
| 205 | Hyperlipidemia | SLC27A1 | solute carrier family 27 member 1 | 0.2 |
| 206 | Hyperlipidemia | SLC2A4 | solute carrier family 2 member 4 | 0.02 |
| 207 | Hyperlipidemia | SLC37A4 | solute carrier family 37 member 4 | 0.1 |
| 208 | Hyperlipidemia | SLCO1B1 | solute carrier organic anion transporter family member 1B1 | 0.03 |
| 209 | Hyperlipidemia | SMARCD1 | SWI/SNF related, matrix associated, actin dependent regulator of chromatin, subfamily d, member 1 | 0.2 |
| 210 | Hyperlipidemia | SOAT1 | sterol O-acyltransferase 1 | 0.02 |
| 211 | Hyperlipidemia | SOD2 | superoxide dismutase 2 | 0.01 |
| 212 | Hyperlipidemia | SREBF1 | sterol regulatory element binding transcription factor 1 | 0.01 |
| 213 | Hyperlipidemia | SREBF2 | sterol regulatory element binding transcription factor 2 | 0.01 |
| 214 | Hyperlipidemia | TFG | TRK-fused gene | 0.1 |
| 215 | Hyperlipidemia | TGFB1 | transforming growth factor beta 1 | 0.2 |
| 216 | Hyperlipidemia | TLR2 | toll like receptor 2 | 0.01 |
| 217 | Hyperlipidemia | TNF | tumor necrosis factor | 0.04 |
| 218 | Hyperlipidemia | TNFRSF11B | TNF receptor superfamily member 11b | 0.01 |
| 219 | Hyperlipidemia | TNFRSF12A | TNF receptor superfamily member 12A | 0.01 |
| 220 | Hyperlipidemia | TNFRSF25 | TNF receptor superfamily member 25 | 0.01 |
| 221 | Hyperlipidemia | TRH | thyrotropin releasing hormone | 0.01 |
| 222 | Hyperlipidemia | TXNIP | thioredoxin interacting protein | 0.01 |
| 223 | Hyperlipidemia | UCP1 | uncoupling protein 1 | 0.01 |
| 224 | Hyperlipidemia | USF1 | upstream transcription factor 1 | 0.01 |
| 225 | Hyperlipidemia | VCAM1 | vascular cell adhesion molecule 1 | 0.22 |
| 226 | Hyperlipidemia | VEGFA | vascular endothelial growth factor A | 0.01 |
| 227 | Hyperlipidemia | VLDLR | very low density lipoprotein receptor | 0.01 |
| 228 | Hyperlipidemia | WDHD1 | WD repeat and HMG-box DNA binding protein 1 | 0.01 |
| 229 | Hyperlipidemia | YWHAZ | tyrosine 3-monooxygenase/tryptophan 5-monooxygenase activation protein zeta | 0.01 |
| 230 | Hyperlipidemia | ZMPSTE24 | zinc metallopeptidase STE24 | 0.1 |
| 231 | Hyperlipidemia | ZPR1 | ZPR1 zinc finger | 0.01 |

Table S5 The information of 65 potential targets of 10 candidate compounds.

| NO. | Compounds | Targets | NO. | Compounds | Targets |
| --- | --- | --- | --- | --- | --- |
| 1 | Emodin | ABCB1 | 72 | Quercetin | CCL2 |
| 2 | Emodin | AHR | 73 | Quercetin | CD36 |
| 3 | Emodin | AKR1B1 | 74 | Quercetin | CNR1 |
| 4 | Emodin | ALB | 75 | Quercetin | CYP19A1 |
| 5 | Emodin | ALOX5 | 76 | Quercetin | CYP2C9 |
| 6 | Emodin | APOE | 77 | Quercetin | CYP2D6 |
| 7 | Emodin | BAX | 78 | Quercetin | G6PC |
| 8 | Emodin | CASP1 | 79 | Quercetin | GGT1 |
| 9 | Emodin | CCL2 | 80 | Quercetin | HIF1A |
| 10 | Emodin | CD36 | 81 | Quercetin | HMGCR |
| 11 | Emodin | CRP | 82 | Quercetin | HSPG2 |
| 12 | Emodin | CYP2C19 | 83 | Quercetin | IL1B |
| 13 | Emodin | CYP2C9 | 84 | Quercetin | IL6 |
| 14 | Emodin | CYP2D6 | 85 | Quercetin | IRS1 |
| 15 | Emodin | FABP2 | 86 | Quercetin | KLRK1 |
| 16 | Emodin | HIF1A | 87 | Quercetin | LDLR |
| 17 | Emodin | IL1B | 88 | Quercetin | MAPK1 |
| 18 | Emodin | IL6 | 89 | Quercetin | MTTP |
| 19 | Emodin | LEP | 90 | Quercetin | NOS3 |
| 20 | Emodin | LRP1 | 91 | Quercetin | NR1I2 |
| 21 | Emodin | MAPK1 | 92 | Quercetin | OLR1 |
| 22 | Emodin | MMP3 | 93 | Quercetin | PIK3C2G |
| 23 | Emodin | PPARA | 94 | Quercetin | PLA2G1B |
| 24 | Emodin | PPARG | 95 | Quercetin | PON1 |
| 25 | Emodin | PTGS1 | 96 | Quercetin | PPARG |
| 26 | Emodin | TGFB1 | 97 | Quercetin | SERPINE1 |
| 27 | Emodin | TNF | 98 | Quercetin | SLCO1B1 |
| 28 | Emodin | VCAM1 | 99 | Quercetin | SOD2 |
| 29 | Emodin | VEGFA | 100 | Quercetin | SREBF2 |
| 30 | Hesperidin | ALB | 101 | Quercetin | TGFB1 |
| 31 | Hesperidin | BAX | 102 | Quercetin | TNF |
| 32 | Hesperidin | MAPK1 | 103 | Quercetin | TXNIP |
| 33 | Hesperidin | TNF | 104 | Quercetin | VCAM1 |
| 34 | Hyperoside | ADRB2 | 105 | Quercetin | VEGFA |
| 35 | Naringenin | ABCB1 | 106 | Salvianolic acid B | ABCB1 |
| 36 | Naringenin | AHR | 107 | Salvianolic acid B | ACE |
| 37 | Naringenin | APOB | 108 | Salvianolic acid B | ACHE |
| 38 | Naringenin | BAX | 109 | Salvianolic acid B | AKR1B1 |
| 39 | Naringenin | CYP19A1 | 110 | Salvianolic acid B | HMGCR |
| 40 | Naringenin | HMGCR | 111 | Salvianolic acid B | MAPK1 |
| 41 | Naringenin | LDLR | 112 | Salvianolic acid B | NOS3 |
| 42 | Naringenin | MAPK1 | 113 | Salvianolic acid B | NR1I2 |
| 43 | Naringenin | MTTP | 114 | Salvianolic acid B | PPARA |
| 44 | Naringenin | NOS3 | 115 | Salvianolic acid B | SERPINE1 |
| 45 | Naringenin | PPARG | 116 | Salvianolic acid B | SHC1 |
| 46 | Naringenin | SOAT1 | 117 | Salvianolic acid B | TGFB1 |
| 47 | Naringenin | SREBF1 | 118 | Salvianolic acid B | VCAM1 |
| 48 | Naringin | ABCB1 | 119 | Salvianolic acid B | VLDLR |
| 49 | Naringin | CASP1 | 120 | Tanshinone II-A | ABCB1 |
| 50 | Naringin | HMGCR | 121 | Tanshinone II-A | AKR1B1 |
| 51 | Nobiletin | ABCB1 | 122 | Tanshinone II-A | ALB |
| 52 | Nobiletin | BAX | 123 | Tanshinone II-A | BAX |
| 53 | Nobiletin | CD36 | 124 | Tanshinone II-A | CCL2 |
| 54 | Nobiletin | CYP19A1 | 125 | Tanshinone II-A | CCR2 |
| 55 | Nobiletin | HMGCR | 126 | Tanshinone II-A | CD40 |
| 56 | Nobiletin | LDLR | 127 | Tanshinone II-A | HIF1A |
| 57 | Nobiletin | MAPK1 | 128 | Tanshinone II-A | IL1B |
| 58 | Nobiletin | OLR1 | 129 | Tanshinone II-A | IL6 |
| 59 | Polydatin | PCSK9 | 130 | Tanshinone II-A | LDLR |
| 60 | Quercetin | ABCA1 | 131 | Tanshinone II-A | LRP1 |
| 61 | Quercetin | ABCB1 | 132 | Tanshinone II-A | MAPK1 |
| 62 | Quercetin | ACE | 133 | Tanshinone II-A | NAMPT |
| 63 | Quercetin | AHR | 134 | Tanshinone II-A | NOS3 |
| 64 | Quercetin | AKR1B1 | 135 | Tanshinone II-A | NR1I2 |
| 65 | Quercetin | ALB | 136 | Tanshinone II-A | PCSK9 |
| 66 | Quercetin | ALOX5 | 137 | Tanshinone II-A | PTGS1 |
| 67 | Quercetin | APOA1 | 138 | Tanshinone II-A | TGFB1 |
| 68 | Quercetin | APOB | 139 | Tanshinone II-A | TLR2 |
| 69 | Quercetin | APOE | 140 | Tanshinone II-A | TNF |
| 70 | Quercetin | BAX | 141 | Tanshinone II-A | VCAM1 |
| 71 | Quercetin | CASP1 | 142 | Tanshinone II-A | VEGFA |

Table S6 The detail information of the 52 pathways.

| NO. | Pathway | Relevant targets | Cou-nt | P-Value | Notes | Category | References |
| --- | --- | --- | --- | --- | --- | --- | --- |
| 1 | Malaria | VCAM1, IL6, TNF, CD36, CCL2, LRP1, TLR2, IL1B, CD40, TGFB1 | 10 | 6.43E-12 | Other disease: Malaria | Other disease |  |
| 2 | Rheumatoid arthritis | IL6, TNF, CCL2, VEGFA, TLR2, IL1B, MMP3, TGFB1 | 8 | 6.96E-07 | Other disease: Rheumatoid arthritis | Other disease |  |
| 3 | Chagas disease (American trypanosomiasis) | MAPK1, IL6, TNF, CCL2, SERPINE1, TLR2, IL1B, TGFB1 | 8 | 2.17E-06 | Other disease: American trypanosomiasis | Other disease |  |
| 4 | Fat digestion and absorption | APOB, APOA1, CD36, PLA2G1B, ABCA1, MTTP | 6 | 2.90E-06 | Involved in fat digestion and absorption in small intestine | Fat metabolism | (Prakash and Srinivasan, 2012) |
| 5 | NOD-like receptor signaling pathway | MAPK1, IL6, TNF, CCL2, IL1B, CASP1 | 6 | 1.79E-05 | Mainly involved in immune and anti-inflammatory response | Inflammatory and immune | (Shiau et al., 2013) |
| 6 | Non-alcoholic fatty liver disease (NAFLD) | LEP, SREBF1, PPARA, IL6, TNF, IL1B, IRS1, TGFB1 | 8 | 2.57E-05 | Other disease: Non-alcoholic fatty liver disease | Other disease |  |
| 7 | TNF signaling pathway | VCAM1, MAPK1, IL6, TNF, CCL2, IL1B, MMP3 | 7 | 3.55E-05 | Mainly involved in inflammatory response | Inflammatory and immune | (Kirkiles-Smith et al., 2000) |
| 8 | Insulin resistance | SREBF1, PPARA, IL6, TNF, CD36, NOS3, IRS1 | 7 | 3.75E-05 | Mainly involved in glucose intake | Glucose metabolism | (Guo et al., 2013) |
| 9 | African trypanosomiasis | VCAM1, IL6, TNF, APOA1, IL1B | 5 | 3.92E-05 | Other disease: African trypanosomiasis | Other disease |  |
| 10 | Toxoplasmosis | MAPK1, TNF, LDLR, TLR2, ALOX5, CD40, TGFB1 | 7 | 4.16E-05 | Other disease: Toxoplasmosis | Other disease |  |
| 11 | HIF-1 signaling pathway | MAPK1, IL6, HIF1A, VEGFA, SERPINE1, NOS3 | 6 | 2.40E-04 | Mainly involved in angiogensis and inflammation | Angiogensis | (Baatar et al., 2002) |
| 12 | Legionellosis | IL6, TNF, TLR2, IL1B, CASP1 | 5 | 2.77E-04 | Other disease: Legionellosis | Other disease |  |
| 13 | Toll-like receptor signaling pathway | MAPK1, IL6, TNF, TLR2, IL1B, CD40 | 6 | 3.81E-04 | Mainly involved in proinflammatory effects and T cell stimulation | Inflammatory and immune | (Liu et al., 2018) |
| 14 | Arachidonic acid metabolism | CYP2C19, CYP2C9, PTGS1, PLA2G1B, ALOX5 | 5 | 4.44E-04 | Mediate or modulate inflammatory reactions | Inflammatory and immune | (Higgs et al., 1981) |
| 15 | Serotonergic synapse | MAPK1, CYP2C19, CYP2C9, PTGS1, CYP2D6, ALOX5 | 6 | 4.71E-04 | Mainly involved in neuronal excitability and neuroprotectiion | Other metabolism |  |
| 16 | Cytokine-cytokine receptor interaction | LEP, IL6, TNF, CCL2, CCR2, IL1B, CD40, TGFB1 | 8 | 5.06E-04 | Involved in inflammation and immunity | Inflammatory and immune | (Bass et al., 2008) |
| 17 | Inflammatory bowel disease (IBD) | IL6, TNF, TLR2, IL1B, TGFB1 | 5 | 5.33E-04 | Other disease: Inflammatory bowel disease (IBD) | Other disease |  |
| 18 | PPAR signaling pathway | PPARA, APOA1, CD36, OLR1, PPARG | 5 | 6.35E-04 | Lipid metabolism | Lipid metabolism | (Wu and Xu, 2016; Hansjörg et al., 1993) |
| 19 | Adipocytokine signaling pathway | LEP, PPARA, TNF, CD36, IRS1 | 5 | 7.50E-04 | Adipocytokine and involved in energy expenditure and insulin resistance | Fat metabolism | (Maslov et al., 2013) |
| 20 | AMPK signaling pathway | LEP, SREBF1, CD36, HMGCR, PPARG, IRS1 | 6 | 7.54E-04 | mainly Involved in glucose uptake, cell growth, fatty acid synthesis and protein production | Glucose Metabolism | (Maeda et al., 2018) |
| 21 | Leishmaniasis | MAPK1, TNF, TLR2, IL1B, TGFB1 | 5 | 7.91E-04 | Other disease: Leishmaniasis | Other disease |  |
| 22 | Pertussis | MAPK1, IL6, TNF, IL1B, CASP1 | 5 | 9.73E-04 | Other disease: Pertussis | Other disease |  |
| 23 | Proteoglycans in cancer | MAPK1, TNF, HIF1A, VEGFA, TLR2, HSPG2, TGFB1 | 7 | 1.08E-03 | Other disease: Proteoglycans | Other disease |  |
| 24 | Hepatitis B | MAPK1, IL6, TNF, TLR2, HSPG2, TGFB1 | 6 | 1.58E-03 | Other disease: Hepatitis B | Other disease |  |
| 25 | Amoebiasis | IL6, TNF, TLR2, IL1B, TGFB1 | 5 | 3.48E-03 | Other disease: Amoebiasis | Other disease |  |
| 26 | Influenza A | MAPK1, IL6, TNF, CCL2, IL1B, CASP1 | 6 | 3.50E-03 | Other disease: Influenza A | Other disease |  |
| 27 | Tuberculosis | MAPK1, IL6, TNF, TLR2, IL1B, TGFB1 | 6 | 3.77E-03 | Other disease: Tuberculosis | Other disease |  |
| 28 | Low-density lipoprotein (LDL) pathway during atherogenesis | IL6, CCL2, LDLR | 3 | 0.004722 | Other disease: Atherogenesis | Other disease |  |
| 29 | Msp/Ron Receptor Signaling Pathway | TNF, CCL2, IL1B | 3 | 6.53E-03 | Involved in inflammation and immunity | Inflammatory and immune | (Wang et al., 2015) |
| 30 | Renal cell carcinoma | MAPK1, HIF1A, VEGFA, TGFB1 | 4 | 6.93E-03 | Other disease: Renal cell carcinoma | Other disease |  |
| 31 | Osteoclast differentiation | MAPK1, TNF, PPARG, IL1B, TGFB1 | 5 | 7.38E-03 | Involved in cell proliferation, survival and cytoskeletal rearrangement | Other metabolism |  |
| 32 | SREBP control of lipid synthesis | SREBF1, LDLR, SREBF2 | 3 | 8.61E-03 | Involved in lipid synthesis | Lipid metabolism | (Eberle et al., 2004; Giudetti et al., 2013) |
| 33 | Linoleic acid metabolism | CYP2C19, CYP2C9, PLA2G1B | 3 | 1.27E-02 | Linoleic acid metabolism | Other metabolism |  |
| 34 | Salmonella infection | MAPK1, IL6, IL1B, CASP1 | 4 | 1.30E-02 | Other disease: Salmonella infection | Other disease |  |
| 35 | Hematopoietic cell lineage | IL6, TNF, CD36, IL1B | 4 | 1.47E-02 | Involved in cell differentiation and generation | Other metabolism |  |
| 36 | NF-kappa B signaling pathway | VCAM1, TNF, IL1B, CD40 | 4 | 1.47E-02 | Involved in inflammatory, immune and survival | Inflammatory and immune | (Wu et al., 2017) |
| 37 | Graft-versus-host disease | IL6, TNF, IL1B | 3 | 1.63E-02 | Other disease: Graft-versus-host disease | Other disease |  |
| 38 | Alzheimer's disease | MAPK1, TNF, LRP1, APOE, IL1B | 5 | 1.72E-02 | Other disease: Alzheimer's disease | Other disease |  |
| 39 | Prion diseases | MAPK1, IL6, IL1B | 3 | 1.72E-02 | Other disease: Prion diseases | Other disease |  |
| 40 | VEGF, Hypoxia, and Angiogenesis | HIF1A, VEGFA, SHC1, NOS3 | 4 | 1.80E-02 | Involved in hypoxia, and  angiogenesis | Angiogenesis | (Arany et al., 2008) |
| 41 | Signal transduction through IL1R | IL6, TNF, IL1B, TGFB1 | 4 | 2.13E-02 | Involved in inflammatory response | Inflammatory and immune | (Wu et al., 2009) |
| 42 | Herpes simplex infection | IL6, TNF, CCL2, TLR2, IL1B | 5 | 2.28E-02 | Other disease: Herpes | Other disease |  |
| 43 | Nuclear Receptors in Lipid Metabolism and Toxicity | PPARA, CYP2C9, PPARG, ABCA1 | 4 | 0.024963 | Involved in lipid Metabolism | Lipid metabolism | (Beaven, and Tontonoz, 2006) |
| 44 | Intestinal immune network for IgA production | IL6, CD40, TGFB1 | 3 | 3.16E-02 | Involved in immune reaction | Inflammatory and immune | (Hyun-Jeong and Sun-Young, 2015) |
| 45 | Type II diabetes mellitus | MAPK1, TNF, IRS1 | 3 | 3.29E-02 | Type II diabetes | Other disease |  |
| 46 | Cells and Molecules involved in local acute inflammatory response | VCAM1, IL6, TNF | 3 | 3.77E-02 | Inflammatory response | Inflammatory and immune | (Souza et al., 2003) |
| 47 | Hypoxia-Inducible Factor in the Cardiovascular System | HIF1A, VEGFA, NOS3 | 3 | 3.77E-02 | Other disease: Hypoxia | Other disease |  |
| 48 | Regulation of lipolysis in adipocytes | ADRB2, PTGS1, IRS1 | 3 | 4.36E-02 | Involved in lipolysis in adipocytes | Fat metabolism | (Duncan et al., 2007) |
| 49 | Hepatitis C | MAPK1, PPARA, TNF, LDLR | 4 | 4.42E-02 | Other disease: Hepatitis C | Other disease |  |
| 50 | FoxO signaling pathway | MAPK1, IL6, IRS1, TGFB1 | 4 | 4.50E-02 | Mainly involved in apoptosis | Apoptosis | (Yan et al., 2017) |
| 51 | mTOR signaling pathway | MAPK1, TNF, IRS1 | 3 | 4.64E-02 | Mainly involved in apoptosis | Apoptosis | (Kumar et al., 2014) |
| 52 | Insulin signaling pathway | SREBF1, MAPK1, SHC1, IRS1 | 4 | 4.84E-02 | Mainly involved in glucose and lipid homeostasis | Glucose metabolism | (Rice et al., 2011) |

**References**

Arany, Z., Foo, S. Y., Ma, Y., Ruas, J. L., Bommireddy, A., Girnun, G., Cooper, M., Laznik, D., Chinsomboon, J., Rangwala, S. M., Baek, K. H., Rosenzweig A., and Spiegelman, B. M. (2008). Hif-independent regulation of vegf and angiogenesis by the transcriptional coactivator pgc-1[agr]. *Nature.* 45, 1008-1012. doi: 10.1038/nature06613

Baatar, D., Jones, M., Tsugawa, K., Pai, R., Moon, W., Koh, G. Y., Kim, I., Kitano, S., and Tarnawsk A. S. (2002). Esophageal ulceration triggers expression of hypoxia-inducible factor-1 and activates vascular endothelial growth factor gene: implications for angiogenesis and ulcer healing. *Am J Pathol.* 161, 1449-1457. doi: 10.1016/S0002-9440(10)64420-3

Bass, W. T., Buescher, E. S., Hair, P. S., White, L. E., Welch, J. C., and Burke, B. L. (2008). Proinflammatory cytokine-receptor interaction model improves the predictability of cerebral white matter injury in preterm infants. *Am J Perinatol.* 25, 211-218. doi: 10.1055/s-2008-1064931

Beaven, S. W., and Tontonoz, P. (2006). Nuclear receptors in lipid metabolism: targeting the heart of dyslipidemia. *Annu Rev Med.* 57, 313-329. doi: 10.1146/annurev.med.57.121304.131428

Duncan, R. E., Ahmadian, M., Jaworski, K., Sarkadi-Nagy, E., and Sul, H. S. (2007). Regulation of lipolysis in adipocytes. *Annu Rev Nutr.* 27, 79-101. doi: 10.1146/annurev.nutr.27.061406.093734

Eberle, D., Hegarty, B., Bossard, P., Ferré, P., and Foufelle, F. (2004). SREBP transcription factors: master regulators of lipid homeostasis. *Biochimie.* 86, 839-848. doi: 10.1016/j.biochi.2004.09.018

Giudetti, A. M., Damiano, F., Gnoni, G. V., and Siculella, L. (2013). Low level of hydrogen peroxide induces lipid synthesis in brl-3a cells through a cap-independent srebp-1a activation. *Int J* *Biochem Cell B.* 45, 1419-1426. doi: 10.1016/j.biocel.2013.04.004

Guo, X., Yoshitomi, H., Gao, M., Qin, L., Duan, Y., Sun, W., Xu, T., Xie, P., Zhou, J., Huang, L. and Liu, T. (2013). Guava leaf extracts promote glucose metabolism in shrsp.z-leprfa/izm rats by improving insulin resistance in skeletal muscle. *Bmc Complem Altern M.* 13, 52-52. doi: 10.1186/1472-6882-13-52

Hansjörg K., Mahfoudi, A., Dreyer, C., Hihi, A. K., and Wahli, W. (1993). Peroxisome proliferator-activated receptors and lipid metabolism. *Ann Ny Acad Sci.* 684, 157-173. doi: 10.1111/j.1749-6632.1993.tb32279.x

Higgs, G. A., Palmer, R. M. J., Eakins, K. E., and Moncada, S. (1981). Arachidonic acid metabolism as a source of inflammatory mediators and its inhibition as a mechanism of action for anti-inflammatory drugs. *Mol Aspects Med.* 4, 275-301. doi: 10.1016/0098-2997(81)90007-8

Hyun-Jeong, K., and Sun-Young, C. (2015). Regulation of intestinal immune system by dendritic cells*. Immune Netw.* 15, 1-8. doi: 10.4110/in.2015.15.1.1

Kirkiles-Smith, N. C., Tereb, D. A., Kim, R. W., Mcniff, J. M., Schechner, J. S., Lorber, M. I., Pober, J. S., and Tellides, G. (2000). Human TNF can induce nonspecific inflammatory and human immune-mediated microvascular injury of pig skin xenografts in immunodeficient mouse hosts. *J Immunol.* 164, 6601-6609. doi: 10.4049/jimmunol.164.12.6601

Kumar, D., Shankar, S., and Srivastava, R. K. (2014). Rottlerin induces autophagy and apoptosis in prostate cancer stem cells via pi3k/akt/mtor signaling pathway. *Cancer Lett.*343, 179-189. doi: 10.1016/j.canlet.2013.10.003

Liu, Y., Liu, W., Wang, X., Wan, Z., Liu, Y., and Leng, Y. (2018). Dexmedetomidine relieves acute inflammatory visceral pain in rats through the erk pathway, toll-like receptor signaling, and trpv1 channel*. J Mol Neurosci.* 66, 279-290. doi: 10.1007/s12031-018-1172-5

Maeda, A., Shirao, T., Shirasaya, D., Yoshioka, Y., Yamashita, Y., Akagawa, M., and Ashida, H. (2018). Piperine promotes glucose uptake through ros-dependent activation of the camkk/ampk signaling pathway in skeletal muscle. *Mol Nutr Food Res.* 62, e1800086. doi: 10.1002/mnfr.201800086

Maslov, M. A., Morozova, N. G., Solomatina, T. V., Sergeeva, O. A., Cheshkov, D. A., and Serebrennikova, G. A. (2013). Differentially expressed genes in adipocytokine signaling pathway of adipose tissue in pregnancy. *J Diabetes Mellitus.* 3, 86-95. doi: 10.4236/jdm.2013.32013

Prakash, U. N., and Srinivasan, K. (2012). Fat digestion and absorption in spice-pretreated rats. *J Sci Food Agric.* 92, 503-510. doi: 10.1002/jsfa.4597

Rice, S., Pellatt, L. J., Bryan, S. J., Whitehead, S. A., and Mason, H. D. (2011). Action of metformin on the insulin-signaling pathway and on glucose transport in human granulosa cells. *J Clin Endocr Metab.* 96, E427-E435. doi: 10.1210/jc.2010-2060

Shiau, C., Monk, K., Joo, W., and Talbot, W. (2013). An anti-inflammatory nod-like receptor is required for microglia development. *Cell Reports.* 5, 1342-1352. doi: 10.1016/j.celrep.2013.11.004

Souza, D. G., Guabiraba, R., Pinho, V., Bristow, A., Poole, S., and Teixeira, M. M. (2003). Il-1-driven endogenous il-10 production protects against the systemic and local acute inflammatory response following intestinal reperfusion injury. *J Immunol.* 170, 4759-66. doi: 10.4049/jimmunol.170.9.4759

Wang, T., Chen, X., Zhang, W., Xiang, X., and Jia, Q. (2015). Roles of macrophage stimulating protein and tyrosine kinase receptor ron in smoke-induced airway inflammation of rats. *Int J Clin Exp Patho.* 8, 8797-8808.

Wu, C., Ying, H., Bose, S., Miller, R., Medina, L., Santora, L., and Ghayur, T. (2009). Molecular construction and optimization of anti-human il-1alpha/beta dual variable domain immunoglobulin (dvd-ig) molecules. *Mabs.* 1, 339-347. doi: 10.4161/mabs.4.2.19495

Wu, L. Y., Ye, Z. N., Zhou, C. H., Wang, C. X., Xie, G. B., Zhang, X. S., Gao., Y. Y., Zhang Z. H., Zhou, M. L., Zhuang, Z., Liu, J. P., Hang, C. H., and Shi, J. X. (2017). Roles of pannexin-1 channels in inflammatory response through the tlrs/nf-kappa b signaling pathway following experimental subarachnoid hemorrhage in rats. *Front. Mol. Neurosci.* 10, 175. doi: 10.3389/fnmol.2017.00175

Wu, X., and Xu, J. (2016). New role of hispidulin in lipid metabolism: pparα activator.*Lipids.* 51, 1249-1257. doi: 10.1007/s11745-016-4200-7

Yan, P., Tang, S., Zhang, H., Guo, Y., Zeng, Z., and Wen, Q. (2017). Palmitic acid triggers cell apoptosis in rgc-5 retinal ganglion cells through the akt/foxo1 signaling pathway. *Metab Brain Dis.* 32, 1-8. doi: 10.1007/s11011-016-9935-6
