# Supplementary material for: Investigation of the Lipid-Lowering Mechanisms and Active Ingredients of Danhe Granule on Hyperlipidemia Based on Systems Pharmacology
Source: Front Pharmacol. 2020 May 6;11:528. doi: 10.3389/fphar.2020.00528 (PMC7218108; doi:10.3389/fphar.2020.00528)
Supplement: Supplementary file 2 [file DataSheet_2.pdf]

Investigation of the lipid lowering mechanism and active ingredients of Danhe granule on hyperlipidemia based on systems pharmacology

Supplementary Material

Preparation of Danhe Granule (DHG)

Danhe Granule (DHG), a TCM formula, was composed of six herbs, as follows: *Salvia miltiorrhiza Bunge* (Danshen), *Reynoutria japonica* Houtt. (Huzhang), *Crataegus pinnatifida* Bunge (Shanzha), *Citrus × aurantium* L. (Chenpi), *Coix lacryma-jobi var. ma-yuen* (Rom.Caill.) Stapf (Yiyiren), and *Nelumbo nucifera* Gaertn. (Heye). Saliae Mil Tiorrhizae Radix et Rhizoma (Lot No.80270602) [the root and rhizome of *Salvia miltiorrhiza Bunge*, a perennial herbal plant of the *Salvia* L. genus, the *Labiatae* family], Polygoni Cuspidati Rhizoma et Radix (Lot No.80510801) [the root and rhizome of *Reynoutria japonica* Houtt., genus *Reynoutria* Houtt., family *Polygonaceae*], Crataegi Fructus (Lot No.82230701) [the fruit of *Crataegus pinnatifida* Bunge, the short arbor plants of the *Crataegus* L. genus, the *Rosaceae* family], Citri Reticulatae Pericarpium (Lot No. 81450601) [the peel of *Citrus × aurantium* L., genus *Citrus* L., family *Rutaceae*], Coicis Semen (Lot No. 70511101) [the mature kernel of *Coix lacryma-jobi var. ma-yuen* (Rom.Caill.) Stapf, a perennial herbal plant of the *Coix* L. genus, the *Poaceae* family] and Nelumbinis Folium (Lot No. 83720701) [the leaf of *Nelumbo nucifera* Gaertn., genus *Nelumbo* Adans., family *Nelumbonaceae*] were obtained from Beijing San He Co., Ltd. (Beijing, China) and identified by Professor Xueyong Wang (School of Chinese Materia Medica, Beijing University of Chinese Medicine). Danhe Granule (DHG) [production batch No. D1905007] was provided by the hospital preparation room, China-Japan Friendship Hospital (Beijing, China), which was used for pharmacological research in the study. The preparation processes follows: The total herbs including Saliae Mil Tiorrhizae Radix et Rhizoma (1000g), Polygoni Cuspidati Rhizoma et Radix (1000g), Crataegi Fructus (1000g), Citri Reticulatae Pericarpium (1000g), Coicis Semen (3000g) and Nelumbinis Folium (1500g) were mixed and extracted 3 times by refluxing with 12-fold of water (volume/weight) for 1 h each time. The liquid extract was filtered and concentrated to a density of 1.08 (60 ℃). 95% ethanol was added into the concentrated liquid to 60% ethanol concentration, and then it was precipitated at room temperature for 24 hours. The supernatant was condensed and dried to yield 1060 g powder using decompression drying method at 60 ℃.

Quality control of Danhe Granule (DHG) by high performance liquid chromatography (HPLC)

1. Chemicals and materials

HPLC-grade acetonitrile, methanol and formic acid were supplied by Merck (Darmstadt, Germany). HPLC-grade phosphoric acid was purchased from Fisher Scientific (Fair Lawn, NJ, USA). Ultrapure water was obtained using a Milli-Q water purification system from Millipore (Bedford, MA, USA). Standards (purity > 98%) of gallic acid (batch No. M-017-161223), polydatin (batch No. M-017-161223), isoquercetin (batch No. Y-076-161216), hesperidin (batch No. 110721-201818), salvianolic acid B (batch No. D-012-170417) and emodin 8-*O*-*β*-D-glucoside (batch No. D-018-160928) were purchased from Chengdu Herbpurify Co., Ltd. (China).

Saliae Mil Tiorrhizae Radix et Rhizoma (Lot No. 80271101; Lot No. 20190223; Lot No. 1812062), Polygoni Cuspidati Rhizoma et Radix (Lot No. 80511101; Lot No. 20181201; Lot No. 1808094), Crataegi Fructus (Lot No. 82231001; Lot No. 20181220; Lot No. 1810120), Citri Reticulatae Pericarpium (Lot No. 81451002; Lot No. 20190122; Lot No. 1811036), Coicis Semen (Lot No. 82511001; Lot No. 20190214; Lot No. 1809075) and Nelumbinis Folium (Lot No. 83720701; Lot No. 20181227; Lot No. 1809012) were purchased from Beijing San He Co., Ltd. (Beijing, China), Beijing Bencao Fangyuan Pharmaceutical Co., Ltd. (Beijing, China) and Beijing Taiyang Pharmaceutical Industry Co., Ltd. (Beijing, China). After random combination of different batches of herbs, we prepared 10 batches of Danhe granules in the laboratory, as follows: XS190312-01, XS190312-02, XS190318-01, XS190326-01, XS190409-01, XS190409-02, XS190412-01, XS190415-01, XS190420-01 and XS190420-02.

Batch numbers of herbs in ten batches of Danhe granules

DHG Batchs	Saliae Mil Tiorrhizae Radix et Rhizoma	Polygoni Cuspidati Rhizoma et Radix	Crataegi Fructus	Citri Reticulatae Pericarpium	Coicis Semen	Nelumbinis Folium
XS190312-01	80271101	1808094	20181220	81451002	20190214	20181227
XS190312-02	80271101	20181201	82231001	20190122	1809075	20181227
XS190318-01	80271101	20181201	1810120	20190122	1809075	20181227
XS190326-01	20190223	1808094	1810120	20190122	20190214	83720701
XS190409-01	20190223	20181201	1810120	20190122	1809075	83720701
XS190409-02	20190223	20181201	1810120	81451002	82511001	1809012
XS190412-01	1812062	20181201	20181220	1811036	20190214	83720701
XS190415-01	1812062	20181201	20181220	20190122	82511001	20181227
XS190420-01	1812062	20181201	20181220	20190122	82511001	20181227
XS190420-02	1812062	80511101	82231001	20190122	20190214	1809012

2. Instrumentation and chromatographic conditions

Chromatographic analysis was performed on an Agilent 1260 Infinity high-performance liquid chromatograph system (Agilent Technologies, Waldbronn, Germany). The chromatographic data were processed with Agilent Chem Station. Samples separation was based on a ZORBAX Eclipse XDB-C18 column (4.6×250

mm, 5 μm) with column temperature at 30 °C. The mobile phase was composed of solvent A (water-0.1% phosphoric acid) and solvent B (acetonitrile), and the flow rate was kept at 1.0 mL/min. The injection volume was 10 μL and the UV detection wavelength was set at 286 nm. The gradient elution was as follows: 0–55 min, 5–30% B; 55–60 min, 30–60% B; 60–70 min, 60–95% B; 70–75 min, maintained at 95% B.

3. Preparation of standard solutions and DHG samples

Stock solutions of six reference standards were prepared at a concentration of 1.0 mg/mL in methanol, respectively. Serial mixed standard working solutions with different concentrations were prepared through blends and dilutions of the stock solutions with methanol. The stock solutions were filtered through 0.45μm nylon membranes before injection. The DHG sample was weighed 0.20 g and extracted with methanol 50 mL at room temperature by ultrasonic extraction for 30 min, then filtered through 0.45μm nylon membranes before injection.

4. Precision, stability and repeatability validation

Precision validation: The sample solution (batch No: XS190318-01) was injected for 6 times continuously. Salvianolic acid B was used as the correction peak. The results showed that the RSD of the relative retention time of the common peaks was less than 1.20% and the relative peak area RSD was less than 1.52%. Repeatability validation: The sample (batch No: XS190318-01) was used to made six independently sample solutions. Salvianolic acid B was used as the correction peak. After analyzing the six sample solutions, results showed that the RSD of the relative retention time of the common peaks was less than 0.93% and the relative peak area RSD was less than 1.90%. Stability validation: The sample solution (batch No: XS190318-01) was analyzed at 0, 2, 4, 6, 12 and 24 h. Salvianolic acid B was taken as the correction peak. The results showed that the RSD of the relative retention time of the common peaks was less than 2.83% and the relative peak area RSD was less than 2.61%. The above results indicated that the method was with good accuracy, reliable and reproducible for the similarity and quantification analysis of DHG samples.

5. Establishment of chromatographic fingerprint

Based on the Similarity Evaluation System for Chromatographic Fingerprint of TCM (2012 edition), the similarities of the 11 batches of DHG samples (batch XS190312-01 as S1, batch XS190312-02 as S2, batch XS190318-01 as S3, batch XS190326-01 as S4, batch XS190409-01 as S5, batch XS190409-02 as S6, batch XS190412-01 as S7, batch XS190415-01 as S8, batch XS190420-01 as S9 and batch XS190420-02 as S10 and batch D1905007 as S11) was evaluated. After peak-picking, template-matching process, the peaks in the spectra were matched automatically (Shown in Figure S1). The reference template was set finally for spectra peak difference and entire similarity evaluation. The similarities of repeatability were greater than 0.94. The results showed that the preparation process of DHG was reasonable and feasible.

Comparability results of reproducibility of DHG samples

	S1	S2	S3	S4	S5	S6	S7	S8	S9	S10	S11	Reference Fingerprint
S1	1	0.994	0.939	0.995	0.997	0.996	0.993	0.997	0.995	0.916	0.916	0.996
S2	0.994	1	0.953	0.992	0.992	0.992	0.99	0.992	0.991	0.929	0.931	0.997
S3	0.939	0.953	1	0.921	0.923	0.922	0.915	0.924	0.919	0.993	0.981	0.953
S4	0.995	0.992	0.921	1	0.999	0.998	0.999	0.999	0.997	0.929	0.932	0.995
S5	0.997	0.992	0.923	0.999	1	0.999	0.998	0.996	0.999	0.914	0.917	0.995
S6	0.996	0.992	0.922	0.998	0.999	1	0.999	0.999	0.998	0.952	0.927	0.995
S7	0.993	0.99	0.915	0.999	0.998	0.999	1	0.998	0.996	0.933	0.933	0.994
S8	0.997	0.992	0.924	0.999	0.996	0.999	0.998	1	0.999	0.896	0.92	0.995
S9	0.995	0.991	0.919	0.997	0.999	0.998	0.996	0.999	1	0.949	0.928	0.995
S10	0.916	0.929	0.993	0.929	0.914	0.952	0.933	0.896	0.949	1	0.922	0.958
S11	0.916	0.931	0.981	0.932	0.917	0.927	0.933	0.92	0.928	0.922	1	0.944
Reference Fingerprint	0.996	0.997	0.953	0.995	0.995	0.995	0.994	0.995	0.995	0.958	0.944	1

6. Quantitative analysis

Under the above chromatographic conditions, based on the comparison of the standard references, six main HPLC chromatographic peaks in DHG sample were identified. In addition, the quantitative analysis of the six compounds in the DHG sample (batch D1905007) which was used in the pharmacological study was carried out. The results are as follows: the contents of gallic acid, polydatin, isoquercetin, hesperidin, salvianolic acid B and emodin-8-*O*-*β*-D-glucoside were 7.09 mg/g, 4.37 mg/g, 5.39 mg/g, 9.62 mg/g, 13.09 mg/g, and 2.15 mg/g, respectively. The chromatograms of mixed standards and DHG sample are listed in Supplementary Figure S1.

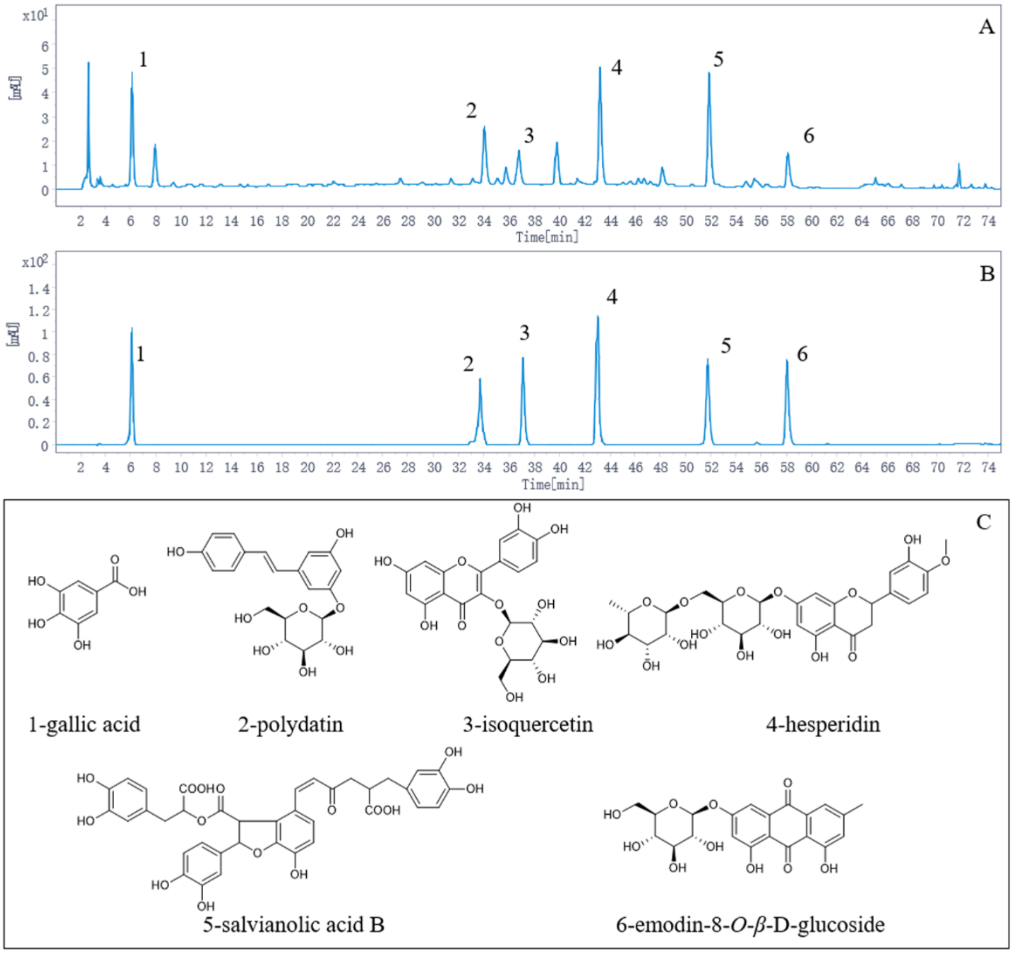

Figure S1. The chromatograms of DHG and standards by HPLC. Chromatogram profiles of DHG (A); Chromatogram profiles of standards (B); Chemical structures of the main compounds in DHG samples (C). The main components of DHG in the chromatograms are as follows: 1-gallic acid, 2-polydatin, 3-isoquercetin, 4-hesperidin, 5-salvianolic acid B and 6-emodin-8-*O*-*β*-D-glucoside.

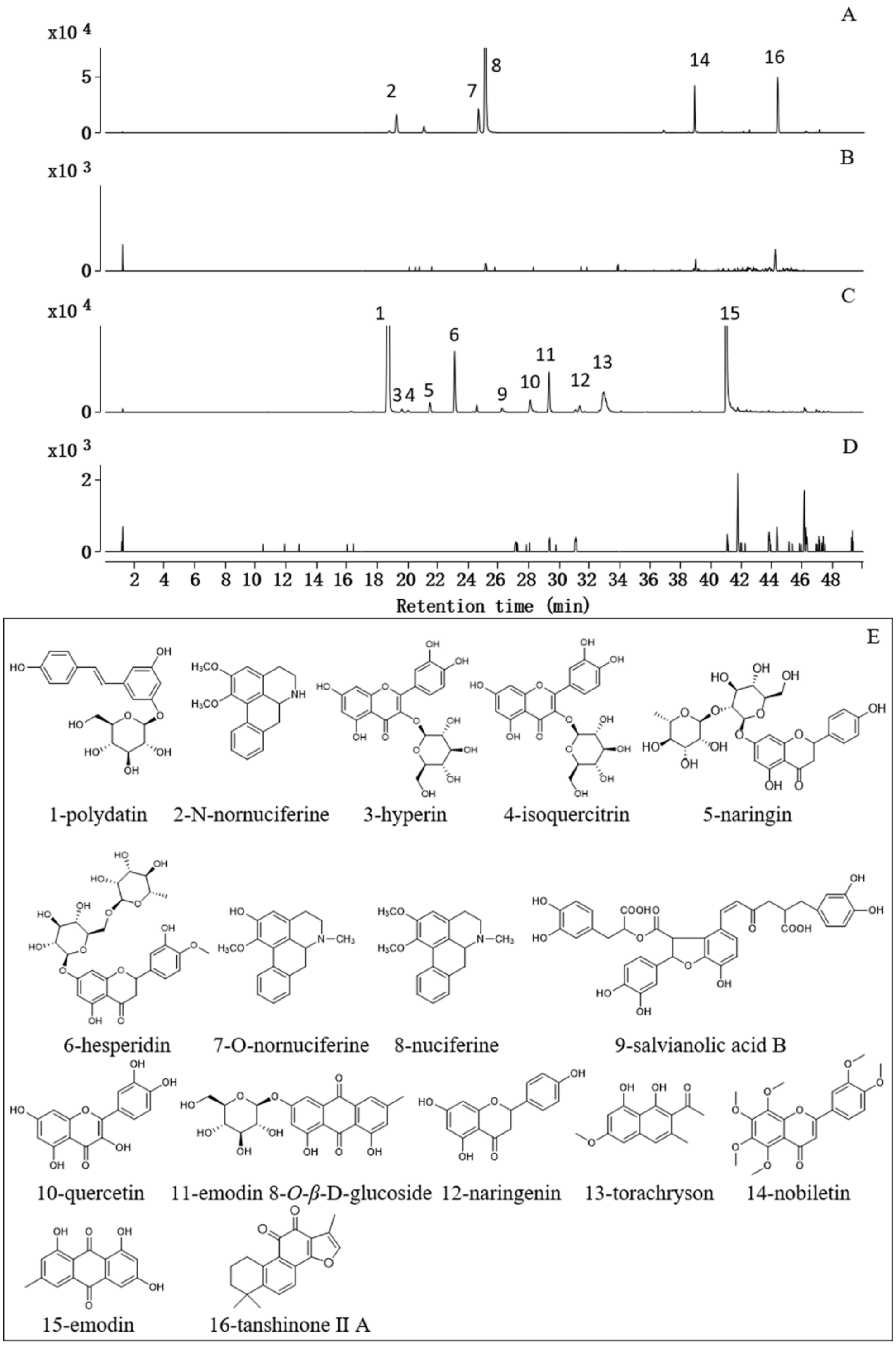

Figure S2. The extracted ion chromatograms (EICs) of 16 ingredients identified in hamster serum; DHG group (A) and blank group (B) in positive ion mode; DHG group (C) and blank group (D) in negative ion mode (The figures A and C were magnified to show those peaks with smaller area). Chemical structures of the main compounds absorbed into blood after DHG taken orally (E).

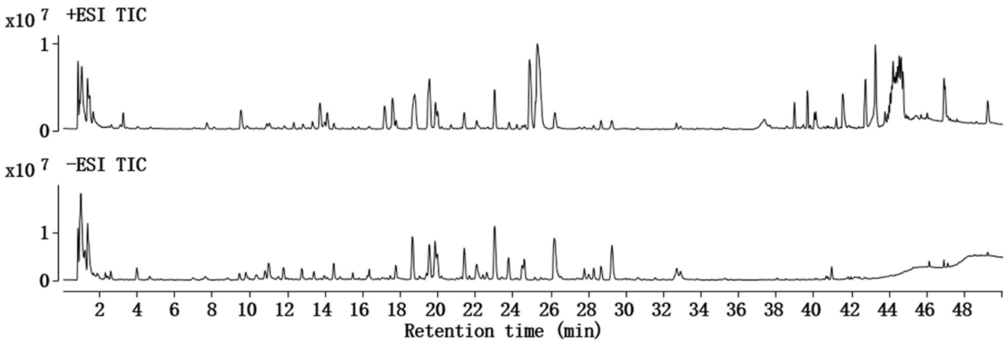

Figure S3. The total ion chromatograms of DHG sample

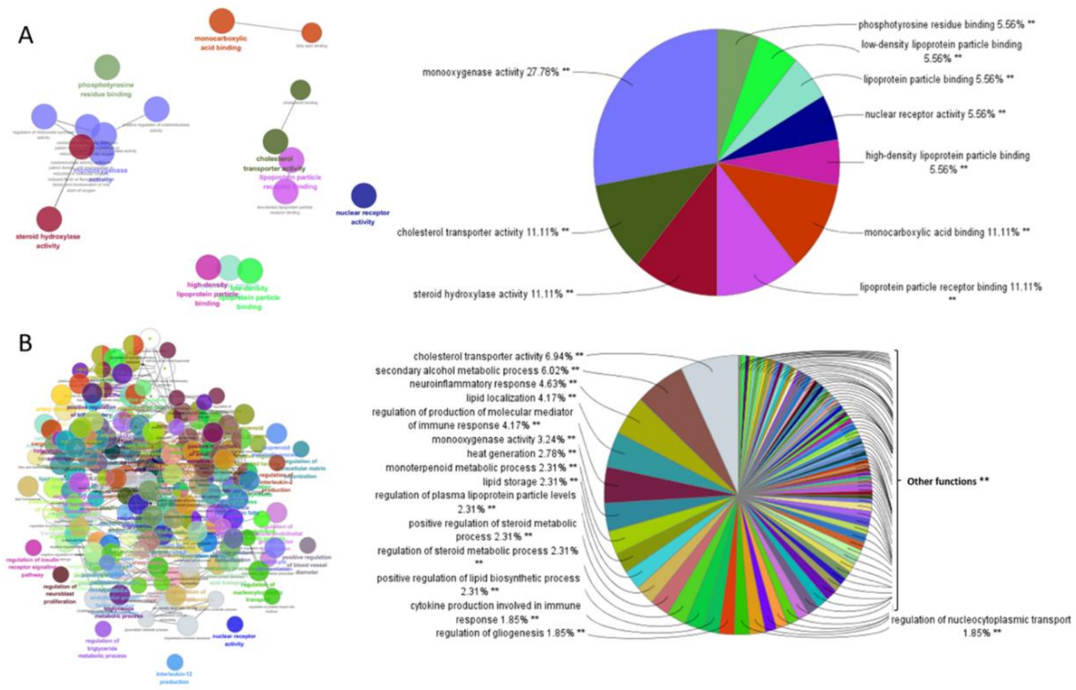

Figure S4. GO enrichment analysis of the targets. The nodes represent GO terms with significant enrichment. The node pie charts represent the molecular function and biological process analysis of these targets. (A) Representative-enriched molecular function relative to the targets. (B) Representative-enriched biological processes relative to the targets

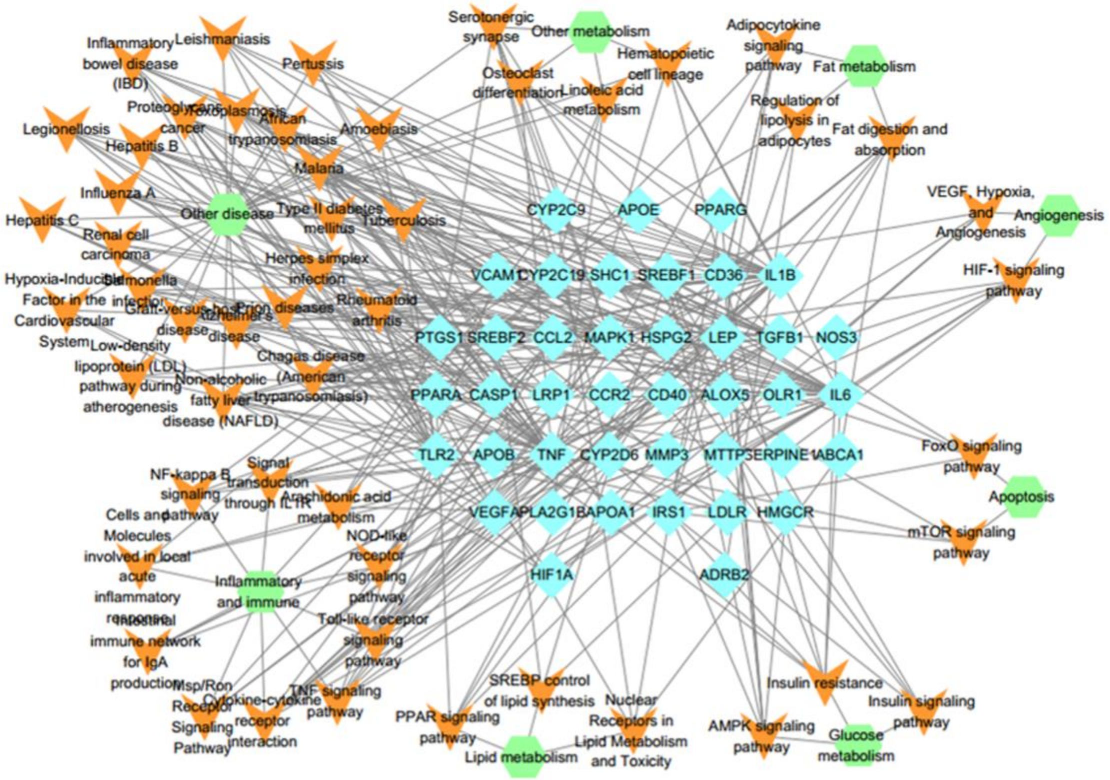

Figure S5. T-P network. The T-P network was constructed by linking targets and their related pathways classified into 8 groups based on pathway analysis. The nodes represent targets (rhombus), pathways (v-shaped) and different groups (hexagon)

The detailed process of identification of serum ingredients

Ten compounds including polydatin, hyperin, hesperidin, salvianolic acid b, nuciferine, quercetin, emodin 8-O-β-D-glucoside, nobiletin, emodin and tanshinone II-A were identified by comparing with reference standards. In addition, other six compounds were identified based on consulting with literatures and fragment ions analysis. Compound 2 and compound 7 are isomers, exhibiting the $[M+H]^+$ ion at *m/z* 282.1495 ($C_{18}H_{19}NO_2$). The differences between them are that compound 7 would produce obvious fragment ion at *m/z* 265 and compound 7 exhibited a longer retention time on a chromatographic column than compound 2. Then, compound 2 and compound 7 were identified as n-nornuciferine and o-nornuciferine, respectively, by consulting with literatures (Deng et al., 2016; Luo et al., 2005). Compound 4, the isomer of hyperin (compound 3), showed the $[M-H]^-$ ion at *m/z* 463.0874 and similar fragment ions at *m/z* 300.0282, *m/z* 271.0255, *m/z* 255.0305, *m/z* 243.0307, *m/z* 227.0355 and *m/z* 151.0044. But compound 4 exhibited a longer retention time on a chromatographic column than hyperin and was identified by consulting with literature (Qiao et al., 2014). Compound 5 showed the $[M-H]^-$ ion at *m/z* 579.1719 and fragment ions at *m/z* 271.0619 $[M-H-Glu-Rha]^-$, *m/z* 227.0722 $[M-H-Glu-Rha-CO_2]^-$, *m/z* 151.0044 $[M-H-Glu-Rha-C_8H_8O]^-$, *m/z* 119.0510 $[M-H-Glu-Rha-CO_2-C_7H_4O_4]^-$ and *m/z* 107.0148$[M-H-Glu-Rha-CO_2-C_7H_4O_4-C]^-$. Therefore, compound 5 was identified as naringin (Ye et al., 2014). Compound 12 showed the $[M-H]^-$ ion at *m/z* 271.0616 and fragment ions at *m/z* 151.0042 $[M-H-C_8H_8O]^-$, *m/z* 119.0508 $[M-H-C_7H_4O_4]^-$ and *m/z* 107.0144 $[M-H-C_7H_4O_4-C]^-$. Then, the compound 12 was deduced to naringenin (Sun et al., 2010). Compound 13 exhibited the $[M-H]^-$ ion at *m/z* 245.0824 and fragment ions at *m/z* 230.0594 $[M-H-CH_3]^-$, *m/z* 215.0362 $[M-H-CH_3-CH_3]^-$, *m/z* 187.0396 $[M-H-CH_3-CH_3-CO]^-$, and *m/z* 159.0458 $[M-H-CH_3-C_3H_3O_2]^-$. Therefore, compound 13 was identified as torachryson (Pan et al., 2015). The information of the proposed fragment behaviors of the 16 compounds was also summarized in the following figures.

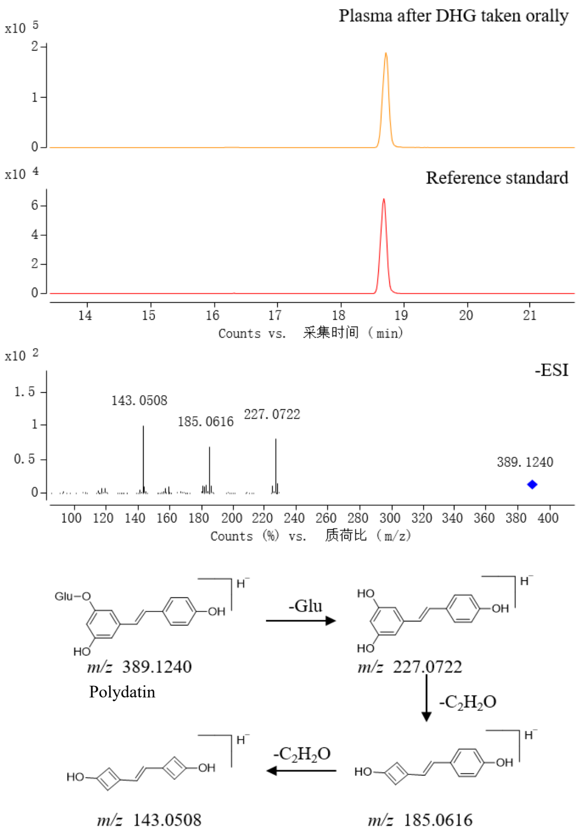

Plasma after DHG taken orally
Reference standard
Counts vs. 采集时间（min）
-ESI
143.0508
185.0616
227.0722
389.1240
Counts (%) vs. 质荷比（m/z）
Glu–O
-Glu
m/z 389.1240
Polydatin
m/z 227.0722
-C2H2O
m/z 143.0508
m/z 185.0616

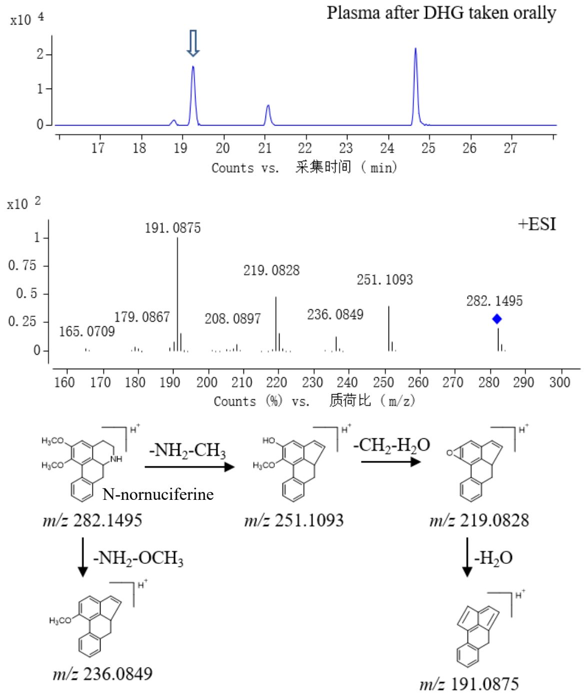

Plasma after DHG taken orally
Counts vs. 采集时间（min）
+ESI
191.0875
219.0828
251.1093
282.1495
179.0867
208.0897
236.0849
165.0709
Counts (%) vs. 质荷比（m/z）
-NH2-CH3
-CH2-H2O
N-nornuciferine
m/z 282.1495
m/z 251.1093
m/z 219.0828
-NH2-OCH3
-H2O
m/z 236.0849
m/z 191.0875

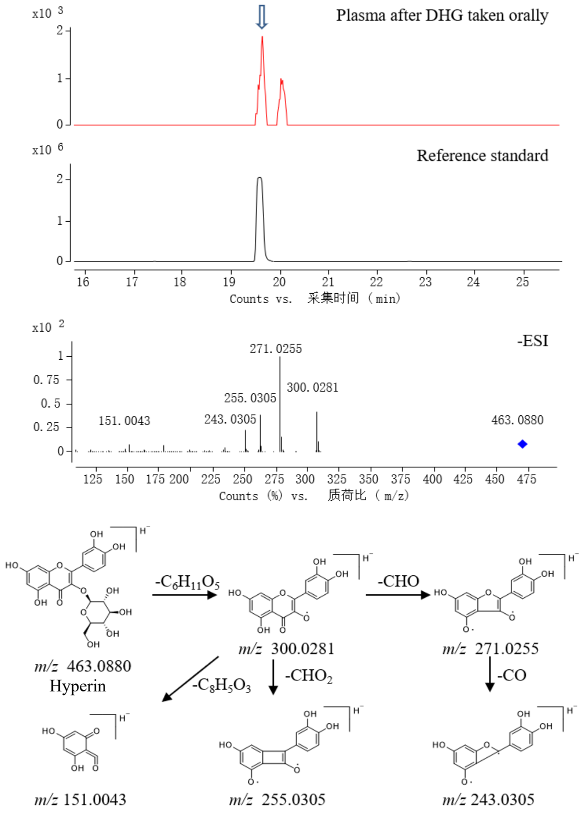

Plasma after DHG taken orally
Reference standard
Counts vs. 采集时间（min）
-ESI
271.0255
300.0281
255.0305
243.0305
151.0043
463.0880
Counts (%) vs. 质荷比（m/z）
-C6H11O5
-CHO
m/z 300.0281
m/z 271.0255
m/z 463.0880
Hyperin
-C8H5O3
-CHO2
-CO
m/z 151.0043
m/z 255.0305
m/z 243.0305

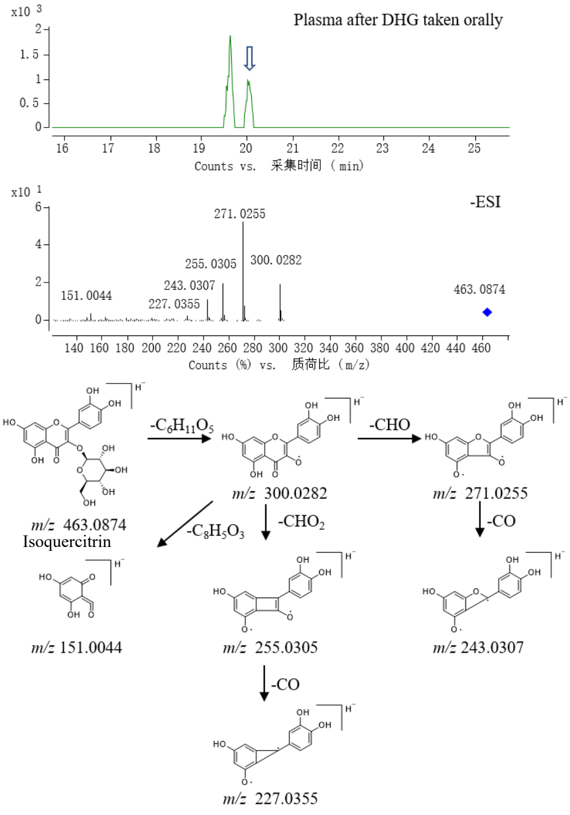

Plasma after DHG taken orally
Counts vs. 采集时间（min）
-ESI
271.0255
255.0305
300.0282
243.0307
151.0044
227.0355
463.0874
Counts (%) vs. 质荷比（m/z）
-C6H11O5
-CHO
m/z 300.0282
m/z 271.0255
m/z 463.0874
Isoquercitrin
-C8H5O3
-CHO2
-CO
m/z 151.0044
m/z 255.0305
m/z 243.0307
-CO
m/z 227.0355

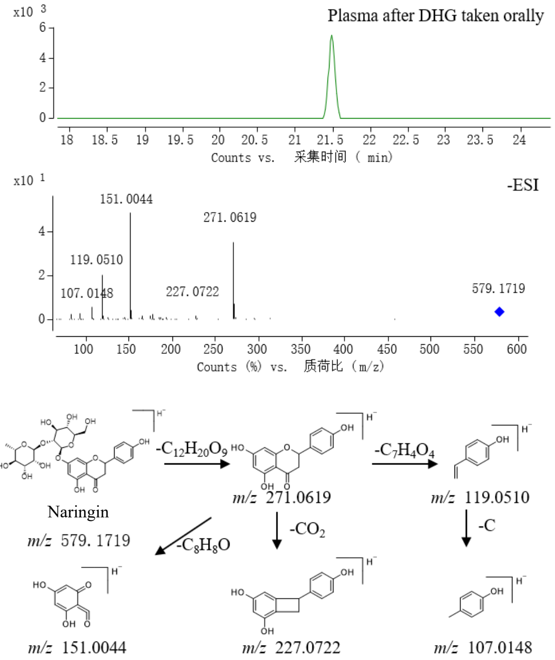

Plasma after DHG taken orally
x10 3
Counts vs. 采集时间（min）
x10 1
-ESI
151.0044
271.0619
119.0510
107.0148
227.0722
579.1719
Counts (%) vs. 质荷比（m/z）
$-C_{12}H_{20}O_9$
$-C_7H_4O_4$
Naringin
m/z 579.1719
m/z 271.0619
m/z 119.0510
$-C_8H_8O$
$-CO_2$
-C
m/z 151.0044
m/z 227.0722
m/z 107.0148

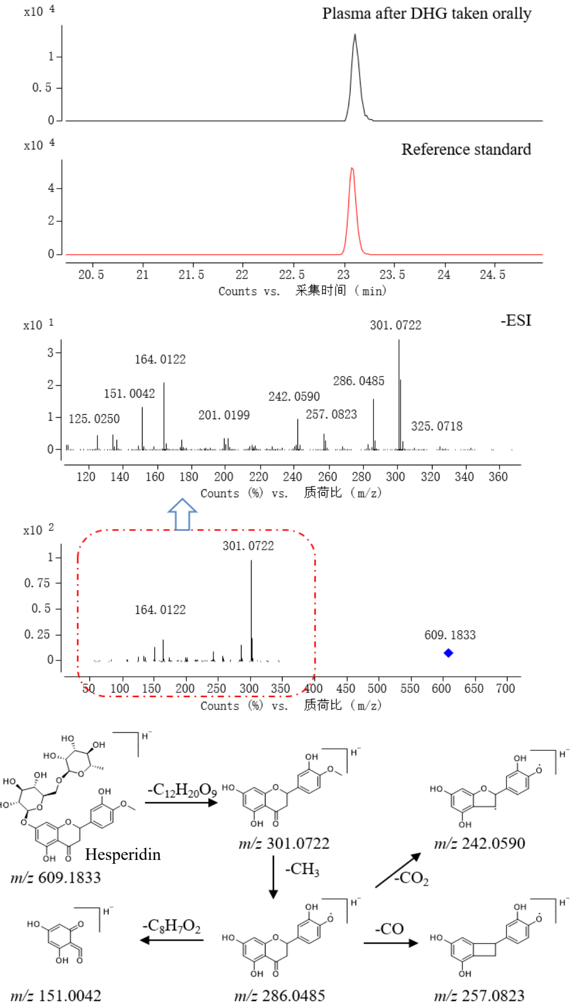

x10 4
Plasma after DHG taken orally
x10 4
Reference standard
Counts vs. 采集时间（min）
x10 1
-ESI
301.0722
164.0122
151.0042
125.0250
201.0199
242.0590
286.0485
257.0823
325.0718
Counts (%) vs. 质荷比（m/z）
x10 2
301.0722
164.0122
609.1833
Counts (%) vs. 质荷比（m/z）
$-C_{12}H_{20}O_9$
Hesperidin
m/z 609.1833
m/z 301.0722
m/z 242.0590
$-CH_3$
$-CO_2$
$-C_8H_7O_2$
-CO
m/z 151.0042
m/z 286.0485
m/z 257.0823

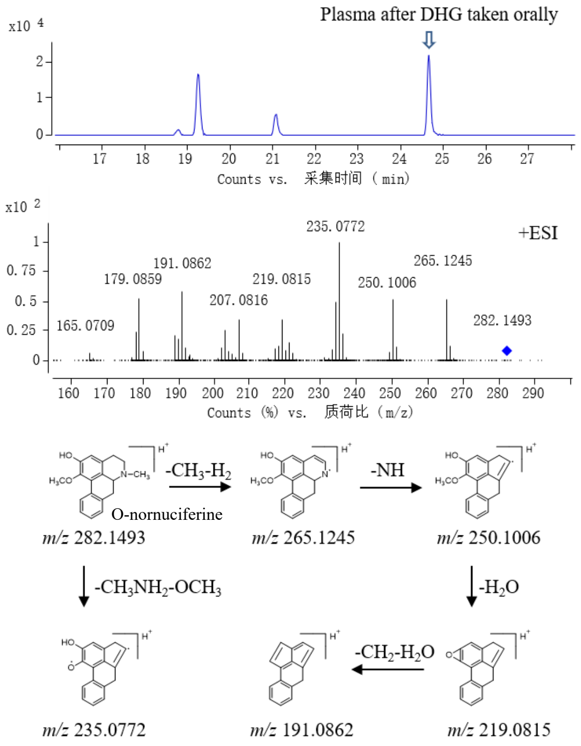
Plasma after DHG taken orally
x10 4
Counts vs. 采集时间（min）
x10 2
+ESI
165. 0709
179. 0859
191. 0862
207. 0816
219. 0815
235. 0772
250. 1006
265. 1245
282. 1493
Counts (%) vs. 质荷比（m/z）
O-nornuciferine
-CH3-H2
-NH
-CH3NH2-OCH3
-H2O
-CH2-H2O
m/z 282.1493
m/z 265.1245
m/z 250.1006
m/z 235.0772
m/z 191.0862
m/z 219.0815

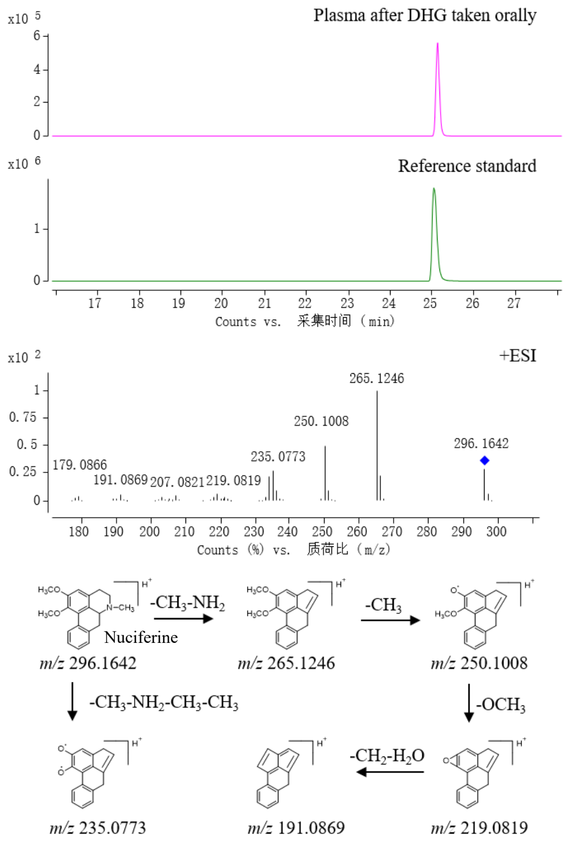
Plasma after DHG taken orally
x10 5
Reference standard
x10 6
Counts vs. 采集时间（min）
x10 2
+ESI
179. 0866
191. 0869
207. 0821
219. 0819
235. 0773
250. 1008
265. 1246
296. 1642
Counts (%) vs. 质荷比（m/z）
Nuciferine
-CH3-NH2
-CH3
-CH3-NH2-CH3-CH3
-OCH3
-CH2-H2O
m/z 296.1642
m/z 265.1246
m/z 250.1008
m/z 235.0773
m/z 191.0869
m/z 219.0819

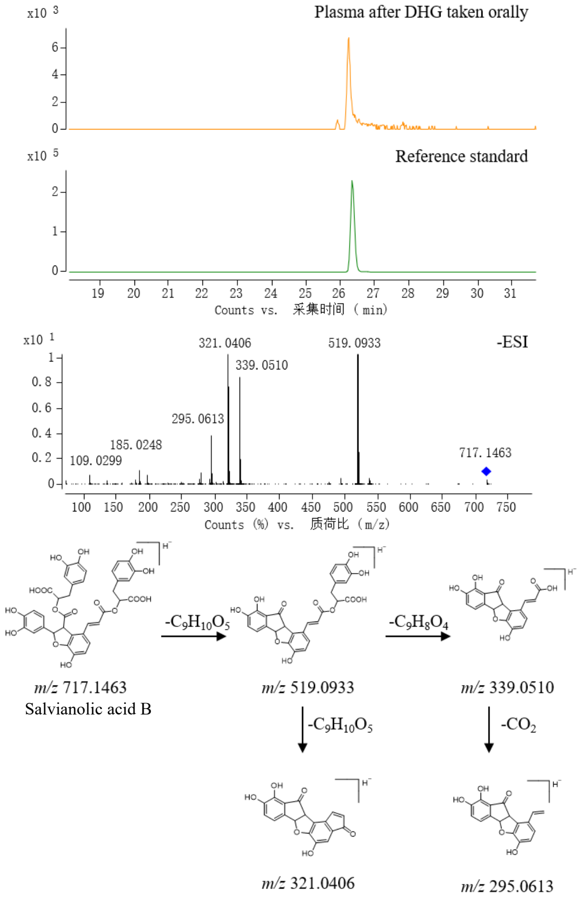

Plasma after DHG taken orally
Reference standard
Counts vs. 采集时间（min）
-ESI
321.0406
339.0510
519.0933
295.0613
185.0248
109.0299
717.1463
Counts (%) vs. 质荷比（m/z）
-C9H10O5
-C9H8O4
m/z 717.1463
Salvianolic acid B
m/z 519.0933
m/z 339.0510
-C9H10O5
-CO2
m/z 321.0406
m/z 295.0613

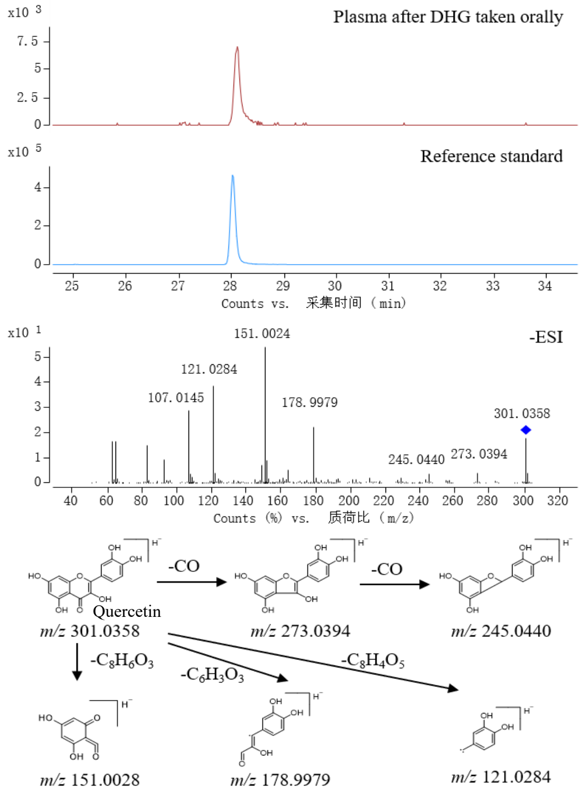

Plasma after DHG taken orally
Reference standard
Counts vs. 采集时间（min）
-ESI
151.0024
121.0284
107.0145
178.9979
301.0358
245.0440
273.0394
Counts (%) vs. 质荷比（m/z）
-CO
-CO
Quercetin
m/z 301.0358
m/z 273.0394
m/z 245.0440
-C8H6O3
-C6H3O3
-C8H4O5
m/z 151.0028
m/z 178.9979
m/z 121.0284

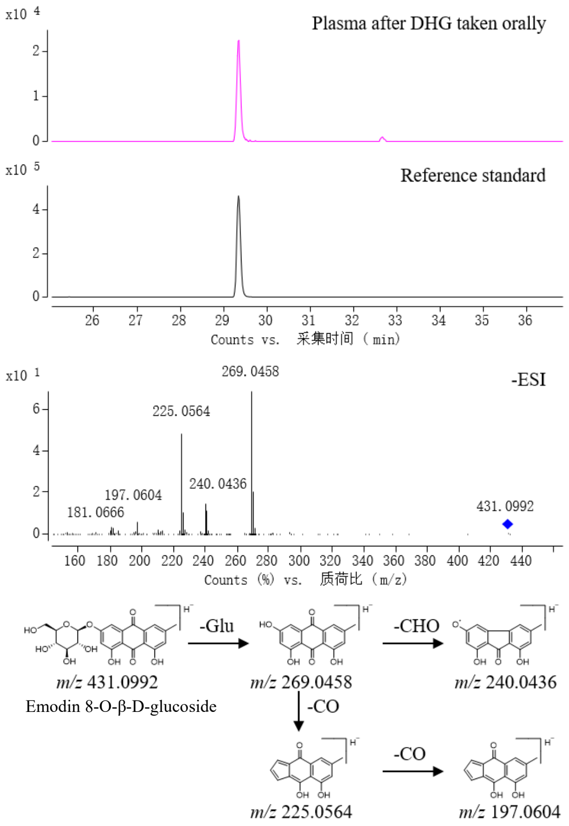
Plasma after DHG taken orally
Reference standard
Counts vs. 采集时间（min）
-ESI
269.0458
225.0564
240.0436
197.0604
181.0666
431.0992
Counts (%) vs. 质荷比（m/z）
-Glu
-CHO
-CO
m/z 431.0992
Emodin 8-O-β-D-glucoside
m/z 269.0458
m/z 240.0436
m/z 225.0564
m/z 197.0604

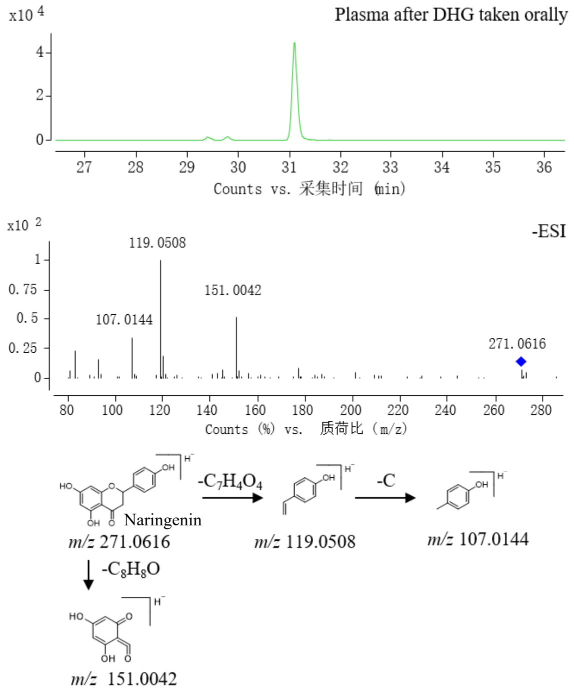
Plasma after DHG taken orally
Counts vs. 采集时间（min）
-ESI
119.0508
151.0042
107.0144
271.0616
Counts (%) vs. 质荷比（m/z）
-C7H4O4
-C
Naringenin
m/z 271.0616
m/z 119.0508
m/z 107.0144
-C8H8O
m/z 151.0042

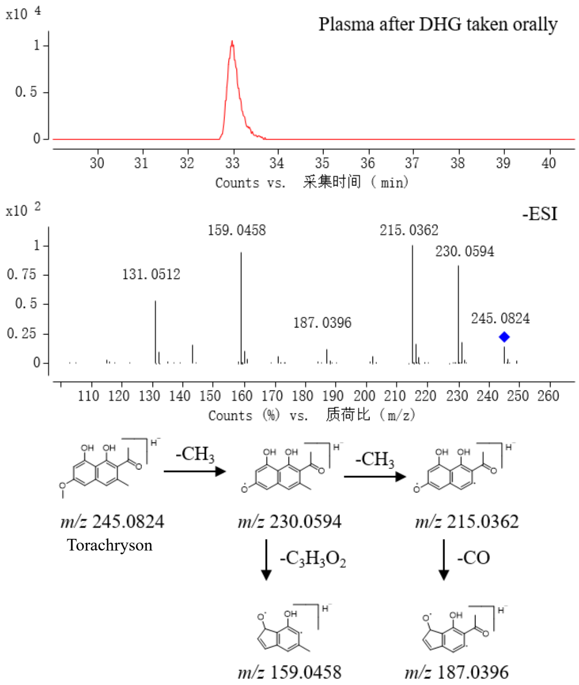

Plasma after DHG taken orally
Counts vs. 采集时间（min）
-ESI
131.0512
159.0458
187.0396
215.0362
230.0594
245.0824
Counts (%) vs. 质荷比（m/z）
-CH3
-CH3
m/z 245.0824
Torachryson
m/z 230.0594
m/z 215.0362
-C3H3O2
-CO
m/z 159.0458
m/z 187.0396

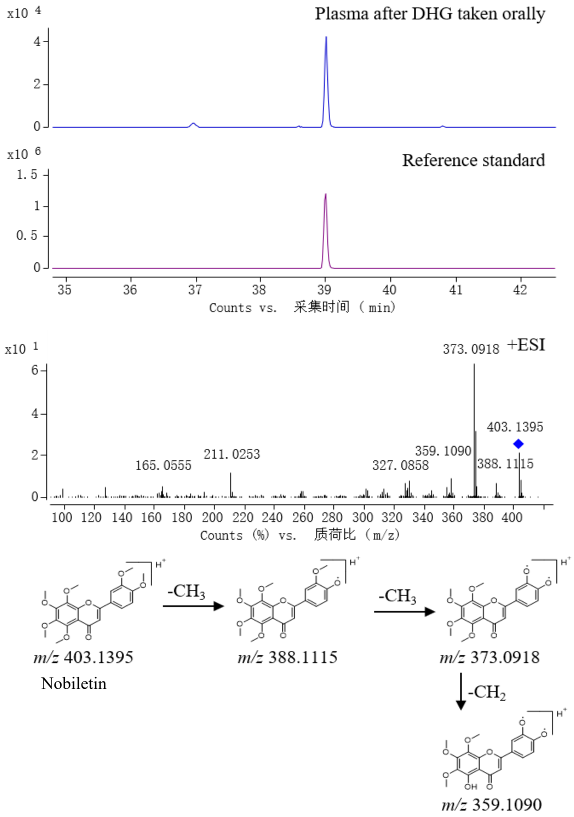

Plasma after DHG taken orally
Reference standard
Counts vs. 采集时间（min）
+ESI
373.0918
403.1395
359.1090
388.1115
327.0858
211.0253
165.0555
Counts (%) vs. 质荷比（m/z）
-CH3
-CH3
m/z 403.1395
Nobiletin
m/z 388.1115
m/z 373.0918
-CH2
m/z 359.1090

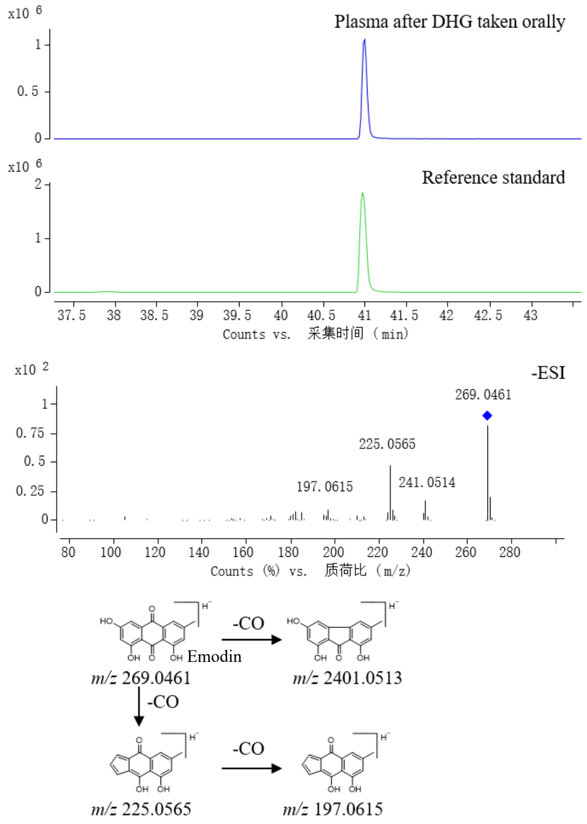

x10 6
Plasma after DHG taken orally
x10 6
Reference standard
Counts vs. 采集时间（min）
x10 2
-ESI
269.0461
225.0565
241.0514
197.0615
Counts (%) vs. 质荷比（m/z）
-CO
Emodin
m/z 269.0461
m/z 2401.0513
-CO
-CO
m/z 225.0565
m/z 197.0615

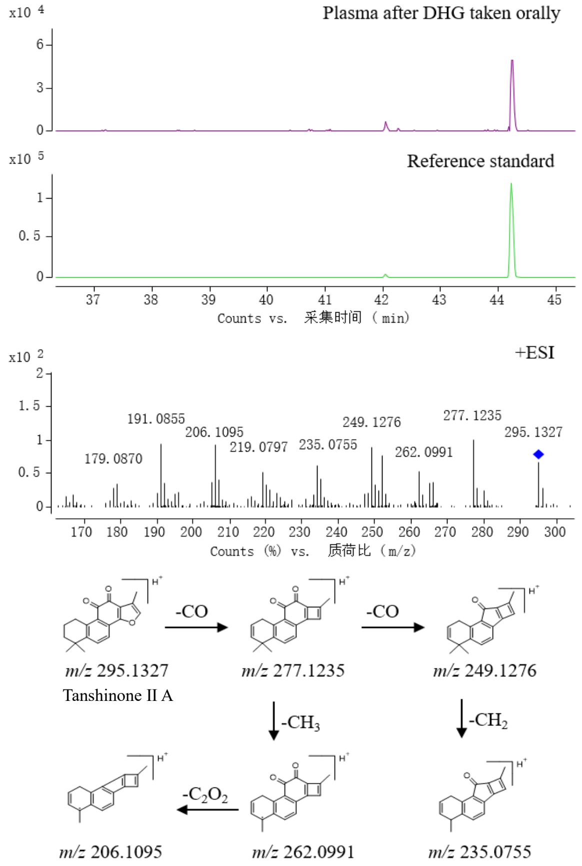

x10 4
Plasma after DHG taken orally
x10 5
Reference standard
Counts vs. 采集时间（min）
x10 2
+ESI
191.0855
206.1095
219.0797
235.0755
249.1276
262.0991
277.1235
295.1327
179.0870
Counts (%) vs. 质荷比（m/z）
-CO
-CO
m/z 295.1327
Tanshinone II A
m/z 277.1235
m/z 249.1276
-CH3
-CH2
-C2O2
m/z 206.1095
m/z 262.0991
m/z 235.0755

Sun, G., Qian, D., Duan, J. A., Li, X., Wan, J., and Guo, J. (2010). UPLC-Q-TOF-MS analysis of naringin and naringenin and its metabolites in rat plasma after intragastrical administration of alcohol extract of exocarpium Citri grandis. *China J. Chin. Mater. Med.* 35, 1580-1585. doi: 10.4268/cjcmm20101217

Ye, X., Cao, D., Zhao, X., Song, F., Huang, Q., Fan, G., and Wu, F. (2014). Chemical fingerprint and metabolic profile analysis of Citrus reticulate 'Chachi'decoction by HPLC-PDA-IT-MSn and HPLC-Quadrupole-Orbitrap-MS method. *J Chromatogr B*. 970, 108-120. doi: 10.1016/j.jchromb.2014.06.035

Luo, X., Chen, B., Liu, J., and Yao, S. (2005). Simultaneous analysis of N-nornuciferine, O-nornuciferine, nuciferine, and roemerine in leaves of Nelumbo nucifera Gaertn by high-performance liquid chromatography–photodiode array detection–electrospray mass spectrometry. *Anal Chim Acta.* 538, 129-133. doi: 10.1016/j.aca.2005.01.066

Deng, X., Zhu, L., Fang, T., Vimolmangkang, S., Yang, D., Ogutu, C., Liu, Y., and Han, Y. (2016). Analysis of isoquinoline alkaloid composition and wound-induced variation in Nelumbo using HPLC-MS/MS. *J Agric Food Chem*. 64, 1130-1136. doi: 10.1021/acs.jafc.5b06099

Qiao, X., Wu S., Qi, X., Feng, J., and Xiao, X. (2014). Identification of chemical constituents in Crataegus pinnatifida var. major by UPLC/ESI-TOF/MS. *Drugs and Clinic*, 29, 120-124. doi: 10.7501/j.issn.1674-5515.2014.02.003

Pan, Z., Liang, H., Liang, C., and Xu, W. (2015). An ultra-high-pressure liquid chromatography/linear ion trap-Orbitrap mass spectrometry method coupled with a diagnostic fragment ions-searching-based strategy for rapid identification and characterization of chemical components in Polygonum cuspidatum. *Chin. J. Chromatogr.* 33, 22-28. doi: 10.3724/sp.j.1123.2014.07006
